# Polyhedral Dicobaltadithiaboranes and Dicobaltdiselenaboranes as Examples of Bimetallic *Nido* Structures without Bridging Hydrogens

**DOI:** 10.3390/molecules28072988

**Published:** 2023-03-27

**Authors:** Amr A. A. Attia, Alexandru Lupan, Robert Bruce King

**Affiliations:** 1 Faculty of Chemistry and Chemical Engineering, Babeş-Bolyai University, 400347 Cluj-Napoca, Romania; amr.attia@ubbcluj.ro; 2 Department of Chemistry and Center for Computational Quantum Chemistry, University of Georgia, Athens, GA 30602, USA

**Keywords:** polyhedral boranes, sulfur, selenium, cobalt, density functional there

## Abstract

The geometries and energetics of the *n*-vertex polyhedral dicobaltadithiaboranes and dicobaltadiselenaboranes Cp_2_Co_2_E_2_B*_n_*_−4_H*_n_*_−4_ (E = S, Se; *n* = 8 to 12) have been investigated via the density functional theory. Most of the lowest-energy structures in these systems are generated from the (*n* + 1)-vertex most spherical *closo* deltahedra by removal of a single vertex, leading to a tetragonal, pentagonal, or hexagonal face depending on the degree of the vertex removed. In all of these low-energy structures, the chalcogen atoms are located at the vertices of the non-triangular face. Alternatively, the central polyhedron in most of the 12-vertex structures can be derived from a Co_2_E_2_B_8_ icosahedron with adjacent chalcogen (E) vertices by breaking the E–E edge and 1 or more E–B edges to create a hexagonal face. Examples of the *arachno* polyhedra with two tetragonal and/or pentagonal faces derived from the removal of two vertices from *isocloso* deltahedra were found among the set of lowest-energy Cp_2_Co_2_E_2_B*_n_*_−4_H*_n_*_−4_ (E = S, Se; *n* = 8 and 12) structures.

## 1. Introduction

**Figure 1:**
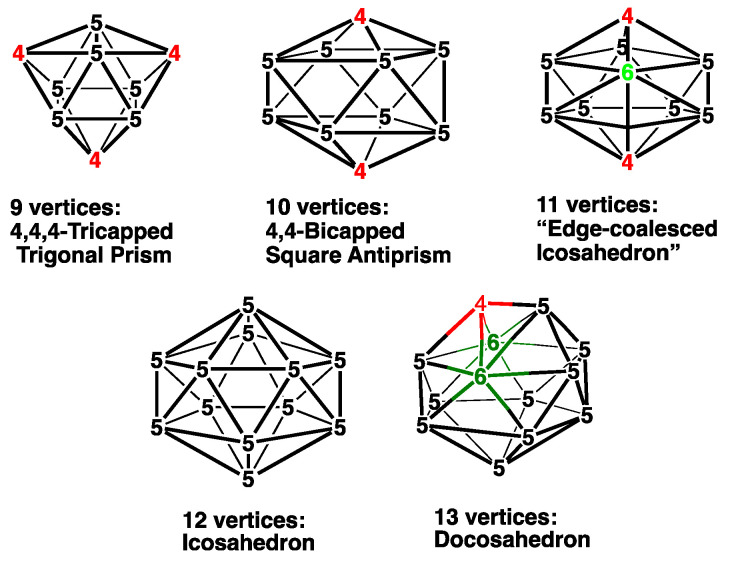
The most spherical *closo* deltahedra having 9 to 13 vertices from which the *nido* structures discussed in this paper are generated. Vertices of degrees 4, 5, and 6 are indicated in red, black, and green, respectively, in Figures 1, 2, and 8.

The structures of the polyhedral borane dianions B*_n_*H*_n_*^2−^ as well as those of the isoelectronic carborane monoanions CB*_n_*_−1_H*_n_*^−^ and neutral dicarbaboranes C_2_B*_n_*_−2_H*_n_* are based on the most spherical deltahedra, which are known as the *closo* deltahedra (Figure 1) [1,2]. Except for the 13-vertex systems, the most spherical deltahedra have exclusively triangular faces with vertex degrees as nearly similar as possible. The 11- and 13-vertex systems contain 1 and 2 degree-6 vertices, respectively, whereas the other *closo* polyhedra have exclusively degree-4 and -5 vertices, for which the degree of a vertex is defined as the number of edges meeting at the vertex in question. The chemical bonding in these polyhedral boranes and carboranes is based on 2*n* + 2 skeletal electrons for an *n*-vertex system, with each BH and CH vertex contributing 2 and 3 skeletal electrons, respectively, after providing an electron for the external B–H or C–H bond. This is a key aspect of the Wade–Mingos rules [3,4,5] relating polyhedral geometry to skeletal electron count. A reasonable chemical bonding model for these deltahedra consists of a resonance hybrid of canonical structures containing a single *n*-center bond composed of orbitals from each of the *n*-vertex atoms overlapping at the center of the polyhedron supplemented by *n* two-center, two-electron bonds on the surface of the polyhedron [6]. This structural model accounts for the 2*n* + 2 skeletal electrons in the stable *closo* deltahedral borane structures. The delocalization implicit in this bonding model, particularly the presence of the multicenter core bond, is consistent with the interpretation of these species as three-dimensional aromatic systems [7,8].

The research groups of Hawthorne [9] and Grimes [10] were the first to show that the boron vertices in these polyhedral boranes could be replaced by transition metal units. The cyclopentadienylcobalt unit (CpCo) was especially useful for this purpose due to the robustness of the linkage between the cyclopentadienyl ring and the cobalt atom. Furthermore, a CpCo vertex is a donor of two skeletal electrons like a BH vertex so that it can replace a BH unit in the polyhedral structures. The research of the Hawthorne group [9] focused on metallaboranes that can be obtained from decaborane (B_10_H_14_) as a precursor, whereas the research of the Grimes group [10] studied metallaboranes obtained from pentaborane (B_5_H_9_) as a precursor. The chemistry of polyhedral boranes and their transition metal derivatives remains of interest even after approximately half a century following the discovery of the original metallaboranes with the possibilities of applications in medicine [11,12] and catalysis [13].

**Figure 2:**
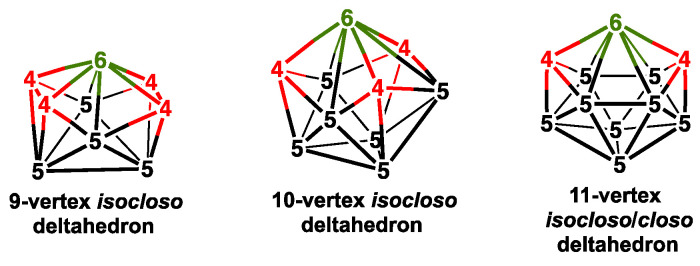
The *isocloso* deltahedra from which the *nido* polyhedra with hexagonal holes are generated.

Further development of metallaborane chemistry, especially by Kennedy and his co-workers, by using a variety of metal vertices, particularly those containing second- and third-row transition metals, resulted in the discovery of alternative so-called *isocloso* deltahedra in 9- and 10-vertex metallaborane structures with a degree-6 vertex for the metal atom (Figure 2) [14,15,16,17]. The 11-vertex *closo* deltahedron can also be an *isocloso* deltahedron since it necessarily has a degree 6 vertex. A reasonable chemical bonding model for an *n*-vertex *isocloso* metallaborane deltahedron consists exclusively of *n* 3-center, 2-electron (3c-2e) bonds in *n* of the 2*n* − 4 faces of the deltahedron without the *n*-center core bond found in the *closo* chemical bonding [18]. Thus, the *isocloso* metallaboranes are 2*n* rather than 2*n* + 2 skeletal electron systems.

Electron-richer polyhedral borane derivatives with *n*-vertices are obtained by removing a vertex from an (*n* + 1)-vertex *closo* or *isocloso* deltahedron to give a so-called *n*-vertex *nido* polyhedron. Such a vertex removal process creates a smaller polyhedron with a non-triangular face at the site of vertex removal. This non-triangular face can be regarded as a “hole” in an otherwise triangulated polyhedral surface. Williams [2] introduced a convenient notation for *nido* polyhedra as ni-*n*〈*h*〉, where *n* is the number of vertices, and *h* is the number of vertices in the non-triangular face as indicated by a Roman numeral. For example, by using this notation, the square pyramid can be designated as a ni-5〈IV〉 polyhedron. Since the original (*n* + 1)-vertex *closo* deltahedron has 2(*n* + 1) + 2 skeletal electrons, the *n-*vertex *nido* polyhedron derived from it has 2*n* + 4 skeletal electrons.

**Figure 3:**
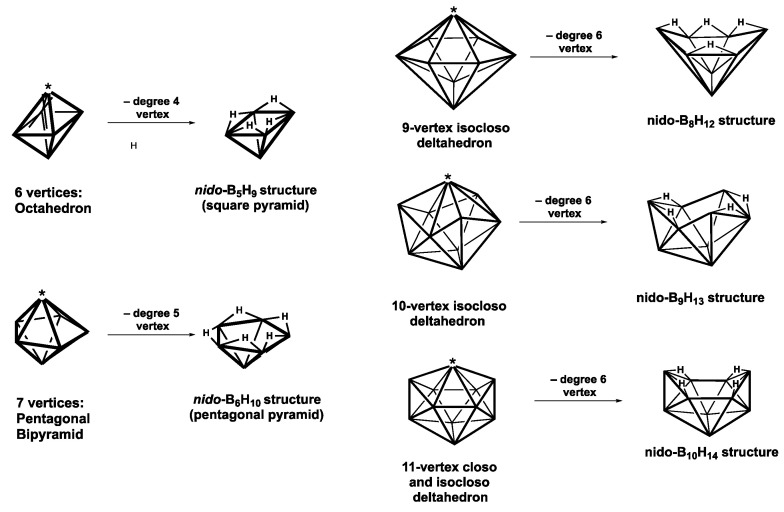
Generation of the binary B*_n_*H*_n+_*_4_ borane structures from *closo* and *isocloso* deltahedra by the removal of the starred vertices.

The discovery of neutral binary boranes B*_n_*H*_n+_*_4_ (*n* = 2, 5, 6, 8, 9, 10) exhibiting *nido* structures, of which B_10_H_14_ is the most stable [19,20], predates that of the more stable *closo* boranes by going back to the original boron hydride syntheses of Stock. The structures of B_5_H_9_ and B_6_H_10_ are derived by the removal of a degree-4 vertex from an octahedron and a degree-5 vertex from a pentagonal bipyramid, respectively, thereby generating a square and a pentagonal face, respectively (Figure 3). The structures of the larger B*_n_*H*_n+_*_4_ (*n* = 8, 9, 10) boranes are derived by removal of the unique degree-6 vertex from the corresponding (*n* + 1) vertex *isocloso* deltahedron. The polyhedral frameworks of all of the B*_n_*H*_n_*_+4_ boranes (*n* = 5, 6, 8, 9, 10) all have *n* BH vertices with the four “extra” hydrogen atoms bridging the B–B edges surrounding the non-triangular face. A relatively large non-triangular face such as a hexagonal hole versus a pentagonal or tetragonal face provides more space for the four hydrogen atoms bridging the hole B–B edges.

A topic of interest is the design of viable *n*-vertex *nido* borane structures that have the necessary 2*n* + 4 skeletal electrons without the need for bridging hydrogen atoms. Tetracarbaborane structures of the type C_4_B*_n_*_−4_H*_n_* provide the required 2*n* + 4 skeletal electrons. In this connection, species of the type C_4_B*_n_*_−4_R*_n_* (R = H or alkyl) are known experimentally to have such structures with 6 [21,22], 8 [23,24], 10 [25,26,27,28], 11 [29], and 12 [30,31,32,33,34,35,36] vertices. Furthermore, the structures and energetics of the tetracarbaboranes have been studied by using modern density functional theory methods [37].

Generating a *nido* polyhedral borane with the requisite 2*n* + 4 skeletal electrons by using electron-richer carbon atoms in CH vertices to replace boron atoms in BH vertices requires four such carbon heteroatoms in the carborane polyhedron. Using bare sulfur or selenium vertex atoms as heteroatoms in polyhedral borane structures to provide the 2*n* + 4 skeletal electrons for *nido* geometry requires only two heteroatoms in an E_2_B*_n_*_−2_H*_n_*_−2_ (E = S, Se) structure since bare sulfur or selenium vertices are each donors of four skeletal electrons after diverting 2 of their 6 valence electrons to an external lone pair. In this connection, the 11-vertex *nido*-B_9_H_9_E_2_ (E = S, Se) structures have been synthesized and shown via X-ray crystallography to have a central polyhedron obtained via the removal of one vertex from an icosahedron [38].

**Figure 4:**
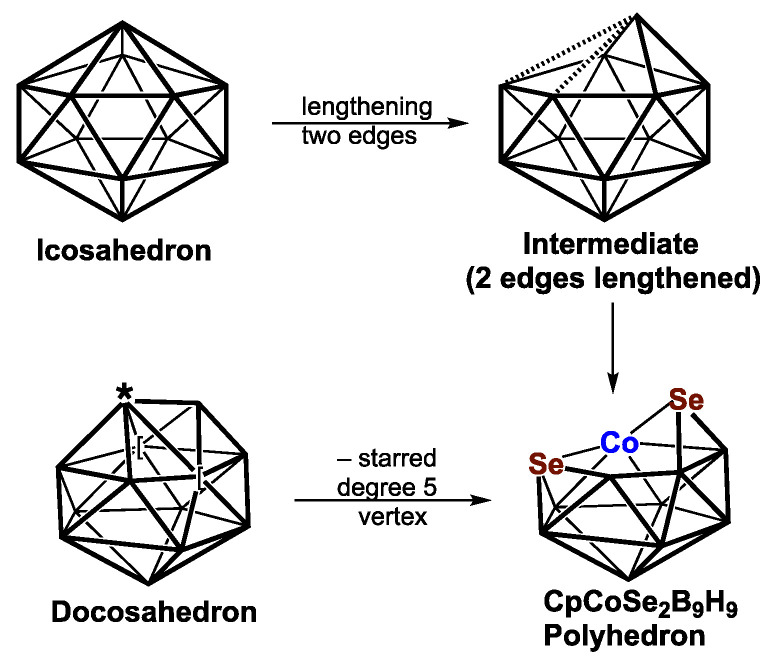
Alternative ways of generating the central 12-vertex polyhedron of the experimentally known CpCoSe_2_B_9_H_9_ from the 12-vertex icosahedron by breaking edges and from the 13-vertex docosahedron by removing the starred degree-5 vertex.

Dithiaboranes and diselenaboranes with one or two CpCo vertices have been synthesized and structurally characterized via X-ray crystallography (Figure 4). The 12-vertex cobaltadiselenaborane CpCoSe_2_B_9_H_9_ has been synthesized and shown to have a ni-13〈VI〉 structure derived via the removal of a degree-6 vertex from a docosahedron, which is the 13-vertex *closo* deltahedron [35]. This structure can also be derived from a central CoSe_2_B_9_ icosahedron with a Se–Se edge by breaking the Se–Se edge and an adjacent Se–B edge to generate an SeCoSeBB pentagonal face, leaving a degree-3 selenium vertex. This structure is shown by density functional theory to be 1 of the 3 lowest-energy structures lying within 1 kcal/mol of each other [39].

**Figure 5:**
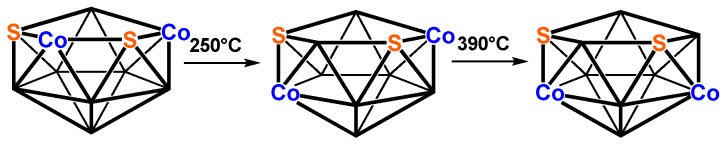
The 11-vertex frameworks suggested for the 3 Cp*_2_Co_2_S_2_B_7_H_7_ isomers isolated by Kang and Sneddon from the reaction between LiCp*, NaS_2_B_7_H_9_, and CoCl_2_.

In total, 3 isomeric structures of the 11-vertex dicobaltadithiaborane Cp*_2_Co_2_S_2_B_7_H_7_ with 2 pentamethylcyclopentadienylcobalt vertices (Cp*Co) have been isolated by Kang and Sneddon [40] from the mixture that was obtained from the reaction between LiCp*, NaS_2_B_7_H_8_, and CoCl_2_ (Figure 5). Attempts to obtain definitive structural information on these species via X-ray crystallography were prevented by disorder problems. However, the poor X-ray data were sufficient to indicate the relative positions of the cobalt atom and 11-vertex Co_2_S_2_B_7_ geometries obtained via removal from a vertex from a central icosahedron. Furthermore, 1 of the 3 structures appears to be an ultimate pyrolysis product at 300 °C. The structures were assigned on the basis of the ^11^B NMR spectra. In addition, trace quantities of a 10-vertex Cp*_2_Co_2_S_2_B_6_H_6_ structure were obtained, which was suggested to have a decaborane-like structure on the basis of its ^11^B NMR.

In view of the difficulty in obtaining definitive structural information on the dicobaltadithiaboranes, we have undertaken density functional theory studies on the complete series of Cp_2_Co_2_E_2_B*_n_*_−4_H*_n_*_−4_ (E = S, Se; *n* = 8, 9, 10, 11, 12) systems with unsubstituted cyclopentadienyl rings. In addition, we have included the Cp*_2_Co_2_S_2_B_7_H_7_ system with pentamethylcyclopentadienyl (Cp*) rings in this study for comparison with the reported experimental data. This theoretical study extends the previous study [36] of the dicobaltadiselenaboranes by introducing a second cobalt atom into the metallaborane polyhedron. This provides an opportunity to assess preferences for a pair of cobalt atoms in these structures to occupy adjacent bonding positions or to be as far removed from each other as possible or something in between. The dicobaltadithiaboranes and dicobaltadiselena boranes are of interest in representing the first examples of *nido* structures without bridging hydrogen atoms containing two transition metal vertices in the underlying polyhedron.

## 2. Results and Discussion

### 2.1. The 11-Vertex Systems Cp_2_Co_2_E_2_B_7_H_7_ (E = S, Se)

**Figure 6:**
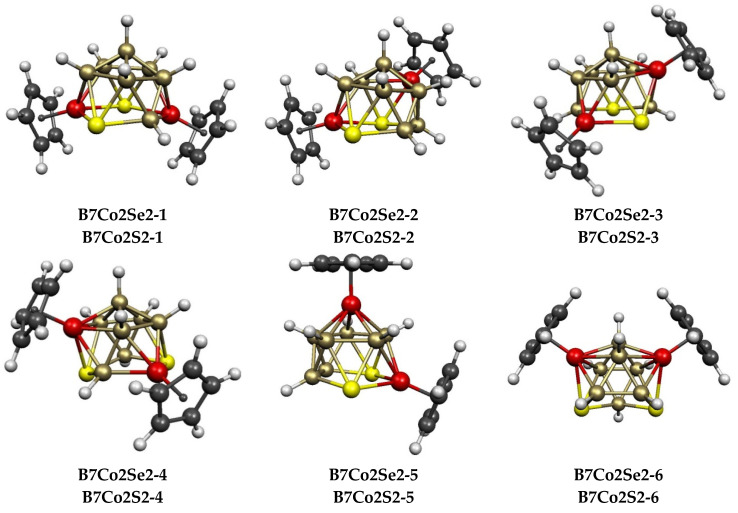

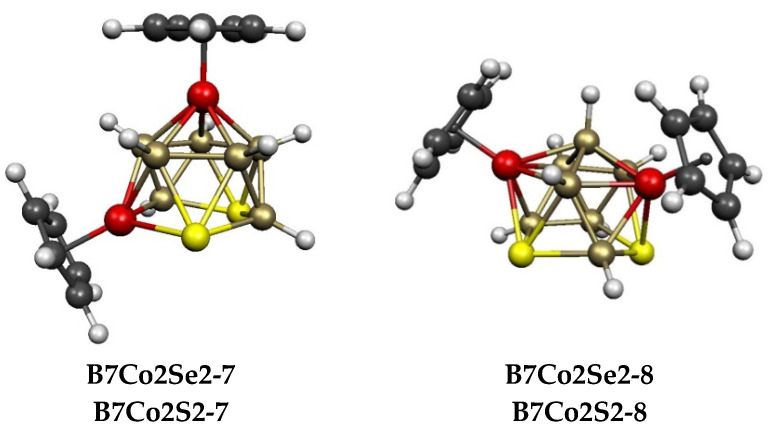
The eight lowest-energy Cp_2_Co_2_E_2_B_7_H_7_ (E = S, Se) structures oriented to have the open pentagonal face at the bottom.

In total, 8 structures were found for each of the 11-vertex Cp_2_Co_2_E_2_B_7_H_7_ (E = S, Se) systems within 15 kcal/mol of the lowest-energy structure (Figure 6 and Table 1). The central ni-11〈V〉 polyhedron in all of the eight structures can be derived from an icosahedron via the removal of one vertex, leaving a pentagonal face that is similar to the dicarbollide anion C_2_B_9_H_12_^−^ that was originally used by Hawthorne and co-workers [9] to synthesize a variety of transition metal complexes having a central MC_2_B_9_ icosahedron. For both Cp_2_Co_2_E_2_B_7_H_7_ (E = S, Se) systems, the lowest-energy structures **B7Co2E2-1** (E = S, Se) have both cobalt atoms and both chalcogen atoms at the surface of the pentagonal face and, thus, at degree-4 vertices. This is the most prevalent example of generating an *n*-vertex *nido* polyhedral borane by the removal of a vertex from an (*n* + 1)-vertex *closo* deltahedral borane. Furthermore, in each of the 8 low-energy Cp_2_Co_2_E_2_B_7_H_7_ structures, both chalcogen atoms are located at the degree-4 pentagonal face vertices rather than at interior degree-5 vertices. This is consistent with the preference of chalcogen atoms for lower degree vertices in polyhedral selena- and thiaboranes.

Three isomeric pentamethylcyclopentadienyl Cp*_2_Co_2_S_2_B_7_H_7_ complexes have been isolated by Kang and Sneddon in small quantities from the reaction between LiCp*, NaS_2_B_7_H_8_, and CoCl_2_ which they designated by the Roman numerals III, IV, and V in their paper (Figure 5) [37]. Extensive disorder prevented complete X-ray structure determinations on these molecules beyond location of the cobalt atoms. On the basis of ^11^B NMR and 2D ^11^B-^11^B COSY NMR, Kang and Sneddon assigned structures analogous to **B7Co2S-1**, **B7Co2S-2**, and **B7Co2S-3** to III, IV, and V, respectively. We found that complete substitution of hydrogen atoms with methyl groups did not affect the relative energy ordering of the 3 lowest-energy structures with the relative energies of the Cp* structures B7Co2S-1*, B7Co2S-2*, and B7Co2S-3* being 0.0, 0.6, and 6.7 kcal/mol, respectively. What is strange is the observation that the Kang/Sneddon isomer III, which has the assigned structure B7Co2S2-1* and has both cobalt atoms as well as both sulfur atoms located on pentagonal face vertices, is converted ultimately upon heating to 300 °C to the Kang/Sneddon isomer V (Figure 5), which was assigned the higher-energy structure B7Co2S2-3*, as it has both cobalt atoms located at degree-5 interior vertices. This is contrary to expectation because pyrolysis, particularly to a temperature as high as 300 °C, would be expected to give a lower-energy isomer rather than a higher-energy isomer. Our theoretical studies cast some doubt about the structure assignments of III, IV, and V that were given by Kang and Sneddon in their 1988 study [37]. Note that the predicted Co^…^Co distances in **B7Co2S-1**, **B7Co2S-2**, and **B7Co2S-3** are all approximately 3.8 to 3.9 Å, so the determination of these distances by an otherwise incomplete X-ray crystallography study on an extensively disordered system would not be sufficient to distinguish between these three structures. The improvements in X-ray crystallography methodology in the 35 years since the Kang/Sneddon report of the 3 Cp*_2_Co_2_S_2_B_7_H_7_ isomers might provide a resolution to this dilemma.

### 2.2. The 12-Vertex Systems Cp_2_Co_2_E_2_B_8_H_8_ (E = S, Se)

**Figure 7:**
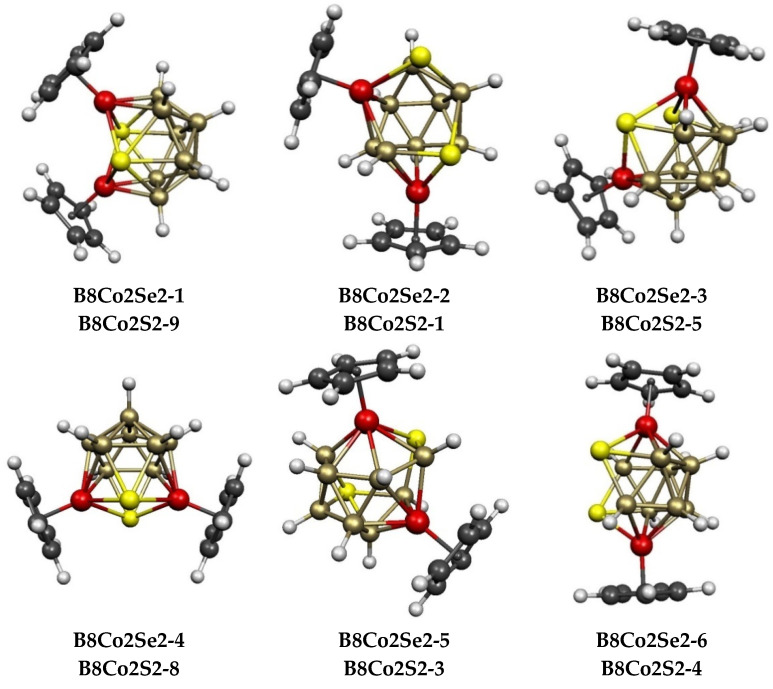

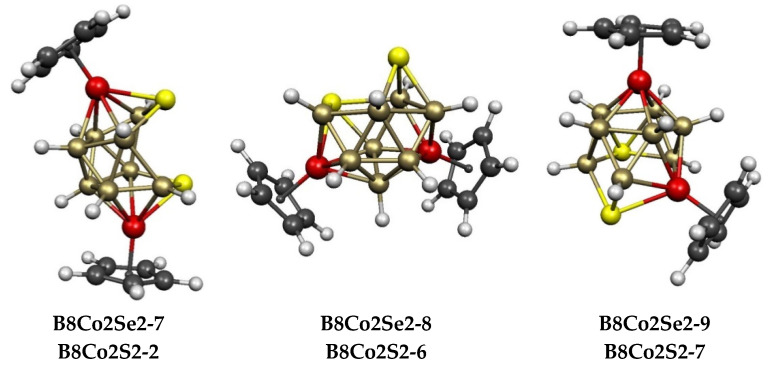
The nine lowest-energy Cp_2_Co_2_E_2_B_8_H_8_ (E = S, Se) structures.

In total, 9 structures were found for the 12-vertex Cp_2_Co_2_E_2_B_8_H_8_ (E = S, Se) systems up to 9 kcal/mol (E = S) and 12 kcal/mol (E = Se) in energy (Figure 7 and Table 2). These structures are of three types. The polyhedra for the lowest-energy Cp_2_Co_2_S_2_B_8_H_8_ structure **B8Co2S2-1**, as well as those for 4 of the 5 next higher-energy structures **B8Co2S2-2**, **B8Co2S2-3**, **B8Co2S2-5**, and **B8Co2S2-6**, lying 3.0, 3.1, 3.8, and 5.6 kcal/mol in energy above **B8Co2S2-1**, respectively, can be derived from a Co_2_E_2_B_8_ icosahedron with an S–S edge by breaking the S–S edge and at least one S–B edge to create typically a gaping, bent hexagonal face. These five structures can be divided in two categories. In **B8Co2S2-1**, **B8Co2S2-5**, and **B8Co2S2-6,** the cobalt atoms are located in *meta* (non-adjacent, non-antipodal) positions of the original octahedron. However, in **B8Co2S2-2** and **B8Co2S2-4**, the cobalt atoms are located in *para* (antipodal) positions in the original icosahedron.

Substituting selenium for sulfur in the 12-vertex system to give Cp_2_Co_2_Se_2_B_8_H_8_ derivatives leads to a different energy ordering of the 9 lowest-energy structures (Table 2). The lowest-energy Cp_2_Co_2_Se_2_B_8_H_8_ structure **B8Co2Se2-1** and the higher-energy structure **B8Co2Se2-4**, which lies 4.0 kcal/mol higher in energy, are exceptional among the complete series of Cp_2_Co_2_E_2_B*_n_*_−4_H*_n_*_−4_ (*n* = 8 to 12) structures in exhibiting ideal *C*_2*v*_ symmetry, whereas all of the remaining structures of this type have the lower-symmetry point groups of *C*_1_, *C*_2_, or *C_s_*. These 2 structures are ni-12〈IV〉 structures derived from the 13-vertex *closo* deltahedron, namely, the docosahedron (Figure 1), by the removal of the unique degree-4 vertex, thereby creating a tetragonal face with alternating degree-5 and degree-4 vertices. In both **B8Co2Se2-1** and **B8Co2Se-4,** the tetragonal face has alternating cobalt and sulfur vertices. The Cp_2_Co_2_S_2_B_8_H_8_ structures corresponding to **B8Co2Se2-1** and **B8Co2Se2-4** are **B8Co2S-9** and **B8Co2S-8**, respectively, which lie 9.0 and 7.9 kcal/mol in energy above **B8Co2S-1**.

**Figure 8:**
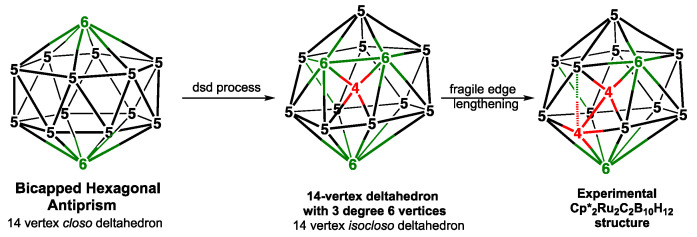
The *D*_6*d*_ bicapped hexagonal antiprism as the 14-vertex *closo* deltahedron and a 14-vertex *isocloso* deltahedron derived from it via a diamond-square-diamond process that is the polyhedron found in the experimentally known Cp*_2_Ru_2_C_2_B_10_H_12_. The 12-vertex *arachno* Cp_2_Co_2_E_2_B_8_H_8_ (E = S, Se) structures **B8Co2S2-3**, **B8Co2S2-7**, **B8Co2Se2-5**, and **B8Co2eS2-9** are obtained from this 14-vertex *isocloso* deltahedron by the removal of a degree-4 vertex and a degree-5 vertex, which are so situated that the resulting tetragonal and pentagonal faces share an edge.

The remaining 2 of the 9 lowest-energy Cp_2_Co_2_E_2_B_8_H_8_ (E = S, Se), structures, namely, **B8Co2S-3** and **B8Co2S-7** for E = S, which lie 3.1 and 7.1 kcal/mol, respectively, in energy above **B8Co2S-1**, and **B8Co2Se-5** and **B8Co2Se-9**, respectively, which lie 8.2 and 12.3 kcal/mol, respectively, in energy above **B8Co2Se2-1**, can be considered to be *arachno* ar-12〈V,V〉 structures that are obtained by removing 2 vertices from a 14-vertex deltahedron. However, the central 14-vertex deltahedron from which these structures are derived is not the 14-vertex *closo* deltahedron, namely, the *D*_6*d*_ bicapped hexagonal antiprism with 2 degree-6 vertices in antipodal positions, as well as 12 degree-5 vertices, but instead, it is a less symmetrical 14-vertex deltahedron with 3 degree-6 vertices, 10 degree-5 vertices, and 1 degree-4 vertex (Figure 8). This 14-vertex deltahedron is closely related to the 14-vertex polyhedron in experimentally known Cp*_2_Ru_2_C_2_B_10_H_12_ by lengthening an edge connecting a degree-6 vertex with a degree-5 vertex [41,42].

### 2.3. The Cp_2_Co_2_E_2_B_n−4_H_n−4_ (E = S, Se) Systems Having 8 to 10 Vertices

**Figure 9:**
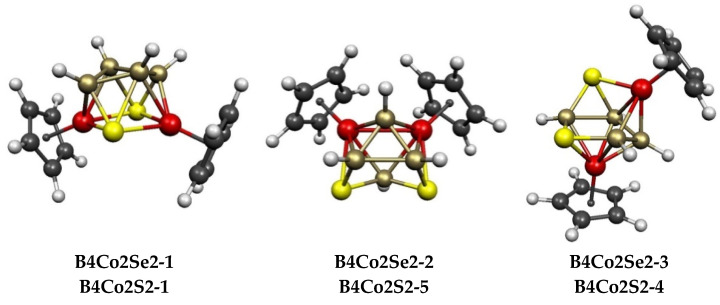

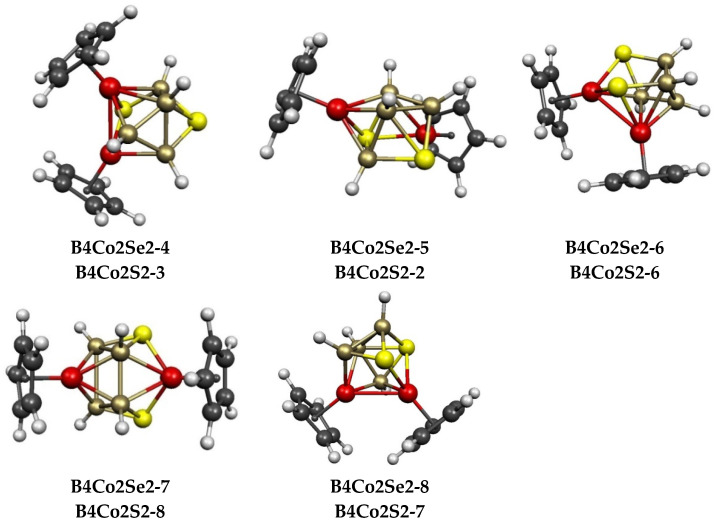
The eight lowest-energy Cp_2_Co_2_E_2_B_4_H_4_ (E = S, Se) structures.

The central Co_2_E_2_B_4_ polyhedra in 7 of the 8 lowest-energy structures of the 8-vertex Cp_2_Co_2_E_2_B_4_H_4_ (E = S, Se) systems (Figure 9 and Table 3) are all derived by the removal of a vertex from the *closo* 9-vertex deltahedron, namely, the tricapped trigonal prism (Figure 1). Removal of a degree-4 vertex from the tricapped, trigonal prism gives the bicapped trigonal prism, which is found in the lowest-energy structures **B4Co2E2-1** (E = S, Se), as well as the higher-energy structures **B4C2S2-2** and **B4Co2Se2-5**, which lie ~6 kcal/mol in energy above the lowest-energy structures (Table 3). In the lowest-energy structure **B4Co2E2-1**, the atoms of the open tetragonal face are alternating cobalt and chalcogen atoms with all four boron atoms located at interior vertices. The other bicapped trigonal prismatic Cp_2_Co_2_E_2_B_4_H_4_ (E = S, Se) structures have the two sulfur atoms as well as one of the cobalt atoms at the open tetragonal face, with the other cobalt atom at an interior vertex.

For the 8-vertex selenium derivative Cp_2_Co_2_Se_2_B_4_H_4,_ the 2 low-energy bicapped trigonal prismatic structures **B4Co2Se-1** and **B4Co2Se-2** are essentially isoenergetic, as they lie within ~1 kcal/mol of each other (Figure 9 and Table 3). The lowest-energy Cp_2_Co_2_Se_2_B_4_H_4_ ni-8〈V〉 structure **B4Co2Se2-3**, which is derived from a tricapped trigonal prism by removing a degree-5 rather than a degree-4 vertex, lies 11.1 kcal/mol above **B4Co2Se-1**. In total, 4 more ni-8〈V〉 Cp_2_Co_2_Se_2_B_4_H_4_ structures, namely, **B4Co2Se2-4**, **B4Co2Se2-5**, **B4Co2Se2-6**, and **B4Co2Se2-8,** lie in the energy range of 11 to 15 kcal/mol above **B4Co2Se2-1**. The potential energy surface of the corresponding 8-vertex sulfur system Cp_2_Co_2_Se_2_B_4_H_4_ is significantly different since 3 of the 5 ni-8〈V〉 structures **B4Co2S2-2**, **B4Co2S2-3**, and **B4Co2S2-4** lie within ~2 kcal/mol of the lowest-energy structure **B4Co2S2-1** and below the higher-energy, bicapped trigonal prism isomer **B4Co2S2-5**. In all eight lowest-energy Cp_2_Co_2_E_2_B_4_H_4_ (E = S, Se) structures, both sulfur atoms lie on tetragonal or pentagonal face vertices, which is consistent with the preference of sulfur for lower degree vertices in borane polyhedra.

Of the 8 lowest-energy Cp_2_Co_2_E_2_B_4_H_4_ (E = S, Se) structures, **B4Co2S2-8** and **B4Co2Se2-7**, which lie at 10.3 and 14.9 kcal/mol in energy, respectively, above the corresponding **B4Co2E2-1** structure, are not derived by removing a vertex from a tricapped trigonal prism (Figure 9 and Table 3). Instead, the central Co_2_S_2_B_4_ polyhedron in these structures is generated via the removal of the 2 degree-4 vertices that are bridged by the unique degree-6 vertex from the 10-vertex *isocloso* deltahedron of ideal *C*_3*v*_ symmetry. This leads to an *arachno* 8-vertex ar-8〈IV,IV〉 structure with 2 tetragonal faces sharing a cobalt atom and a sulfur atom. The process of removing 2 degree-4 vertices from the 10-vertex *isocloso* deltahedron to give the 8-vertex Cp_2_Co_2_E_2_B_4_H_4_ structures **B4Co2S2-8** and **B4Co2Se2-7** is analogous to the process of removing a degree-4 and a degree-5 vertex from a 14-vertex *isocloso* 14-vertex deltahedron (Figure 8) to give the 12-vertex ar-14〈IV,V〉 Cp_2_Co_2_E_2_B_8_H_8_ structures **B8Co2S23** and **B8Co2S27** (Figure 7) that are discussed above.

**Figure 10:**
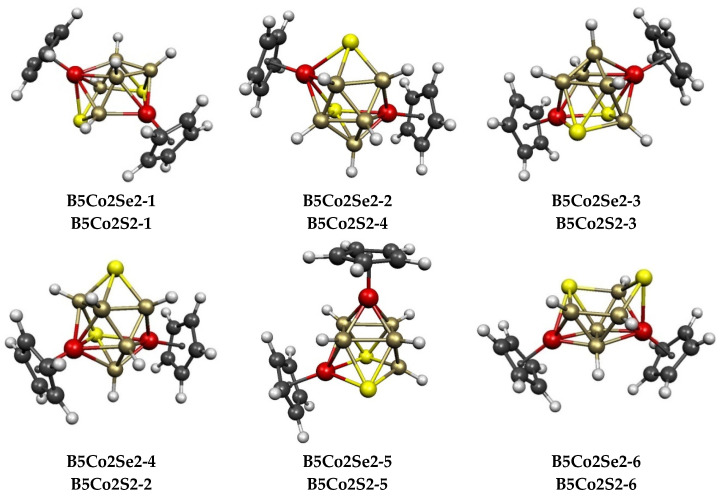
The six lowest-energy Cp_2_Co_2_E_2_B_5_H_5_ (E = S, Se) structures.

The central polyhedra of the 6 lowest-energy 9-vertex Cp_2_Co_2_E_2_B_5_H_5_ (E = S, Se) structures (Figure 10 and Table 4) are all derived from the 10-vertex *closo* deltahedron, namely, the bicapped square antiprism, by removing either a degree-4 vertex or a degree-5 vertex. Whether a degree-4 vertex is removed to give a capped square antiprism or a degree-5 vertex is removed to give a ni-9〈V〉 structure with a pentagonal face makes relatively little difference in energy since the 6 structures lying within 7 kcal/mol of the lowest-energy structures **B5Co2E2-1** (E = S, Se) include 2 representatives of the former type and 4 representatives of the latter type. Both chalcogen vertices are always located at a non-triangular face in all of the low-energy structures.

**Figure 11:**
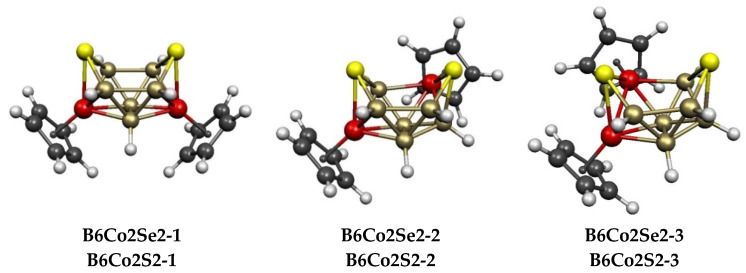
The three lowest-energy Cp_2_Co_2_E_2_B_6_H_6_ (E = S, Se) structures are oriented so that the bent hexagonal face is at the top.

The potential energy surfaces for the 10-vertex Cp_2_Co_2_E_2_B_6_H_6_ (E = S, Se) systems are the simplest of all, with only 3 structures lying within 16 kcal/mol (E = S) or 21 kcal/mol (E = Se) of the lowest-energy structures **B6Co2E2-1** (Figure 11 and Table 5). The central Co_2_E_2_B_6_ framework of all three structures is that of the very stable decaborane, B_10_H_14_, which is obtained via the removal of the unique degree-6 vertex of the *closo* 11-vertex deltahedron (sometimes called by the confusing name of “edge-coalesced icosahedron”) to create a bent hexagonal face. The structures **B6Co2E2-1**, in which the hexagonal face has only boron and sulfur atoms with the sulfur atoms in opposite (“*para*”) positions, is favored energetically over the next lowest-energy structures **B6Co2E2-1** by significant margins of ~11 kcal/mol (E = S) and ~12 kcal/mol (E = Se).

## 3. Theoretical Methods

The chemical models that were investigated in this study are based on various B*_n_*H*_n_*^2−^ polyhedra, for which a systematic substitution of 2 BH vertices with 2 CpCo units, followed by the substitution of 2 BH vertices with 2 chalcogen atoms (sulfur or selenium) led to the generation of a total of 5389 different starting structures for each of the Cp_2_Co_2_S_2_B*_n_*_−4_H*_n_*_−4_ and Cp_2_Co_2_Se_2_B*_n_*_−4_H*_n_*_−4_ systems *(n* = 8 to 12) (see the Supporting Information).

Full geometry optimizations were carried out on all systems by using the PBE0 DFT functional [43], coupled with the def2-TZVP basis set [44], as implemented in the Gaussian 09 package [45]. The natures of the stationary points after optimization were checked via calculations of the harmonic vibrational frequencies to ensure genuine minima. Furthermore, single-point energy calculations were performed on the lowest-energy optimized structures by using the DLPNO-CCSD(T) method [46,47,48,49,50,51,52,53,54,55,56,57,58,59] coupled with the def2-QZVP basis set, as implemented in the ORCA 3.0.3 software package [60,61,62,63,64,65,66,67,68]. Zero-point corrections taken from the PBE0/def2-TZVP computations were then added to the final energies.

The polyhedral dicobaltadithiaborane and dicobaltadiselenaborane structures Cp_2_Co_2_E_2_B*_n_*_4_H*_n_*_−4_ (E = S, Se; *n* = 8 to 12) are designated as **B(*n*−4)Co2E2-x** throughout the text, where ***n*** is the total number of polyhedral vertices, and **x** is the relative ordering of the structure on the energy scale. Only the lowest-energy and, thus, potentially chemically significant structures are considered in detail in this paper. More comprehensive structural information including higher-energy structures, connectivity information not readily seen in the figures, and orbital energies and HOMO-LUMO gaps are provided in the Supporting Information.

## 4. Summary

The central Co_2_E_2_B*_n_*_−4_ polyhedra in the low-energy structures of the *n*-vertex polyhedral dicobaltadithiaboranes and dicobaltadiselenaboranes Cp_2_Co_2_E_2_B*_n_*_−4_H*_n_*_−4_ (E = S, Se; *n* = 8 to 12) in general are generated from the (*n* + 1)-vertex most spherical *closo* deltahedra via the removal of a single vertex, leading to a tetragonal, pentagonal, or hexagonal face, depending on the degree of the vertex removed. In all of these low-energy structures, both chalcogen atoms are located on the non-triangular face vertices, reflecting the energetic preference of chalcogens for lower degree vertices. For the 8- and 9-vertex systems, the structures obtained via the removal of a degree-4 or degree-5 vertex from the corresponding (*n* + 1)-vertex *closo* deltahedra, namely, the tricapped trigonal prism and the bicapped square antiprism, have similar energies. The low-energy structures for the 10-vertex Cp_2_Co_2_E_2_B_6_H_6_ (E = S, Se) systems all have the framework of the most stable B*_n_*H*_n_*_+4_ borane, namely, B_10_H_14_ with a bent hexagonal face. The lowest-energy of these 10-vertex Cp_2_Co_2_E_2_B_6_H_6_ structures by significant margins exceeding 10 kcal/mol has only boron and both sulfur atoms located at the 6 hexagonal face vertices. The central polyhedra of all of the 11-vertex Cp_2_Co_2_E_2_B_7_H_7_ structures are similar to the polyhedron of the dicarbollide anion C_2_B_9_H_12_^−^ in being generated by loss of a vertex from a regular icosahedron to generate a pentagonal face.

In principle, the central polyhedra in most of the low-energy 12-vertex Cp_2_Co_2_E_2_B_8_H_8_ (E = S, Se) structures can be derived via the removal of a vertex from the 13-vertex *closo* deltahedron, namely, the docosahedron. However, the central polyhedron in most of the 12-vertex structures can also be derived from a Co_2_E_2_B_8_ icosahedron with adjacent chalcogen vertices by breaking the E–E edge and 1 or more E–B edges to create a hexagonal hole.

Two examples of *arachno* polyhedra were found among the set of lowest-energy Cp_2_Co_2_E_2_B*_n_*_−4_H*_n_*_−4_ (E = S, Se; *n* = 8 to 12) structures. The central polyhedron of one structure within 15 kcal/mol of the lowest-energy structure in each of the 8-vertex Cp_2_Co_2_E_2_B_4_H_4_ (E = S, Se) systems is an *arachno* polyhedron with 2 tetragonal faces sharing an edge that is derived from the 10-vertex *isocloso* deltahedron via the removal of the 2 degree-4 vertices bridged by the unique degree-6 vertex. In addition, 2 of the structures in each of the 12-vertex Cp_2_Co_2_E_2_B_8_H_8_ (E = S, Se) systems are ar12〈IV,V〉 structures that are derived from the 14-vertex *isocloso* deltahedron that is found in the experimentally known Cp*_2_Ru_2_C_2_B_10_H_12_ by removing the unique degree-4 vertex as well as a degree-5 vertex.

## Figures and Tables

**Table 1:** Relative energies (kcal/mol) and geometries of the lowest-energy 11-vertex Cp_2_Co_2_E_2_B_7_H_7_ (E = Se, S) structures. In all 8 structures, the central Co_2_E_2_B_7_ polyhedron is derived from an 11-vertex polyhedron that is obtained via the removal of a vertex from the 12-vertex regular icosahedron, leaving a pentagonal face.

Cp_2_Co_2_Se_2_B_7_H_7_		Cp_2_Co_2_S_2_B_7_H_7_		Vertex Degrees		Co^…^S	Co^…^Co (E = Se)		Pentagonal
Structure (sym)	∆E	Structure	∆E	S	Co	Edges	Dist (Å)	WBI	Face Atoms
**B7Co2Se2-1 (C_1_)**	0.0	**B7Co2S2-1**	0.0	4, 4	4, 4	3	3.91	0.10	SCoSBCo
**B7Co2Se2-2 (C_1_)**	2.5	**B7Co2S2-2**	1.8	4, 4	4, 5	3	3.81	0.10	SCoSBB
**B7Co2Se2-3 (C_1_)**	8.1	**B7Co2S2-3**	6.2	4, 4	4, 5	2	3.79	0.09	SCoBSB
**B7Co2Se2-4 (C_1_)**	9.4	**B7Co2S2-4**	7.7	4, 4	4, 5	2	3.71	0.10	SCoBSB
**B7Co2Se2-5 (C*_s_*)**	10.1	**B7Co2S2-5**	18.2	4, 4	4, 5	2	3.68	0.09	SCoSBB
**B7Co2Se2-6 (C*_s_*)**	11.8	**B7Co2S2-6**	19.9	4, 4	5, 5	2	3.73	0.09	SBSBB
**B7Co2Se2-7 (C_1_)**	13.5	**B7Co2S2-7**	10.7	4, 4	4, 5	1	3.67	0.08	SCoBSB
**B4Co2Se2-8 (C_1_)**	14.7	**B7Co2S2-8**	12.6	4, 4	5, 5	2	3.73	0.08	SBSBB

**Table 2:** Relative energies (kcal/mol) and geometries of the lowest-energy 12-vertex Cp_2_Co_2_E_2_B_8_H_8_ (E = S, Se) structures.

Cp_2_Co_2_Se_2_B_8_H_8_		Cp_2_Co_2_S_2_B_8_H_8_		Vertex Degrees		Co^…^S	Co^…^Co (E = Se)		
Structure (sym)	∆E	Structure	∆E	S	Co	Edges	Dist (Å)	WBI	Polyhedron
**B8Co2Se2-1 (*C*_2*v*_)**	0.0	**B8Co2S2-9**	9.0	5, 5	4, 4	4	3.18	0.17	ni-12〈IV〉
**B8Co2Se2-2 (*C*_1_)**	4.0	**B8Co2S2-1**	0.0	3, 3	5, 5	2	3.85	0.12	*meta* Co_2_ open icosahedron
**B8Co2Se2-3 (*C*_1_)**	4.5	**B8Co2S2-5**	3.8	3, 4	4, 5	2	3.83	0.09	*meta* Co_2_ open icosahedron
**B8Co2Se2-4 (*C*_2*v*_)**	5.3	**B8Co2S2-8**	7.9	4, 4	5, 5	4	3.48	0.15	ni-12〈IV〉
**B8Co2Se2-5 (*C*_1_)**	8.2	**B8Co2S2-3**	3.1	3, 4	5, 5	1	3.61	0.09	ar-12〈IV,V〉
**B8Co2Se2-6 (*C*_2_)**	8.2	**B8Co2S2-4**	3.5	3, 3	5, 5	2	4.22	0.05	*para* Co_2_ open icosahedron
**B8Co2Se2-7 (*C*_2_)**	9.3	**B8Co2S2-2**	3.0	3, 3	5, 5	2	4.82	0.08	*para* Co_2_ open icosahedron
**B8Co2Se2-8 (*C*_1_)**	11.8	**B8Co2S2-6**	5.6	3, 4	5, 5	1	3.73	0.11	*meta* Co_2_ open icosahedron
**B8Co2Se2-9 (*C*_1_)**	12.3	**B8Co2S2-7**	7.1	3, 4	5, 5	1	3.72	0.10	ar-12〈IV,V〉

**Table 3:** Relative energies (kcal/mol) and geometries of the lowest-energy Cp_2_Co_2_E_2_B_4_H_4_ (E = S, Se) structures.

Cp_2_Co_2_Se_2_B_4_H_4_		Cp_2_Co_2_S_2_B_4_H_4_		Vertex Degrees		Co^…^S	Co^…^Co (E = Se)		Non-Triang Face	
Structure (sym)	∆E	Structure	∆E	S	Co	Edges	Dist (Å)	WBI	Atoms	Polyhedron
**B4Co2Se2-1 (C_2_)**	0.0	**B4Co2S2-1**	0.0	4, 4	4, 4	4	3.39	0.12	SCoSCo	bicap trig prism
**B4Co2Se2-2 (C_s_)**	7.1	**B4Co2S2-5**	5.2	4, 4	4, 4	3	3.75	0.14	SCoSB	bicap trig prism
**B4Co2Se2-3 (C_1_)**	11.1	**B4Co2S2-4**	1.9	3, 3	4, 5	2	2.55	0.38	SCoBBB	ni-8〈V〉
**B4Co2Se2-4 (C_1_)**	11.4	**B4Co2S2-3**	0.7	3, 3	4, 5	2	3.44	0.10	SCoBSB	ni-8〈V〉
**B4Co2Se2-5 (C*_s_*)**	12.5	**B4Co2S2-2**	0.1	3, 3	5, 5	2	2.64	0.24	SCoBSB	ni-8〈V〉
**B4Co2Se2-6 (C_1_)**	13.5	**B4Co2S2-6**	6.5	3, 3	4, 5	3	2.59	0.40	SCoSBB	ni-8〈V〉
**B4Co2Se2-7 (C_1_)**	14.9	**B4Co2S2-8**	10.3	3, 4	4, 4	2	2.65	0.44	2 × SCoSB	ar-8〈IV,IV〉
**B4Co2Se2-8 (C*_s_*)**	15.3	**B4Co2S2-7**	8.3	3, 3	4, 4	2	2.62	0.09	SCoSBB	ni-8〈V〉

**Table 4:** Relative energies (kcal/mol) and geometries of the lowest-energy 9-vertex Cp_2_Co_2_E_2_B_5_H_5_ (E = S, Se) structures.

Cp_2_Co_2_Se_2_B_5_H_5_		Cp_2_Co_2_S_2_B_5_H_5_		Vertex Degrees		Co^…^S	Co^…^Co (E = Se)		Non-Triang Face	
Structure (sym)	∆E	Structure	∆E	S	Co	Edges	Dist(Å)	WBI	Atoms	Polyhedron
**B5Co2Se2-1 (*C*_1_)**	0.0	**B5Co2S2-1**	0.0	3, 4	4, 5	2	3.67	0.11	SCoBSB	ni-9〈V〉
**B5Co2Se2-2 (*C*_1_)**	0.7	**B5Co2S2-4**	3.9	3, 4	4, 5	3	3.82	0.07	SCoBSCo	ni-9〈V〉
**B5Co2Se2-3 (*C*_1_)**	0.9	**B5Co2S2-3**	3.6	4, 4	4, 5	3	3.77	0.12	SCoSB	capped square antiprism
**B5Co2Se2-4 (*C*_1_)**	2.7	**B5Co2S2-2**	2.8	3, 4	4, 5	3	3.40	0.07	SCoBSB	ni-9〈V〉
**B5Co2Se2-5 (*C*_s_)**	3.7	**B5Co2S2-5**	6.0	4, 4	4, 4	2	3.65	0.11	SCoSB	capped square antiprism
**B5Co2Se2-6 (*C*_1_)**	6.9	**B5Co2S2-6**	6.2	3, 4	4, 5	2	3.72	0.12	SBBSB	ni-9〈V〉

**Table 5:** Relative energies (kcal/mol) and geometries of the lowest-energy 10-vertex Cp_2_Co_2_E_2_B_6_H_6_ (E = S, Se) structures. In all 3 structures, the central Co_2_E_2_B_6_ polyhedron has the same geometry as the B_10_ polyhedron in decaborane-14 with a hexagonal face.

Cp_2_Co_2_Se_2_B_6_H_6_		Cp_2_Co_2_S_2_B_6_H_6_		Vertex Degrees		Co^…^S	Co^…^Co (E = Se)		Hexagonal Face	
Structure (sym)	∆E	Structure	∆E	S	Co	Edges	Dist (Å)	WBI	Atoms	Polyhedron
**B6Co2Se2-1 (*C*_2*v*_)**	0.0	**B6Co2S2-1**	0.0	3, 3	5, 5	2	3.77	0.10	SBBBBS	B_10_H_14_ framework
**B6Co2Se2-2 (*C*_1_)**	10.6	**B6Co2S2-2**	12.4	3, 3	4, 5	2	3.78	0.09	SBBSCoBS	B_10_H_14_ framework
**B6Co2Se2-3 (*C*_1_)**	15.7	**B6Co2S2-3**	18.1	3, 3	4, 5	2	2.49	0.41	SBBSCoBS	B_10_H_14_ framework

## Data Availability

The data presented in this study are available in supplementary material.

## Supplementary Materials

The following supporting information can be downloaded at: https://www.mdpi.com/article/10.3390/molecules28072988/s1, Initial structures, distance, and energy ranking tables, orbital energies and HOMO/LUMO gaps, complete Gaussian09 reference; concatenated .xyz file containing the Cartesian coordinates of the lowest-energy optimized structure. Table S1A: Initial 8-vertex starting structures. Table S1B: Distance matrices and energy rankings for the lowest energy Cp_2_Co_2_S_2_B_4_H_4_ structures. Table S1C: Distance matrices and energy rankings for the lowest energy Cp_2_Co_2_Se_2_gB_4_H_4_ structures. Table S2A: Initial 9-vertex starting structures. Table S2B: Distance matrices and energy rankings for the lowest energy Cp_2_Co_2_S_2_B_5_H_5_ structures. Table S2C: Distance matrices and energy rankings for the lowest energy Cp_2_Co_2_Se_5_B_5_H_5_ structures. Table S3A: Initial 10-vertex starting structures. Table S3B: Distance matrices and energy rankings for the lowest energy Cp_2_Co_2_S_2_B_6_H_6_ structures. Table S3C: Distance matrices and energy rankings for the lowest energy Cp_2_Co_2_Se_2_B_6_H_6_ structures. Table S4A: Initial 11-vertex starting structures. Table S4B: Distance matrices and energy rankings for the lowest energy Cp_2_Co_2_S_2_B_7_H_7_ structures. Table S4C: Distance matrices and energy rankings for the lowest energy Cp_2_Co_2_Se_2_B_7_H_7_ structures. Table S5A: Initial 12-vertex starting structures. Table S5B: Distance matrices and energy rankings for the lowest energy Cp_2_Co_2_S_2_B_8_H_8_ structures. Table S5C: Distance matrices and energy rankings for the lowest energy Cp_2_Co_2_Se_2_B_8_H_8_ structures. Table S6A: Distance matrices and energy rankings for the lowest energy permethylated Cp*_2_Co_2_S_2_B_7_H_7_ structures. Table S6B: Distance matrices and energy rankings for the lowest energy permethylated Cp*_2_Co_2_Se_2_B_7_H_7_ structures. Table S7: Orbital energies and HOMO-LUMO gaps for the lowest Cp_2_Co_2_S_2_B_n−4_H_n−4_ (*n* = 8 to 12) structures. Table S8: Orbital energies and HOMO-LUMO gaps for the lowest Cp_2_Co_2_Se_2_B_n−4_H_n−4_ (*n* = 8 to 12) structures. Table S9: Orbital energies and HOMO-LUMO gaps for the lowest permethylated Cp*_2_Co_2_E_2_B_7_H_7_ (E = S, Se) structures

## Sample Availability

Samples of the compounds are not available from the authors.
