# Supplementary material for: Polyhedral Dicobaltadithiaboranes and Dicobaltdiselenaboranes as Examples of Bimetallic Nido Structures without Bridging Hydrogens
Source: Molecules. 2023 Mar 27;28(7):2988. doi: 10.3390/molecules28072988 (PMC10095674; doi:10.3390/molecules28072988)
Supplement: Supplementary file 1 [file molecules-28-02988-s001.zip › Cp2Co2E2BnHn (E=S,Se) suppinfo 2-23.pdf]

Polyhedral Dicobaltadithiaboranes and Dicobaltdiselenaboranes as Examples of Bimetallic *Nido* Structures without Bridging Hydrogens

Amr A. A. Attia,[1] Alexandru Lupan,*[1] and R. Bruce King*[2]

[1] *Faculty of Chemistry and Chemical Engineering, Babeş-Bolyai University, Cluj-Napoca, Romania*
[2] *Department of Chemistry, University of Georgia, Athens, Georgia, 30602, USA*

Supporting Information

Complete Gaussian09 Reference.
Table S1A: Initial 8-vertex starting structures.
Table S1B: Distance matrices and energy rankings for the lowest energy $Cp_2Co_2S_2B_4H_4$ structures.
Table S1C: Distance matrices and energy rankings for the lowest energy $Cp_2Co_2Se_2B_4H_4$ structures.
Table S2A: Initial 9-vertex starting structures.
Table S2B: Distance matrices and energy rankings for the lowest energy $Cp_2Co_2S_2B_5H_5$ structures.
Table S2C: Distance matrices and energy rankings for the lowest energy $Cp_2Co_2Se_2B_5H_5$ structures.
Table S3A: Initial 10-vertex starting structures.
Table S3B: Distance matrices and energy rankings for the lowest energy $Cp_2Co_2S_2B_6H_6$ structures.
Table S3C: Distance matrices and energy rankings for the lowest energy $Cp_2Co_2Se_2B_6H_6$ structures.
Table S4A: Initial 11-vertex starting structures.
Table S4B: Distance matrices and energy rankings for the lowest energy $Cp_2Co_2S_2B_7H_7$ structures.
Table S4C: Distance matrices and energy rankings for the lowest energy $Cp_2Co_2Se_2B_7H_7$ structures.
Table S5A: Initial 12-vertex starting structures.
Table S5B: Distance matrices and energy rankings for the lowest energy $Cp_2Co_2S_2B_8H_8$ structures.
Table S5C: Distance matrices and energy rankings for the lowest energy $Cp_2Co_2Se_2B_8H_8$ structures.
Table S6A: Distance matrices and energy rankings for the lowest energy permethylated $Cp^*{}_2Co_2S_2B_7H_7$ structures.
Table S6B: Distance matrices and energy rankings for the lowest energy permethylated $Cp^*{}_2Co_2Se_2B_7H_7$ structures.
Table S7: Orbital energies and HOMO-LUMO gaps for the lowest $Cp_2Co_2S_2B_{n-4}H_{n-4}$ (n = 8 to 12) structures.
Table S8: Orbital energies and HOMO-LUMO gaps for the lowest $Cp_2Co_2Se_2B_{n-4}H_{n-4}$ (n = 8 to 12) structures.
Table S9: Orbital energies and HOMO-LUMO gaps for the lowest permethylated $Cp^*{}_2Co_2E_2B_7H_7$ (E = S, Se) structures.

Complete Gaussian09 Reference.

Gaussian 09, Revision E.01, M. J. Frisch, G. W. Trucks, H. B. Schlegel, G. E. Scuseria, M. A. Robb, J. R. Cheeseman, G. Scalmani, V. Barone, B. Mennucci, G. A. Petersson, H. Nakatsuji, M. Caricato, X. Li, H. P. Hratchian, A. F. Izmaylov, J. Bloino, G. Zheng, J. L. Sonnenberg, M. Hada, M. Ehara, K. Toyota, R. Fukuda, J. Hasegawa, M. Ishida, T. Nakajima, Y. Honda, O. Kitao, H. Nakai, T. Vreven, J. A. Montgomery, Jr., J. E. Peralta, F. Ogliaro, M. Bearpark, J. J. Heyd, E. Brothers, K. N. Kudin, V. N. Staroverov, R. Kobayashi, J. Normand, K. Raghavachari, A. Rendell, J. C. Burant, S. S. Iyengar, J. Tomasi, M. Cossi, N. Rega, J. M. Millam, M. Klene, J. E. Knox, J. B. Cross, V. Bakken, C. Adamo, J. Jaramillo, R. Gomperts, R. E. Stratmann, O. Yazyev, A. J. Austin, R. Cammi, C. Pomelli, J. W. Ochterski, R. L. Martin, K. Morokuma, V. G. Zakrzewski, G. A. Voth, P. Salvador, J. J. Dannenberg, S. Dapprich, A. D. Daniels, O. Farkas, J. B. Foresman, J. V. Ortiz, J. Cioslowski, and D. J. Fox, Gaussian, Inc., Wallingford CT, 2016.

Table S1A: Initial 8-vertex $[BH]_8{}^{2-}$ polyhedra upon which the starting structures are based; the H atoms are omitted for clarity.

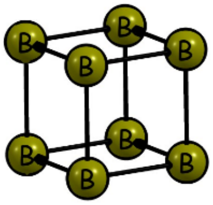 1. Cube	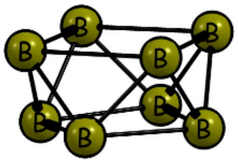 2. Antiprism	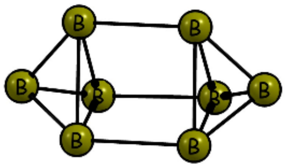 3. Trigonal Prism
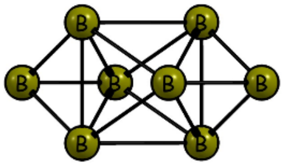 4. Trigonal Antiprism	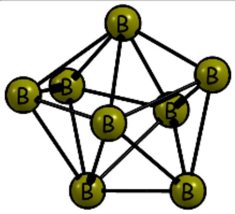 5. Bisdisphenoid	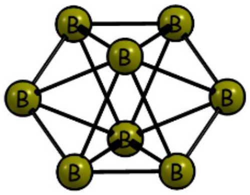 6. Hexagonal bipyramid
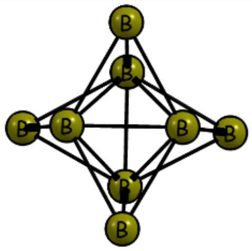 7. All-capped tetrahedron	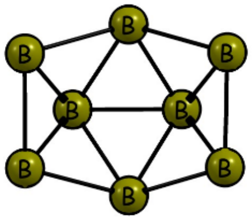 8. Nido structure	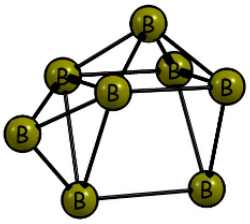 9. Dicapped trigonal prism (C_s)
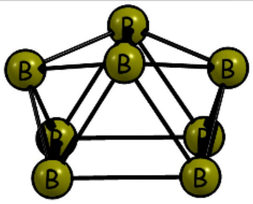 10. Dicapped trigonal prism (C_{2v})	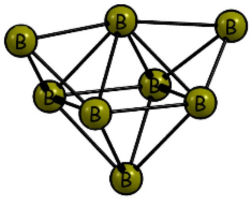 11. Dicapped octahedron	

Table S1B: Distance table for the lowest-lying $Cp_2Co_2S_2B_4H_4$ optimized structures obtained at the PBE0/def2-TZVP level of theory. Included are the zero-point corrected absolute energy in (a.u.) at the DLPNO-CCSD(T)/def2-QZVP level of theory with zero-point energy obtained from the PBE0/def2-TZVP computations, relative energy in (kcal/mol), symmetry and Wiberg bond indecies. For clarity, only the atoms forming the cluster framework are shown.

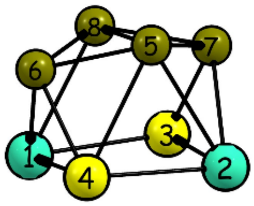

1. -4049.627167 0.0 C_2

WBI: Co1-Co2: 0.1138

		1	2	3	4	5
1	Co	0.000000				
2	Co	3.220370	0.000000			
3	S	2.235578	2.237026	0.000000		
4	S	2.237551	2.235209	3.098611	0.000000	
5	B	3.048692	2.210637	2.858098	2.223067	0.000000
6	B	2.053738	3.232586	3.210043	1.848254	1.786828
7	B	3.231789	2.054528	1.847583	3.209811	1.690494
8	B	2.211298	3.049244	2.224988	2.857964	1.718218

		6	7	8
6	B	0.000000		
7	B	2.947801	0.000000	
8	B	1.689430	1.786381	0.000000

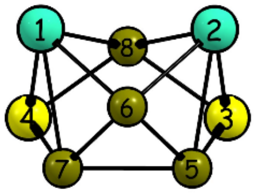

2. -4049.626963 +0.1 C_s

WBI: Co1-Co2: 0.2970

		1	2	3	4	5
1	Co	0.000000				
2	Co	2.640002	0.000000			
3	S	3.564705	2.181303	0.000000		
4	S	2.182312	3.575664	3.024990	0.000000	
5	B	3.126770	2.144828	1.838857	3.055198	0.000000
6	B	2.000369	2.000265	3.039815	3.043506	1.712793
7	B	2.143866	3.130669	3.053896	1.839533	1.966164
8	B	2.273836	2.285557	1.869124	1.867222	2.715907

		6	7	8
6	B	0.000000		
7	B	1.713366	0.000000	
8	B	2.918687	2.713411	0.000000

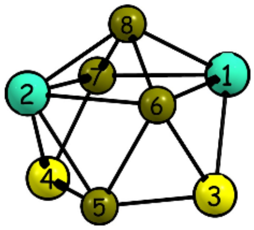

3. -4049.625996 +0.7 C_1

WBI: Co1-Co2: 0.1191

		1	2	3	4	5
1	Co	0.000000				
2	Co	3.410811	0.000000			
3	S	2.194368	3.416179	0.000000		
4	S	3.378562	2.203731	3.129701	0.000000	
5	B	3.118292	2.148034	1.857196	1.856739	0.000000
6	B	2.168685	2.186000	1.876642	3.167560	2.017013
7	B	2.220387	2.197787	3.217030	1.836265	2.743294
8	B	1.930810	1.993143	3.094614	3.063830	2.972706

		6	7	8
6	B	0.000000		
7	B	2.749425	0.000000	
8	B	1.755993	1.737701	0.000000

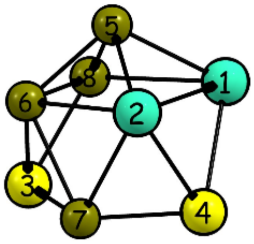

4. -4049.624139 +1.9 C_1

WBI: Co1-Co2: 0.4081

		1	2	3	4	5
1	Co	0.000000				
2	Co	2.513388	0.000000			
3	S	3.415941	3.459805	0.000000		
4	S	2.152907	2.171505	3.107366	0.000000	
5	B	1.991405	2.006988	3.034064	3.121391	0.000000
6	B	3.072877	2.172756	1.924844	3.129773	1.719730
7	B	3.104757	2.174735	1.877856	1.840869	2.945758
8	B	2.198097	3.068214	1.864674	3.142236	1.689039
		6	7	8		
6	B	0.000000				
7	B	1.943052	0.000000			
8	B	1.851173	2.731794	0.000000		

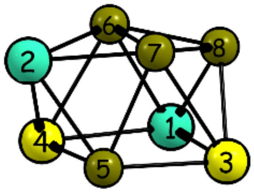

5. -4049.618924 +5.2 C_1

WBI: Co1-Co2: 0.1353

		1	2	3	4	5
1	Co	0.000000				
2	Co	3.713452	0.000000			
3	S	2.269266	3.502475	0.000000		
4	S	2.251071	2.181820	3.032010	0.000000	
5	B	2.889006	2.019865	1.984002	1.898975	0.000000
6	B	2.250035	2.050279	2.868388	2.093038	2.580929
7	B	3.089765	1.946915	2.169458	2.868803	1.963935
8	B	1.995737	3.279041	1.883707	3.115593	2.865680
		6	7	8		
6	B	0.000000				
7	B	1.764317	0.000000			
8	B	1.728118	1.783341	0.000000		

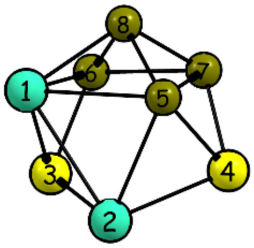

6. -4049.616830 +6.5 C_1

WBI: Co1-Co2: 0.3734

		1	2	3	4	5
1	Co	0.000000				
2	Co	2.563679	0.000000			
3	S	2.184768	2.183756	0.000000		
4	S	3.614742	2.192616	3.183153	0.000000	
5	B	2.335413	2.258653	3.171576	1.912641	0.000000
6	B	2.162024	3.177954	1.822107	3.134512	2.750721
7	B	3.141043	3.172459	3.109599	1.831192	1.860274
8	B	2.001333	3.314180	3.011183	2.978283	1.726986
		6	7	8		
6	B	0.000000				
7	B	2.013953	0.000000			
8	B	1.707134	1.653379	0.000000		

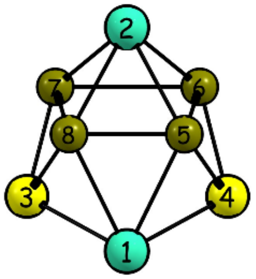

7. -4049.614005 +8.3 C_s

WBI: Co1-Co2: 0.0899

		1	2	3	4	5
1	Co	0.000000				
2	Co	3.588300	0.000000			
3	S	2.208061	3.302103	0.000000		
4	S	2.208692	3.302305	3.213666	0.000000	
5	B	2.108063	2.016316	3.125775	1.967338	0.000000
6	B	3.206068	1.909290	3.281199	1.821320	1.906645
7	B	3.205411	1.910012	1.821784	3.277189	2.808560
8	B	2.111200	2.015312	1.965873	3.128700	1.839670

		6	7	8
6	B	0.000000		
7	B	2.313584	0.000000	
8	B	2.810101	1.907077	0.000000

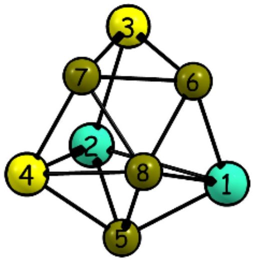

8. -4049.610774 +10.3 C_1

WBI: Co1-Co2: 0.4023

		1	2	3	4	5
1	Co	0.000000				
2	Co	2.614705	0.000000			
3	S	2.987412	2.261676	0.000000		
4	S	3.264344	2.255712	3.006674	0.000000	
5	B	1.894031	2.078376	3.406565	1.918069	0.000000
6	B	2.007256	3.035889	1.873763	3.073282	2.923849
7	B	3.164579	2.843302	1.895191	1.920002	2.944335
8	B	2.044177	3.009245	2.951557	2.075170	1.861945

		6	7	8
6	B	0.000000		
7	B	1.800714	0.000000	
8	B	1.726839	1.835845	0.000000

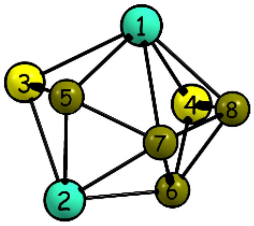

9. -4049.607227 +12.5 C_1

WBI: Co1-Co2: 0.0832

		1	2	3	4	5
1	Co	0.000000				
2	Co	3.305704	0.000000			
3	S	2.259876	2.259420	0.000000		
4	S	2.259191	3.386480	3.167898	0.000000	
5	B	2.204932	1.990814	1.793410	3.606819	0.000000
6	B	2.912757	2.022545	3.153818	1.937833	2.960114
7	B	2.334790	2.061666	2.946480	2.855767	1.805401
8	B	2.127846	3.301021	3.631984	1.818631	3.110078

		6	7	8
6	B	0.000000		
7	B	1.746401	0.000000	
8	B	1.804287	1.722319	0.000000

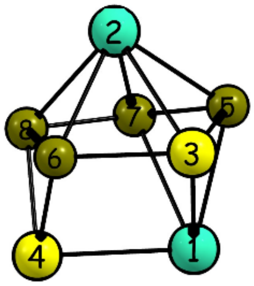

10. -4049.603115 +15.1 C_1

WBI: Co1-Co2: 0.1116

		1	2	3	4	5
1	Co	0.000000				
2	Co	3.359728	0.000000			
3	S	2.288391	2.274002	0.000000		
4	S	2.236882	3.366887	2.998125	0.000000	
5	B	2.066648	2.049664	1.954936	3.421311	0.000000
6	B	2.826081	2.102613	1.937452	1.860638	2.947805
7	B	2.264746	2.136500	2.951623	2.741192	1.667438
8	B	2.911989	2.142867	3.166528	1.908889	2.870287

		6	7	8
6	B	0.000000		
7	B	2.828071	0.000000	
8	B	1.951344	1.667825	0.000000

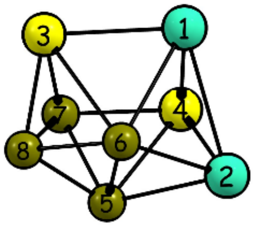

11. -4049.600967 +16.4 C_1

WBI: Co1-Co2: 0.4385

		1	2	3	4	5
1	Co	0.000000				
2	Co	2.441559	0.000000			
3	S	2.236330	3.668368	0.000000		
4	S	2.214963	2.160534	2.980688	0.000000	
5	B	3.086135	2.016274	2.878884	2.265268	0.000000
6	B	2.248482	2.001757	2.333073	2.894224	1.751822
7	B	2.874446	3.205961	1.937138	1.926509	1.894558
8	B	3.204679	3.253107	1.863952	3.134305	1.652716

		6	7	8
6	B	0.000000		
7	B	2.691097	0.000000	
8	B	1.797902	1.768749	0.000000

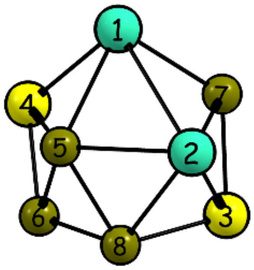

12. -4049.597840 +18.4 C_1

WBI: Co1-Co2: 0.3590

		1	2	3	4	5
1	Co	0.000000				
2	Co	2.518526	0.000000			
3	S	3.321915	2.220922	0.000000		
4	S	2.208373	3.658385	3.501102	0.000000	
5	B	2.064485	2.099700	2.981039	2.064986	0.000000
6	B	3.203047	3.320485	2.840085	1.752150	1.853275
7	B	1.937590	2.106423	1.837983	3.027686	2.815288
8	B	3.272548	2.055310	1.880525	2.978731	1.776146

		6	7	8
6	B	0.000000		
7	B	3.340174	0.000000	
8	B	1.728489	2.864511	0.000000

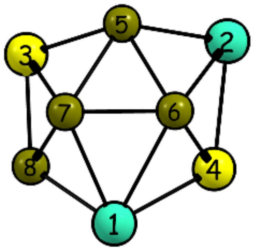

13. -4049.596917 +19.0 C_1

WBI: Co1-Co2: 0.1473

		1	2	3	4	5
1	Co	0.000000				
2	Co	3.680764	0.000000			
3	S	3.295857	3.247351	0.000000		
4	S	2.184893	2.075512	3.546497	0.000000	
5	B	3.226053	2.036653	1.912546	2.955348	0.000000
6	B	2.100658	2.185551	3.016849	1.964517	1.739085
7	B	2.023833	3.343982	2.018259	3.069723	1.813589
8	B	1.936704	3.761238	1.813554	2.955841	2.864763
		6	7	8		
6	B	0.000000				
7	B	1.763554	0.000000			
8	B	2.879596	1.853079	0.000000		

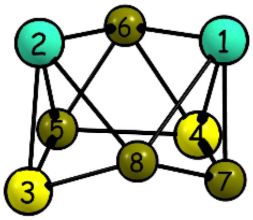

14. -4049.591436 +22.4 C_1

WBI: Co1-Co2: 0.2850

		1	2	3	4	5
1	Co	0.000000				
2	Co	2.707845	0.000000			
3	S	3.610077	2.172572	0.000000		
4	S	2.246583	3.317796	3.194318	0.000000	
5	B	3.112022	2.094462	1.834774	2.086004	0.000000
6	B	1.952845	1.983415	3.130965	1.971397	1.813889
7	B	2.037021	3.383038	2.887912	1.897922	2.946851
8	B	2.387840	2.370688	1.875676	2.892143	2.736934
		6	7	8		
6	B	0.000000				
7	B	2.986973	0.000000			
8	B	3.031333	1.648193	0.000000		

Table S1C: Distance table for the lowest-lying $Cp_2Co_2Se_2B_4H_4$ optimized structures obtained at the PBE0/def2-TZVP level of theory. Included are the zero-point corrected absolute energy in (a.u.) at the DLPNO-CCSD(T)/def2-QZVP level of theory with zero-point energy obtained from the PBE0/def2-TZVP computations, relative energy in (kcal/mol), symmetry and Wiberg bond indecies. For clarity, only the atoms forming the cluster framework are shown.

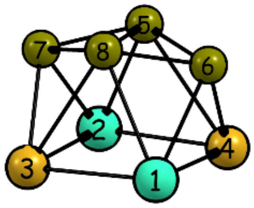

1. -8056.041300 0.0 C_2

WBI: Co1-Co2: 0.1158

		1	2	3	4	5
1	Co	0.000000				
2	Co	3.391772	0.000000			
3	Se	2.359782	2.361989	0.000000		
4	Se	2.363950	2.361966	3.286198	0.000000	
5	B	3.095339	2.228107	3.003620	2.363068	0.000000
6	B	2.064674	3.307056	3.350242	1.990617	1.792841
7	B	3.305211	2.064218	1.990249	3.350433	1.687272
8	B	2.227354	3.095715	2.364051	3.003662	1.716199
		6	7	8		
6	B	0.000000				
7	B	2.959389	0.000000			
8	B	1.686552	1.792570	0.000000		

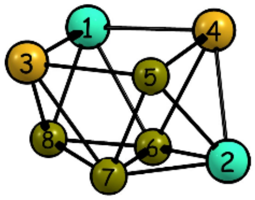

2. -8056.029989 +7.1 C_1

WBI: Co1-Co2: 0.1398

		1	2	3	4	5
1	Co	0.000000				
2	Co	3.748092	0.000000			
3	Se	2.394735	3.642449	0.000000		
4	Se	2.367475	2.301183	3.225294	0.000000	
5	B	3.068154	2.015079	2.133172	2.050563	0.000000
6	B	2.258819	2.050716	3.006204	2.285366	2.654530
7	B	3.115052	1.957270	2.284367	3.021031	1.957872
8	B	2.007600	3.291845	2.023331	3.284184	2.964607
		6	7	8		
6	B	0.000000				
7	B	1.758711	0.000000			
8	B	1.713190	1.795415	0.000000		

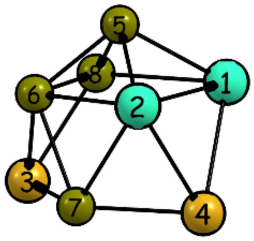

3. -8056.023622 +11.1 C_1

WBI: Co1-Co2: 0.3788

		1	2	3	4	5
1	Co	0.000000				
2	Co	2.553563	0.000000			
3	Se	3.583847	3.631334	0.000000		
4	Se	2.279810	2.299297	3.316525	0.000000	
5	B	1.988370	2.006878	3.191549	3.249452	0.000000
6	B	3.090671	2.154126	2.075784	3.244085	1.722744
7	B	3.212195	2.160097	2.039804	1.995147	2.958017
8	B	2.225025	3.092677	2.001383	3.292752	1.683562
		6	7	8		
6	B	0.000000				
7	B	1.902761	0.000000			
8	B	1.866873	2.813085	0.000000		

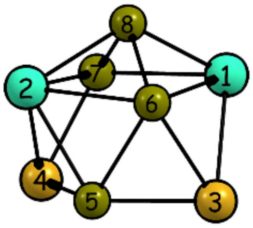

4. -8056.023138 +11.4 C_1

WBI: Co1-Co2: 0.0998

		1	2	3	4	5
1	Co	0.000000				
2	Co	3.444586	0.000000			
3	Se	2.321168	3.589612	0.000000		
4	Se	3.529226	2.326581	3.342630	0.000000	
5	B	3.206707	2.126747	2.011188	2.016496	0.000000
6	B	2.202163	2.165969	2.033647	3.282798	1.970219
7	B	2.250418	2.219533	3.397851	1.974410	2.842465
8	B	1.930539	2.002108	3.249760	3.198525	2.980748
		6	7	8		
6	B	0.000000				
7	B	2.758522	0.000000			
8	B	1.750610	1.727184	0.000000		

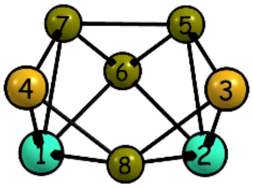

5. -8056.021317 +12.5 C_s

WBI: Co1-Co2: 0.2379

		1	2	3	4	5
1	Co	0.000000				
2	Co	2.642715	0.000000			
3	Se	3.714077	2.303985	0.000000		
4	Se	2.305410	3.721959	3.220949	0.000000	
5	B	3.146851	2.165845	1.978629	3.207066	0.000000
6	B	2.000523	2.000637	3.181695	3.184727	1.706430
7	B	2.165011	3.149817	3.206348	1.979776	1.976352
8	B	2.246844	2.256063	2.031894	2.029368	2.809838
		6	7	8		
6	B	0.000000				
7	B	1.706819	0.000000			
8	B	2.927168	2.807750	0.000000		

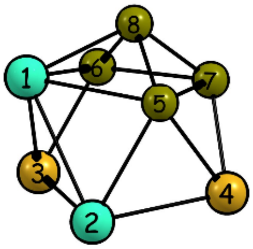

6. -8056.019762 +13.5 C_1

WBI: Co1-Co2: 0.4048

		1	2	3	4	5
1	Co	0.000000				
2	Co	2.589334	0.000000			
3	Se	2.311576	2.312841	0.000000		
4	Se	3.777160	2.325025	3.367782	0.000000	
5	B	2.296562	2.249592	3.273785	2.084220	0.000000
6	B	2.185495	3.288546	1.964941	3.292967	2.754704
7	B	3.159566	3.261582	3.254483	1.964593	1.886298
8	B	2.001943	3.358350	3.145842	3.134372	1.723223
		6	7	8		
6	B	0.000000				
7	B	2.036333	0.000000			
8	B	1.695814	1.647498	0.000000		

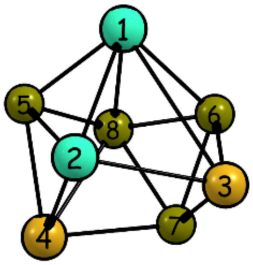

7. -8056.017516 +14.9 C_1

WBI: Co1-Co2: 0.4374

		1	2	3	4	5
1	Co	0.000000				
2	Co	2.651394	0.000000			
3	Se	3.033770	2.390746	0.000000		
4	Se	3.414669	2.377502	3.207340	0.000000	
5	B	1.896264	2.066188	3.502803	2.068562	0.000000
6	B	2.005504	3.171143	2.017190	3.217272	2.953328
7	B	3.179075	3.006886	2.051526	2.056116	3.024669
8	B	2.042843	3.071520	3.083096	2.199482	1.884849

		6	7	8
6	B	0.000000		
7	B	1.789745	0.000000	
8	B	1.720710	1.851323	0.000000

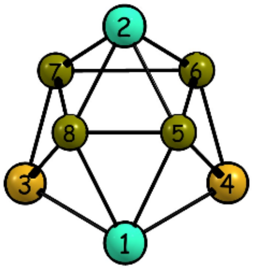

8. -8056.016885 +15.3 C_s

WBI: Co1-Co2: 0.0936

		1	2	3	4	5
1	Co	0.000000				
2	Co	3.623997	0.000000			
3	Se	2.336778	3.462867	0.000000		
4	Se	2.337378	3.461956	3.395074	0.000000	
5	B	2.117345	2.012345	3.266062	2.127232	0.000000
6	B	3.291167	1.906200	3.448707	1.962841	1.917875
7	B	3.290263	1.907259	1.963440	3.444444	2.820549
8	B	2.121472	2.012049	2.124995	3.269599	1.813915

		6	7	8
6	B	0.000000		
7	B	2.363713	0.000000	
8	B	2.822964	1.916050	0.000000

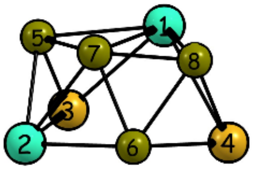

9. -8056.015113 +16.4 C_1

WBI: Co1-Co2: 0.0906

		1	2	3	4	5
1	Co	0.000000				
2	Co	3.348626	0.000000			
3	Se	2.392772	2.392321	0.000000		
4	Se	2.394196	3.512834	3.281079	0.000000	
5	B	2.211450	1.995485	1.944286	3.736177	0.000000
6	B	2.964132	2.027473	3.292256	2.099642	2.968532
7	B	2.289707	2.064993	3.070905	2.991595	1.783288
8	B	2.131420	3.311487	3.758143	1.961344	3.121644

		6	7	8
6	B	0.000000		
7	B	1.741432	0.000000	
8	B	1.808087	1.715788	0.000000

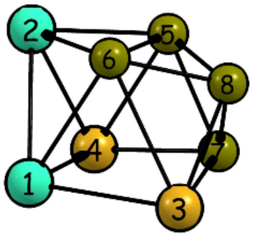

10. -8056.013310 +17.5 C_1

WBI: Co1-Co2: 0.4671

		1	2	3	4	5
1	Co	0.000000				
2	Co	2.462502	0.000000			
3	Se	2.360969	3.815992	0.000000		
4	Se	2.343085	2.285970	3.187484	0.000000	
5	B	3.127436	2.023256	3.019916	2.358914	0.000000
6	B	2.269097	2.008495	2.432351	3.017016	1.750990
7	B	3.045212	3.303583	2.091548	2.077459	1.903952
8	B	3.292695	3.277117	2.003397	3.259653	1.655639

		6	7	8
6	B	0.000000		
7	B	2.727870	0.000000	
8	B	1.804844	1.757580	0.000000

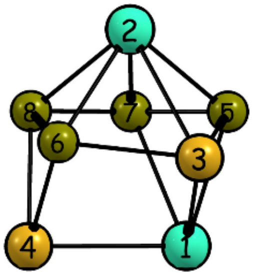

11. -8056.011567 +18.6 C_1

WBI: Co1-Co2: 0.0969

		1	2	3	4	5
1	Co	0.000000				
2	Co	3.409820	0.000000			
3	Se	2.403137	2.385164	0.000000		
4	Se	2.366438	3.528879	3.185760	0.000000	
5	B	2.060726	2.069092	2.107603	3.550257	0.000000
6	B	2.992912	2.116948	2.092666	2.016187	3.043636
7	B	2.279736	2.134131	3.077958	2.818442	1.666798
8	B	3.015255	2.121266	3.278417	2.055864	2.888150

		6	7	8
6	B	0.000000		
7	B	2.829963	0.000000	
8	B	1.896794	1.667272	0.000000

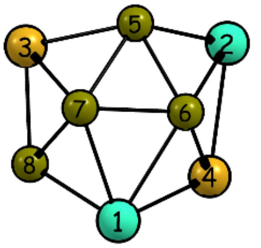

12. -8056.005557 +22.4 C_1

WBI: Co1-Co2: 0.1236

		1	2	3	4	5
1	Co	0.000000				
2	Co	3.763412	0.000000			
3	Se	3.450286	3.334967	0.000000		
4	Se	2.315185	2.210279	3.729856	0.000000	
5	B	3.260882	2.039816	2.058168	3.114059	0.000000
6	B	2.107579	2.203774	3.148720	2.128650	1.728090
7	B	2.019069	3.360446	2.161870	3.205771	1.819072
8	B	1.933870	3.860860	1.961100	3.044049	2.955000

		6	7	8
6	B	0.000000		
7	B	1.756442	0.000000	
8	B	2.898572	1.864743	0.000000

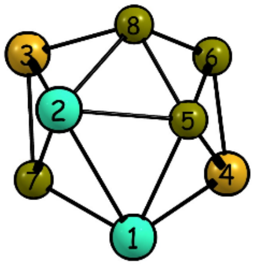

13. -8056.000986 +25.2 C_1

WBI: Co1-Co2: 0.3663

		1	2	3	4	5
1	Co	0.000000				
2	Co	2.523338	0.000000			
3	Se	3.464797	2.345629	0.000000		
4	Se	2.341649	3.811242	3.655111	0.000000	
5	B	2.064227	2.104390	3.095905	2.230071	0.000000
6	B	3.270610	3.323174	2.874645	1.893487	1.878061
7	B	1.927345	2.113863	1.995960	3.125238	2.826746
8	B	3.311347	2.053903	2.026671	3.140592	1.761490

		6	7	8
6	B	0.000000		
7	B	3.404988	0.000000	
8	B	1.720758	2.967525	0.000000

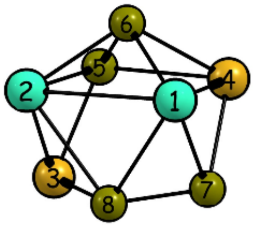

14. -8055.995373 +28.7 C_1

WBI: Co1-Co2: 0.2431

		1	2	3	4	5
1	Co	0.000000				
2	Co	2.729173	0.000000			
3	Se	3.771528	2.297158	0.000000		
4	Se	2.361632	3.476285	3.402231	0.000000	
5	B	3.174466	2.098516	1.976574	2.252325	0.000000
6	B	1.968211	1.995885	3.267623	2.106059	1.803995
7	B	2.045864	3.399854	3.008878	2.035129	3.101804
8	B	2.334691	2.309885	2.040867	3.027977	2.838025

		6	7	8
6	B	0.000000		
7	B	3.042453	0.000000	
8	B	2.996959	1.651631	0.000000

Table S2A: Initial 9-vertex $[BH]_9^{2-}$ polyhedra upon which the starting structures are based; the H atoms are omitted for clarity.

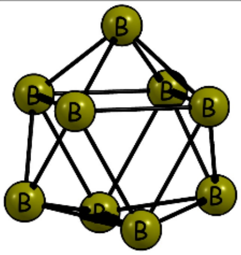 1. Tricapped trigonal prism	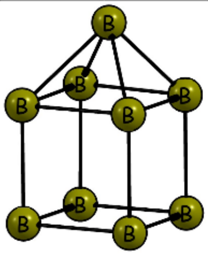 2. Capped cube	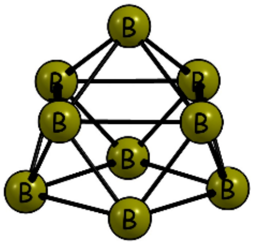 3. Capped square antiprism
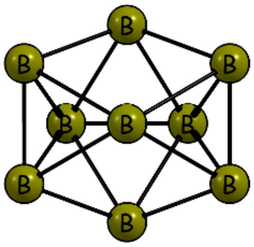 4. Isocloso	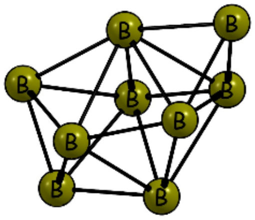 5. Capped bisdisphenoid	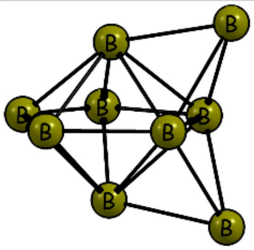 6. Bicapped pentagonal bipyramid
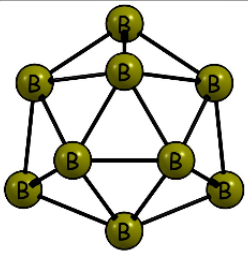 7. 10vx-Isocloso-1vx		

Table S2B: Distance table for the lowest-lying $Cp_2Co_2S_2B_5H_5$ optimized structures obtained at the PBE0/def2-TZVP level of theory. Included are the zero-point corrected absolute energy in (a.u.) at the DLPNO-CCSD(T)/def2-QZVP level of theory with zero-point energy obtained from the PBE0/def2-TZVP computations, relative energy in (kcal/mol), symmetry and Wiberg bond indecies. For clarity, only the atoms forming the cluster framework are shown.

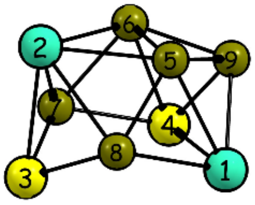

1. -4075.062142 0.0 C_1

WBI: Co1-Co2: 0.0919

		1	2	3	4	5
1	Co	0.000000				
2	Co	3.652380	0.000000			
3	S	3.260907	2.190754	0.000000		
4	S	2.280199	3.453907	3.155400	0.000000	
5	B	2.108376	2.134235	3.106050	2.819848	0.000000
6	B	2.991651	2.045309	3.156983	2.063890	1.798930
7	B	3.259811	2.112415	1.850252	1.958271	2.867781
8	B	2.082725	2.178162	1.864464	3.144835	1.768622
9	B	1.950700	3.350741	3.936292	1.915917	1.718440
		6	7	8	9	
6	B	0.000000				
7	B	1.881296	0.000000			
8	B	2.860910	2.714144	0.000000		
9	B	1.766168	3.039351	2.990969	0.000000	

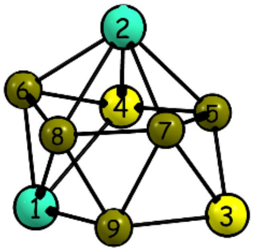

2. -4075.057638 +2.8 C_1

WBI: Co1-Co2: 0.0868

		1	2	3	4	5
1	Co	0.000000				
2	Co	3.364363	0.000000			
3	S	3.277688	3.446507	0.000000		
4	S	2.311257	2.281319	3.185129	0.000000	
5	B	3.233483	2.094400	1.860631	1.905581	0.000000
6	B	1.924266	2.023616	3.940579	1.982234	3.060080
7	B	3.206226	2.089753	1.939022	3.105417	1.956912
8	B	2.129342	2.158541	3.057961	2.877758	2.886661
9	B	2.049627	3.272109	1.882404	3.168770	2.749207
		6	7	8	9	
6	B	0.000000				
7	B	2.991817	0.000000			
8	B	1.742196	1.758393	0.000000		
9	B	2.960279	1.847104	1.736808	0.000000	

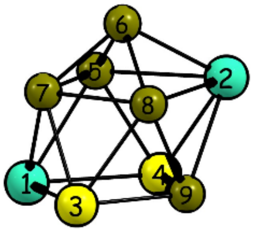

3. -4075.056443 +3.6 C_1

WBI: Co1-Co2: 0.1125

		1	2	3	4	5
1	Co	0.000000				
2	Co	3.707902	0.000000			
3	S	2.237365	3.447782	0.000000		
4	S	2.220653	2.247666	2.949099	0.000000	
5	B	2.123918	2.271589	3.084256	2.012051	0.000000
6	B	3.286691	1.996357	3.092943	3.124330	1.713995
7	B	2.105025	3.061727	1.985619	3.054190	1.839782
8	B	3.312004	2.117584	2.012534	3.086020	2.668777
9	B	2.896167	2.076784	1.926945	1.906620	2.857335
		6	7	8	9	
6	B	0.000000				
7	B	1.686846	0.000000			
8	B	1.688184	1.912096	0.000000		
9	B	2.929922	2.838035	1.878409	0.000000	

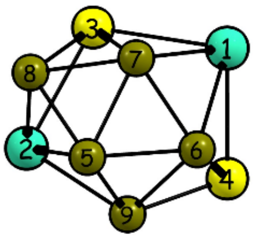

4. -4075.056001 +3.9 C_1

WBI: Co1-Co2: 0.0818

		1	2	3	4	5
1	Co	0.000000				
2	Co	3.689387	0.000000			
3	S	2.249960	2.255730	0.000000		
4	S	2.190564	3.326652	3.314625	0.000000	
5	B	3.260073	2.132127	2.810912	3.046385	0.000000
6	B	2.151228	3.246401	3.086948	1.893887	1.806864
7	B	2.102567	2.972202	2.021766	3.152176	1.798157
8	B	3.423637	1.938606	1.925035	3.987882	1.708314
9	B	3.174481	2.050788	3.151683	1.855513	1.747211

		6	7	8	9
6	B	0.000000			
7	B	1.799933	0.000000		
8	B	3.000206	1.779766	0.000000	
9	B	1.907737	2.865835	2.957711	0.000000

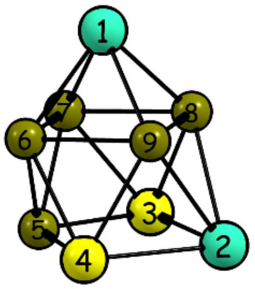

5. -4075.052583 +6.0 C_s

WBI: Co1-Co2: 0.1031

		1	2	3	4	5
1	Co	0.000000				
2	Co	3.625943	0.000000			
3	S	3.438225	2.225166	0.000000		
4	S	3.438467	2.224375	2.967963	0.000000	
5	B	3.250206	2.881917	1.923730	1.924040	0.000000
6	B	1.960880	3.322996	3.059099	2.037645	1.816057
7	B	1.960425	3.324569	2.039606	3.058305	1.814910
8	B	1.963113	2.135067	1.996085	3.086311	2.844119
9	B	1.963775	2.134438	3.086441	1.995791	2.844256

		6	7	8	9
6	B	0.000000			
7	B	1.751931	0.000000		
8	B	2.669701	1.964883	0.000000	
9	B	1.962865	2.670002	1.867257	0.000000

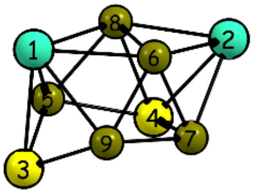

6. -4075.052218 +6.2 C_1

WBI: Co1-Co2: 0.1046

		1	2	3	4	5
1	Co	0.000000				
2	Co	3.710827	0.000000			
3	S	2.189792	4.367299	0.000000		
4	S	3.426525	2.197470	3.085256	0.000000	
5	B	2.086052	3.421722	1.853375	1.977934	0.000000
6	B	2.135596	2.029639	3.118840	2.777119	2.868297
7	B	3.249925	2.006513	3.007197	1.925966	2.906019
8	B	2.076469	2.030033	3.180845	2.080046	1.880680
9	B	2.142155	3.375332	1.848903	3.034094	2.685163

		6	7	8	9
6	B	0.000000			
7	B	1.758639	0.000000		
8	B	1.813772	2.567277	0.000000	
9	B	1.763372	1.862645	2.853532	0.000000

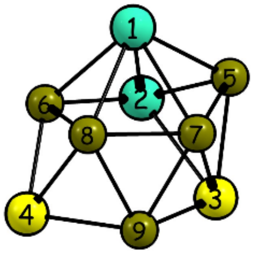

7. -4075.044672 +11.0 C_1

WBI: Co1-Co2: 0.3474

		1	2	3	4	5
1	Co	0.000000				
2	Co	2.484054	0.000000			
3	S	3.211731	2.260789	0.000000		
4	S	3.408094	3.282381	3.151887	0.000000	
5	B	1.959226	1.943847	1.941835	3.954256	0.000000
6	B	2.046315	2.036405	3.112202	1.900231	2.972199
7	B	2.053873	3.013971	2.040682	3.091382	1.847349
8	B	2.047787	3.274981	3.035762	1.925971	3.033207
9	B	3.226273	3.256802	1.922672	1.866035	3.062346
		6	7	8	9	
6	B	0.000000				
7	B	2.875130	0.000000			
8	B	1.963171	1.749887	0.000000		
9	B	2.760233	1.814701	1.828412	0.000000	

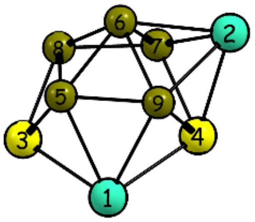

8. -4075.043498 +11.7 C_1

WBI: Co1-Co2: 0.1098

		1	2	3	4	5
1	Co	0.000000				
2	Co	3.778299	0.000000			
3	S	2.177855	4.407935	0.000000		
4	S	2.257454	2.208675	3.230128	0.000000	
5	B	2.124074	3.377181	1.908332	3.050800	0.000000
6	B	3.254283	2.031419	3.035888	2.766988	1.821978
7	B	3.332924	1.993718	3.068936	1.909501	2.873613
8	B	3.138163	3.351712	1.824840	3.043539	1.915605
9	B	2.126787	2.033052	3.178253	2.046140	1.815678
		6	7	8	9	
6	B	0.000000				
7	B	1.773121	0.000000			
8	B	1.736294	1.851332	0.000000		
9	B	1.807353	2.549151	2.866563	0.000000	

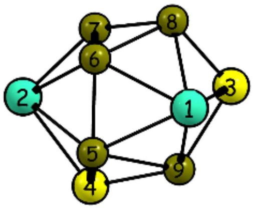

9. -4075.041098 +13.2 C_1

WBI: Co1-Co2: 0.0857

		1	2	3	4	5
1	Co	0.000000				
2	Co	3.703796	0.000000			
3	S	2.220627	3.946943	0.000000		
4	S	3.448958	2.192419	3.300574	0.000000	
5	B	2.127338	2.111421	3.206625	1.915442	0.000000
6	B	2.101844	2.052430	3.000396	3.080524	1.794563
7	B	3.225376	1.896888	2.810322	3.001523	2.814904
8	B	2.064101	3.260812	1.863090	3.729405	2.966880
9	B	2.116237	3.264936	1.874735	1.859639	1.983257
		6	7	8	9	
6	B	0.000000				
7	B	1.726131	0.000000			
8	B	1.738256	1.799010	0.000000		
9	B	2.884627	3.142239	2.870603	0.000000	

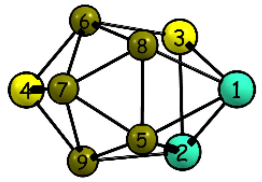

10. -4075.040078 +13.8 C_1

WBI: Co1-Co2: 0.4727

		1	2	3	4	5
1	Co	0.000000				
2	Co	2.354954	0.000000			
3	S	2.214434	2.240397	0.000000		
4	S	4.397353	3.269127	3.154958	0.000000	
5	B	2.088930	2.120419	2.817465	3.069522	0.000000
6	B	3.400416	3.249723	1.953186	1.856314	2.860649
7	B	3.365756	3.222838	3.047018	1.922240	1.815255
8	B	2.034215	2.986108	2.046757	3.084253	1.824349
9	B	3.442651	2.080977	3.128775	1.877687	1.730741

		6	7	8	9
6	B	0.000000			
7	B	1.839268	0.000000		
8	B	1.808684	1.758842	0.000000	
9	B	2.732006	1.851763	2.835111	0.000000

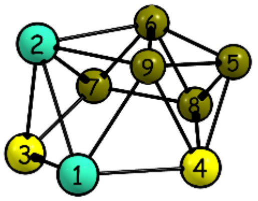

11. -4075.039602 +14.1 C_1

WBI: Co1-Co2: 0.4029

		1	2	3	4	5
1	Co	0.000000				
2	Co	2.494190	0.000000			
3	S	2.162111	2.165909	0.000000		
4	S	2.254146	3.494159	3.202348	0.000000	
5	B	3.438219	3.366280	3.993166	1.898269	0.000000
6	B	3.337550	2.142817	3.123948	2.744588	1.679637
7	B	3.131489	2.210839	1.833571	2.976544	2.953398
8	B	3.375392	3.284629	3.068780	1.938750	1.712575
9	B	2.199205	2.095806	3.193468	2.098302	1.768801

		6	7	8	9
6	B	0.000000			
7	B	1.799263	0.000000		
8	B	1.755444	1.782682	0.000000	
9	B	1.802340	2.858902	2.612774	0.000000

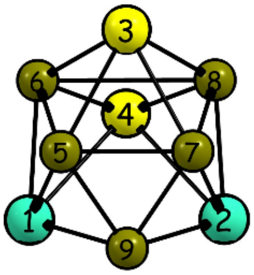

12. -4075.038645 +14.7 C_s

WBI: Co1-Co2: 0.1873

		1	2	3	4	5
1	Co	0.000000				
2	Co	2.782560	0.000000			
3	S	3.428470	3.426348	0.000000		
4	S	2.348944	2.353756	2.951401	0.000000	
5	B	2.071041	3.083705	1.987188	3.098659	0.000000
6	B	2.009112	3.329280	1.982204	1.913278	1.880317
7	B	3.084368	2.068841	1.984206	3.098937	1.878354
8	B	3.328013	2.007919	1.983617	1.912892	2.882042
9	B	2.001224	1.999138	3.127676	3.166128	1.717874

		6	7	8	9
6	B	0.000000			
7	B	2.878481	0.000000		
8	B	2.532123	1.882497	0.000000	
9	B	2.950674	1.718126	2.951657	0.000000

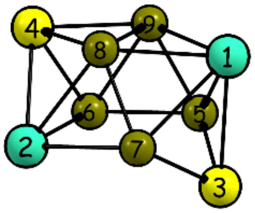

13. -4075.033870 +17.7 C_1

WBI: Co1-Co2: 0.0906

		1	2	3	4	5
1	Co	0.000000				
2	Co	3.648066	0.000000			
3	S	2.197575	3.252019	0.000000		
4	S	3.501891	2.127230	4.112214	0.000000	
5	B	2.152645	3.237579	1.837050	3.146465	0.000000
6	B	3.264141	2.081635	2.960911	1.873789	1.835270
7	B	2.178101	2.088262	1.850397	3.221587	2.709395
8	B	2.105896	2.125913	3.085608	1.967117	2.893045
9	B	2.040779	3.035704	3.066467	1.935062	1.739172

		6	7	8	9
6	B	0.000000			
7	B	2.896029	0.000000		
8	B	2.638377	1.749788	0.000000	
9	B	1.875979	2.896209	1.970332	0.000000

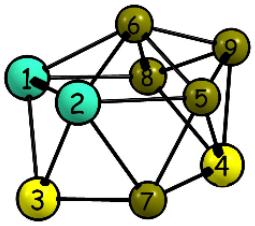

14. -4075.033716 +17.8 C_1

WBI: Co1-Co2: 0.3872

		1	2	3	4	5
1	Co	0.000000				
2	Co	2.504974	0.000000			
3	S	2.156004	2.179545	0.000000		
4	S	3.429880	3.438706	3.143524	0.000000	
5	B	3.296231	2.070858	3.178926	2.066780	0.000000
6	B	2.081619	2.181080	3.196769	2.783100	1.839529
7	B	3.111439	2.109868	1.830702	1.968695	1.876350
8	B	2.073301	3.298753	3.120175	2.000672	2.627932
9	B	3.320792	3.335073	3.990543	1.913838	1.734596

		6	7	8	9
6	B	0.000000			
7	B	2.901858	0.000000		
8	B	1.762698	2.958290	0.000000	
9	B	1.686060	3.036397	1.733384	0.000000

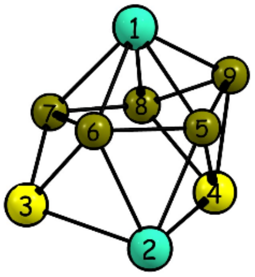

15. -4075.029405 +20.5 C_1

WBI: Co1-Co2: 0.0800

		1	2	3	4	5
1	Co	0.000000				
2	Co	3.644730	0.000000			
3	S	3.355299	2.190976	0.000000		
4	S	3.127808	2.226340	3.249028	0.000000	
5	B	2.071056	2.119963	3.143831	2.035072	0.000000
6	B	2.101895	2.130274	1.882128	3.041330	1.781791
7	B	2.145222	3.184071	1.863093	2.959476	2.957758
8	B	2.132402	3.371589	3.075868	1.968473	2.649077
9	B	1.919313	3.462043	4.015990	1.917022	1.839147

		6	7	8	9
6	B	0.000000			
7	B	2.158968	0.000000		
8	B	2.950951	1.688735	0.000000	
9	B	3.071292	2.972215	1.741729	0.000000

Table S2C: Distance table for the lowest-lying $Cp_2Co_2Se_2B_5H_5$ optimized structures obtained at the PBE0/def2-TZVP level of theory. Included are the zero-point corrected absolute energy in (a.u.) at the DLPNO-CCSD(T)/def2-QZVP level of theory with zero-point energy obtained from the PBE0/def2-TZVP computations, relative energy in (kcal/mol), symmetry and Wiberg bond indecies. For clarity, only the atoms forming the cluster framework are shown.

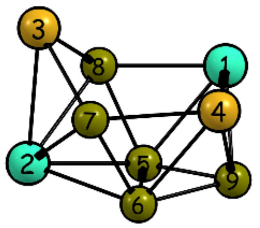

1. -8081.466114 0.0 C_1

WBI: Co1-Co2: 0.1094

		1	2	3	4	5
1	Co	0.000000				
2	Co	3.676713	0.000000			
3	Se	3.411178	2.314233	0.000000		
4	Se	2.411143	3.599780	3.355943	0.000000	
5	B	2.102141	2.129544	3.246631	2.957137	0.000000
6	B	3.022701	2.050830	3.300576	2.192926	1.798342
7	B	3.394652	2.110438	1.997481	2.106399	2.903869
8	B	2.087583	2.198096	2.011410	3.294988	1.758570
9	B	1.963912	3.366153	4.101721	2.048008	1.712482

		6	7	8	9
6	B	0.000000			
7	B	1.878175	0.000000		
8	B	2.881652	2.817366	0.000000	
9	B	1.788550	3.115632	3.001442	0.000000

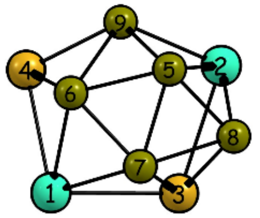

2. -8081.465025 +0.7 C_1

WBI: Co1-Co2: 0.0744

		1	2	3	4	5
1	Co	0.000000				
2	Co	3.823649	0.000000			
3	Se	2.369558	2.390936	0.000000		
4	Se	2.317760	3.466902	3.493089	0.000000	
5	B	3.298608	2.126417	2.952809	3.197035	0.000000
6	B	2.162535	3.260734	3.208508	2.046258	1.795398
7	B	2.121941	3.012797	2.166307	3.308037	1.796644
8	B	3.496134	1.949010	2.064968	4.146640	1.704442
9	B	3.270885	2.056098	3.290232	2.001259	1.736402

		6	7	8	9
6	B	0.000000			
7	B	1.791173	0.000000		
8	B	3.000719	1.802608	0.000000	
9	B	1.919239	2.886008	2.963111	0.000000

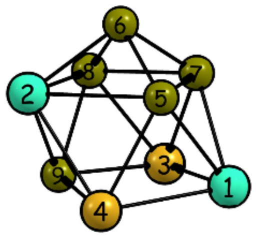

3. -8081.464604 +0.9 C_1

WBI: Co1-Co2: 0.1231

		1	2	3	4	5
1	Co	0.000000				
2	Co	3.772108	0.000000			
3	Se	2.357645	3.604448	0.000000		
4	Se	2.338764	2.363786	3.144858	0.000000	
5	B	2.133009	2.316357	3.241109	2.172586	0.000000
6	B	3.304045	1.998431	3.242909	3.263358	1.709820
7	B	2.111373	3.082117	2.136656	3.194007	1.816398
8	B	3.367239	2.114056	2.149716	3.213262	2.687633
9	B	3.075587	2.080036	2.076586	2.060031	2.967404

		6	7	8	9
6	B	0.000000			
7	B	1.684832	0.000000		
8	B	1.690369	1.947744	0.000000	
9	B	2.956563	2.927297	1.866028	0.000000

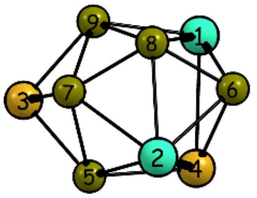

4. -8081.461757 +2.7 C_1

WBI: Co1-Co2: 0.0702

		1	2	3	4	5
1	Co	0.000000				
2	Co	3.393555	0.000000			
3	Se	3.429667	3.620951	0.000000		
4	Se	2.436877	2.392804	3.396696	0.000000	
5	B	3.367285	2.098339	2.008809	2.055698	0.000000
6	B	1.934100	2.051742	4.131631	2.117295	3.158514
7	B	3.217719	2.086383	2.085807	3.218073	1.937280
8	B	2.121897	2.153584	3.217964	3.003483	2.923920
9	B	2.056842	3.293155	2.025995	3.310001	2.839445

		6	7	8	9
6	B	0.000000			
7	B	3.005205	0.000000		
8	B	1.735478	1.756971	0.000000	
9	B	2.974861	1.861183	1.731344	0.000000

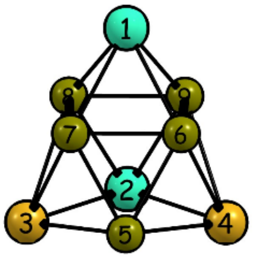

5. -8081.460246 +3.7 C_s

WBI: Co1-Co2: 0.1115

		1	2	3	4	5
1	Co	0.000000				
2	Co	3.650591	0.000000			
3	Se	3.587572	2.347025	0.000000		
4	Se	3.586608	2.345478	3.161318	0.000000	
5	B	3.274667	3.058086	2.069139	2.069132	0.000000
6	B	1.959605	3.386704	3.202630	2.171064	1.820605
7	B	1.959246	3.388294	2.173834	3.200877	1.819288
8	B	1.963128	2.144394	2.148118	3.230095	2.932908
9	B	1.963115	2.143399	3.231255	2.148579	2.934321

		6	7	8	9
6	B	0.000000			
7	B	1.749805	0.000000		
8	B	2.684214	1.996240	0.000000	
9	B	1.995720	2.684721	1.841594	0.000000

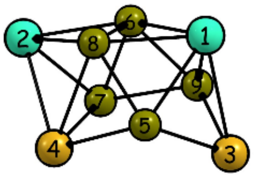

6. -8081.455136 +6.9 C_1

WBI: Co1-Co2: 0.1165

		1	2	3	4	5
1	Co	0.000000				
2	Co	3.723053	0.000000			
3	Se	2.312796	4.524421	0.000000		
4	Se	3.572695	2.307420	3.282097	0.000000	
5	B	2.082376	3.483629	2.004714	2.126352	0.000000
6	B	2.131112	2.021344	3.259028	2.914050	2.898347
7	B	3.279684	2.015601	3.164512	2.080791	3.039782
8	B	2.081153	2.054114	3.326274	2.212974	1.875546
9	B	2.160367	3.378467	1.992804	3.183578	2.784105

		6	7	8	9
6	B	0.000000			
7	B	1.753381	0.000000		
8	B	1.810448	2.611777	0.000000	
9	B	1.756031	1.867171	2.875333	0.000000

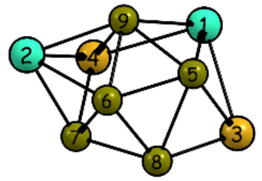

7. -8081.451113 +9.4 C_1

WBI: Co1-Co2: 0.1180

		1	2	3	4	5
1	Co	0.000000				
2	Co	3.821262	0.000000			
3	Se	2.307610	4.557395	0.000000		
4	Se	2.373591	2.321838	3.410498	0.000000	
5	B	2.135271	3.371036	2.057243	3.180514	0.000000
6	B	3.283591	2.027992	3.184261	2.911509	1.809370
7	B	3.459765	2.000092	3.223180	2.065047	2.895020
8	B	3.226390	3.351064	1.967088	3.177675	1.929021
9	B	2.141578	2.051649	3.336603	2.212259	1.804191

		6	7	8	9
6	B	0.000000			
7	B	1.767677	0.000000		
8	B	1.728558	1.852742	0.000000	
9	B	1.804840	2.608834	2.890504	0.000000

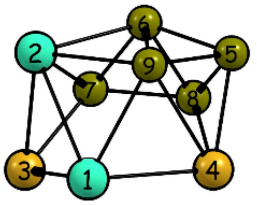

8. -8081.447518 +11.6 C_1

WBI: Co1-Co2: 0.4185

		1	2	3	4	5
1	Co	0.000000				
2	Co	2.520412	0.000000			
3	Se	2.289522	2.293297	0.000000		
4	Se	2.371997	3.642383	3.382265	0.000000	
5	B	3.503538	3.371300	4.134586	2.032515	0.000000
6	B	3.376468	2.127299	3.254007	2.893888	1.675939
7	B	3.243362	2.232963	1.977421	3.121194	2.952492
8	B	3.509126	3.308185	3.215912	2.095130	1.717639
9	B	2.223250	2.096173	3.339122	2.254957	1.791534

		6	7	8	9
6	B	0.000000			
7	B	1.787256	0.000000		
8	B	1.750981	1.782719	0.000000	
9	B	1.798340	2.888193	2.660472	0.000000

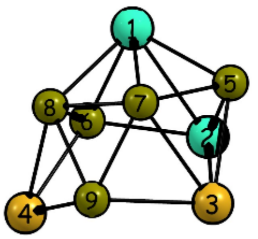

9. -8081.446363 +12.4 C_1

WBI: Co1-Co2: 0.3146

		1	2	3	4	5
1	Co	0.000000				
2	Co	2.484469	0.000000			
3	Se	3.363434	2.397532	0.000000		
4	Se	3.574363	3.433779	3.363783	0.000000	
5	B	1.957404	1.950182	2.078852	4.137239	0.000000
6	B	2.040612	2.040713	3.267646	2.053789	2.981695
7	B	2.051389	3.042486	2.168543	3.254655	1.878293
8	B	2.037675	3.280658	3.166437	2.070829	3.045805
9	B	3.262444	3.381803	2.065041	2.012413	3.147354

		6	7	8	9
6	B	0.000000			
7	B	2.892515	0.000000		
8	B	1.964870	1.750443	0.000000	
9	B	2.854052	1.820734	1.828066	0.000000

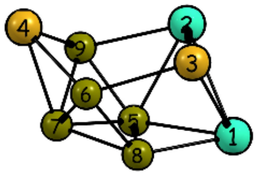

10. -8081.445966 +12.6 C_1

WBI: Co1-Co2: 0.4806

		1	2	3	4	5
1	Co	0.000000				
2	Co	2.377798	0.000000			
3	Se	2.322515	2.365153	0.000000		
4	Se	4.573307	3.423751	3.362481	0.000000	
5	B	2.081867	2.117752	2.944579	3.223734	0.000000
6	B	3.467281	3.382494	2.105847	2.001629	2.892041
7	B	3.372948	3.241095	3.178422	2.064284	1.807051
8	B	2.057630	3.029617	2.180742	3.242401	1.821598
9	B	3.454406	2.088821	3.272780	2.027634	1.724602

		6	7	8	9
6	B	0.000000			
7	B	1.837070	0.000000		
8	B	1.810224	1.757247	0.000000	
9	B	2.823198	1.861713	2.857139	0.000000

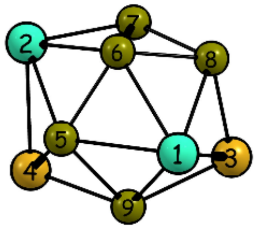

11. -8081.444687 +13.4 C_1

WBI: Co1-Co2: 0.0966

		1	2	3	4	5
1	Co	0.000000				
2	Co	3.718204	0.000000			
3	Se	2.345565	4.064015	0.000000		
4	Se	3.628831	2.317913	3.504590	0.000000	
5	B	2.123217	2.127987	3.318900	2.076516	0.000000
6	B	2.105383	2.045294	3.125873	3.250832	1.787678
7	B	3.221588	1.910161	2.876442	3.138496	2.811919
8	B	2.079870	3.273099	2.006371	3.934142	2.975840
9	B	2.100818	3.331014	2.033517	2.008618	1.947658

		6	7	8	9
6	B	0.000000			
7	B	1.725784	0.000000		
8	B	1.730312	1.794511	0.000000	
9	B	2.911819	3.219118	2.978667	0.000000

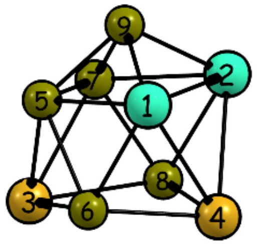

12. -8081.444349 +13.6 C_s

WBI: Co1-Co2: 0.1628

		1	2	3	4	5
1	Co	0.000000				
2	Co	2.872619	0.000000			
3	Se	3.589176	3.592790	0.000000		
4	Se	2.463758	2.458387	3.166952	0.000000	
5	B	2.064644	3.119238	2.122615	3.218546	0.000000
6	B	2.005647	3.441013	2.127623	2.071621	1.878768
7	B	3.119754	2.069120	2.124578	3.218397	1.900447
8	B	3.443890	2.008291	2.127476	2.073989	2.947917
9	B	2.006747	2.004720	3.275164	3.251840	1.718526

		6	7	8	9
6	B	0.000000			
7	B	2.950575	0.000000		
8	B	2.723159	1.875077	0.000000	
9	B	2.965204	1.717433	2.962278	0.000000

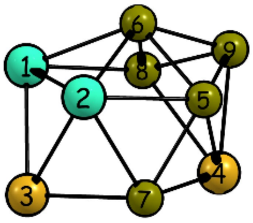

13. -8081.439628 +16.6 C_1

WBI: Co1-Co2: 0.3903

		1	2	3	4	5
1	Co	0.000000				
2	Co	2.540159	0.000000			
3	Se	2.285550	2.305478	0.000000		
4	Se	3.595796	3.589263	3.346999	0.000000	
5	B	3.329245	2.078153	3.325159	2.195538	0.000000
6	B	2.077789	2.168499	3.322836	2.930531	1.835009
7	B	3.222198	2.107045	1.981066	2.109470	1.872083
8	B	2.078849	3.331899	3.277173	2.168132	2.666891
9	B	3.329018	3.347574	4.142662	2.042939	1.753365

		6	7	8	9
6	B	0.000000			
7	B	2.925625	0.000000		
8	B	1.758393	3.089982	0.000000	
9	B	1.683549	3.096012	1.739323	0.000000

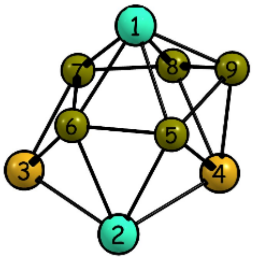

14. -8081.434165 +20.0 C_1

WBI: Co1-Co2: 0.0883

		1	2	3	4	5
1	Co	0.000000				
2	Co	3.684441	0.000000			
3	Se	3.523115	2.318972	0.000000		
4	Se	3.287483	2.345191	3.440026	0.000000	
5	B	2.072050	2.138968	3.312047	2.181486	0.000000
6	B	2.089265	2.138625	2.038453	3.164353	1.776103
7	B	2.132530	3.285669	2.007880	3.099876	2.978082
8	B	2.127412	3.506557	3.230317	2.129873	2.696174
9	B	1.919510	3.534220	4.178986	2.051137	1.868671

		6	7	8	9
6	B	0.000000			
7	B	2.157816	0.000000		
8	B	2.960380	1.688195	0.000000	
9	B	3.070278	2.966418	1.742497	0.000000

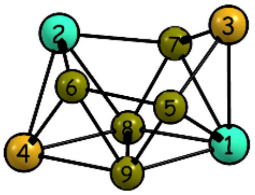

15. -8081.429046 +23.2 C_1

WBI: Co1-Co2: 0.0923

		1	2	3	4	5
1	Co	0.000000				
2	Co	3.656822	0.000000			
3	Se	2.326048	3.350464	0.000000		
4	Se	3.665512	2.257499	4.420637	0.000000	
5	B	2.164556	3.295251	1.972569	3.312180	0.000000
6	B	3.260121	2.072229	3.014094	2.032767	1.842788
7	B	2.188976	2.089620	1.998353	3.394219	2.797158
8	B	2.100470	2.145132	3.230856	2.123765	2.924884
9	B	2.042043	3.054141	3.200282	2.069511	1.724745

		6	7	8	9
6	B	0.000000			
7	B	2.890930	0.000000		
8	B	2.651203	1.737555	0.000000	
9	B	1.906164	2.914668	1.976899	0.000000

Table S3A: Initial 10-vertex $[BH]_{10}{}^{2-}$ polyhedra upon which the starting structures are based; the H atoms are omitted for clarity.

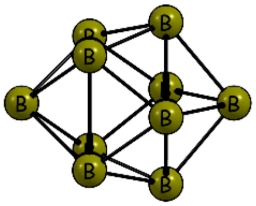 1. Bicapped square antiprism	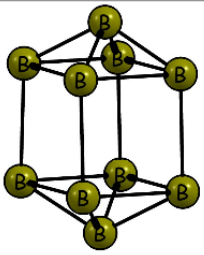 2. Bicapped cube A	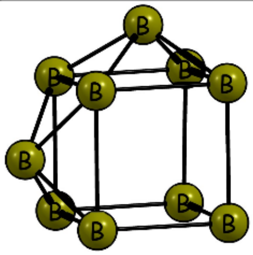 3. Bicapped cube B
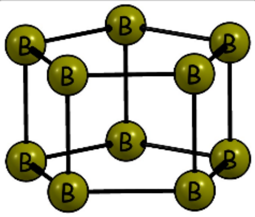 4. Pentagonal prism	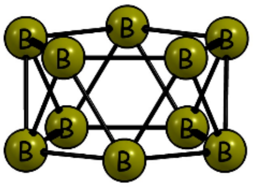 5. Pentagonal antiprism	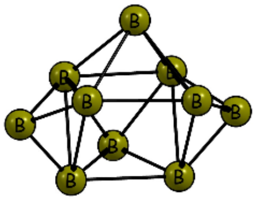 6. Tetracapped trig prism A
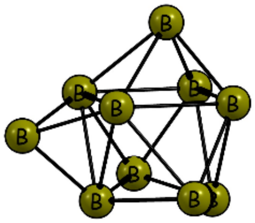 7. Tetracapped trig prism B	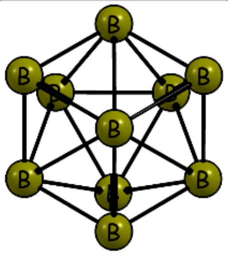 8. Isocloso	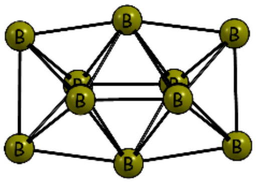 9. Tetracapped octahedron
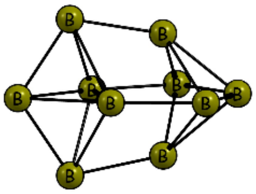 10. Bisdisphenoid-like	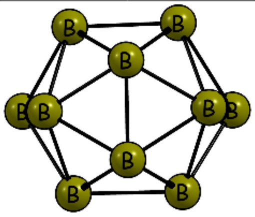 11. Edge-coalesced icosahedron -1vx	

Table S3B: Distance table for the lowest-lying $Cp_2Co_2S_2B_6H_6$ optimized structures obtained at the PBE0/def2-TZVP level of theory. Included are the zero-point corrected absolute energy in (a.u.) at the DLPNO-CCSD(T)/def2-QZVP level of theory with zero-point energy obtained from the PBE0/def2-TZVP computations, relative energy in (kcal/mol), symmetry and Wiberg bond indecies. For clarity, only the atoms forming the cluster framework are shown.

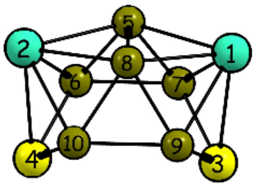

1. -4100.502733 0.0 C_{2v}

WBI: Co1-Co2: 0.0883

		1	2	3	4	5
1	Co	0.000000				
2	Co	3.769219	0.000000			
3	S	2.199202	4.194629	0.000000		
4	S	4.195471	2.198408	3.386299	0.000000	
5	B	2.094656	2.094505	3.037590	3.038404	0.000000
6	B	3.382191	2.088904	3.123078	1.845623	1.760722
7	B	2.090568	3.381632	1.845592	3.124581	1.760426
8	B	2.096404	2.095274	3.039312	3.038216	1.800531
9	B	2.090329	3.382653	1.845957	3.123976	2.846495
10	B	3.383261	2.091124	3.124913	1.845911	2.847583

		6	7	8	9	10
6	B	0.000000				
7	B	1.875807	0.000000			
8	B	2.847366	2.848565	0.000000		
9	B	3.354248	2.781616	1.760963	0.000000	
10	B	2.781621	3.356196	1.760481	1.876586	0.000000

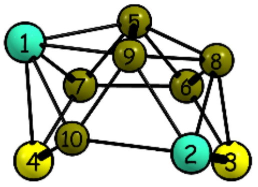

2. -4100.482916 +12.4 C_1

WBI: Co1-Co2: 0.0889

		1	2	3	4	5
1	Co	0.000000				
2	Co	3.755994	0.000000			
3	S	4.238964	2.148600	0.000000		
4	S	2.205961	3.446871	3.479973	0.000000	
5	B	2.079911	3.249850	2.991372	3.030817	0.000000
6	B	3.370174	3.212220	1.812795	3.143289	1.738276
7	B	2.075941	3.787473	3.137854	1.840646	1.767571
8	B	3.381367	2.107970	1.947992	3.757639	1.784172
9	B	2.108339	2.058417	3.086487	3.040460	1.820265
10	B	2.115355	2.137088	3.274324	1.853011	2.870159

		6	7	8	9	10
6	B	0.000000				
7	B	1.875475	0.000000			
8	B	1.863909	3.010107	0.000000		
9	B	2.890006	2.873677	1.790057	0.000000	
10	B	3.425693	2.810600	3.016209	1.771860	0.000000

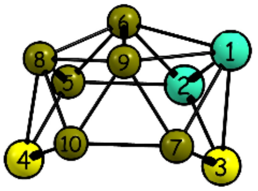

3. -4100.473863 +18.1 C_1

WBI: Co1-Co2: 0.4119

		1	2	3	4	5
1	Co	0.000000				
2	Co	2.459003	0.000000			
3	S	2.198945	2.146729	0.000000		
4	S	4.211529	3.453828	3.499450	0.000000	
5	B	3.425881	2.121140	3.254948	1.856591	0.000000
6	B	2.122181	2.103793	3.088326	3.015339	1.737969
7	B	2.107131	3.241087	1.820567	3.150541	3.413626
8	B	3.379145	3.392953	3.806199	1.951728	1.821976
9	B	2.091768	3.323741	3.031141	2.989866	2.850056
10	B	3.368419	3.806274	3.138358	1.841971	2.810948

		6	7	8	9	10
6	B	0.000000				
7	B	2.896304	0.000000			
8	B	1.790979	3.002909	0.000000		
9	B	1.839713	1.764430	1.761707	0.000000	
10	B	2.865067	1.867230	1.827268	1.723451	0.000000

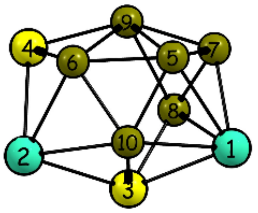

4. -4100.468329 +21.6 C_1

WBI: Co1-Co2: 0.1036

		1	2	3	4	5
1	Co	0.000000				
2	Co	3.810941	0.000000			
3	S	2.222970	2.226720	0.000000		
4	S	4.253316	2.199478	3.179649	0.000000	
5	B	2.122659	3.345465	3.035495	3.119971	0.000000
6	B	3.405794	2.083767	3.060382	1.895390	1.829635
7	B	2.001402	4.091136	3.018507	3.416681	1.709326
8	B	2.166140	3.398183	1.877648	3.032841	2.688211
9	B	3.133871	3.236318	3.073206	1.870982	1.932535
10	B	2.122125	2.111896	2.023279	3.104563	1.766737

		6	7	8	9	10
6	B	0.000000				
7	B	3.040113	0.000000			
8	B	3.277086	1.670480	0.000000		
9	B	2.068260	1.685024	2.045810	0.000000	
10	B	1.785322	2.886943	2.877863	2.844891	0.000000

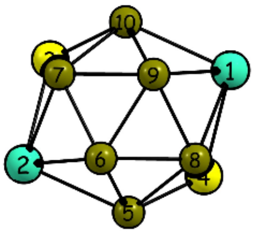

5. -4100.465561 +23.3 C_2

WBI: Co1-Co2: 0.0946

		1	2	3	4	5
1	Co	0.000000				
2	Co	4.200512	0.000000			
3	S	3.433294	2.147021	0.000000		
4	S	2.147059	3.434666	3.572097	0.000000	
5	B	3.238791	2.142930	3.325202	1.827407	0.000000
6	B	3.278906	2.086183	3.091341	3.001984	1.740859
7	B	3.376669	2.120028	1.957312	3.795170	3.014573
8	B	2.120526	3.376492	3.794098	1.957767	1.847644
9	B	2.086927	3.278832	3.001779	3.092170	2.900620
10	B	2.142608	3.238794	1.827386	3.326125	3.519554

		6	7	8	9	10
6	B	0.000000				
7	B	1.778179	0.000000			
8	B	1.782918	2.969656	0.000000		
9	B	1.824656	1.783374	1.777729	0.000000	
10	B	2.900052	1.847116	3.013907	1.740592	0.000000

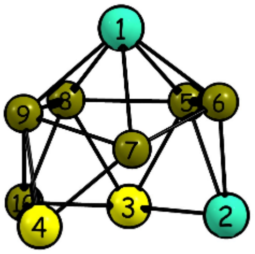

6. -4100.461099 +26.1 C_1

WBI: Co1-Co2: 0.1267

		1	2	3	4	5
1	Co	0.000000				
2	Co	3.393882	0.000000			
3	S	3.399324	2.209462	0.000000		
4	S	3.484355	3.178664	3.143986	0.000000	
5	B	2.062855	2.153738	1.970361	3.774926	0.000000
6	B	1.961010	1.907719	3.131409	3.476196	1.769647
7	B	2.221192	2.327279	3.175932	1.906497	2.739401
8	B	2.012976	3.301524	2.015006	3.075049	1.914895
9	B	2.049431	3.606176	3.054218	1.919880	3.011719
10	B	3.281037	3.246101	1.915971	1.877669	3.049668

		6	7	8	9	10
6	B	0.000000				
7	B	1.749221	0.000000			
8	B	2.991088	2.866233	0.000000		
9	B	3.085844	2.023348	1.761057	0.000000	
10	B	3.684888	2.807333	1.814488	1.804722	0.000000

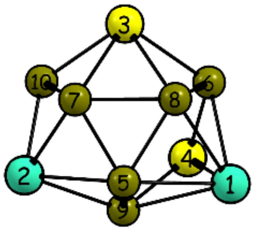

7. -4100.460314 +26.6 C_1

WBI: Co1-Co2: 0.0826

		1	2	3	4	5
1	Co	0.000000				
2	Co	3.755690	0.000000			
3	S	3.497129	3.307572	0.000000		
4	S	2.198395	3.555897	3.243725	0.000000	
5	B	2.118937	2.034175	3.058267	3.106224	0.000000
6	B	2.065997	3.838783	1.973833	1.844606	2.918199
7	B	3.378262	1.993506	2.052655	3.809065	1.749489
8	B	2.068551	3.235894	1.993153	3.130305	1.777835
9	B	2.192701	2.069139	3.455257	1.880331	1.798942
10	B	3.981299	1.850917	1.914380	3.270377	2.834612

		6	7	8	9	10
6	B	0.000000				
7	B	3.084626	0.000000			
8	B	1.894358	1.821924	0.000000		
9	B	2.800109	2.906192	2.872787	0.000000	
10	B	3.055136	1.879150	2.955528	2.780592	0.000000

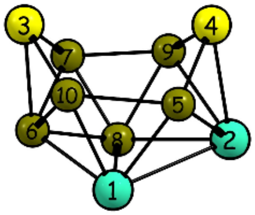

8. -4100.457483 +28.4 C_1

WBI: Co1-Co2: 0.3482

		1	2	3	4	5
1	Co	0.000000				
2	Co	2.540069	0.000000			
3	S	3.350032	4.239278	0.000000		
4	S	3.422609	2.197690	3.320543	0.000000	
5	B	2.019743	2.127712	3.261365	1.835791	0.000000
6	B	2.053755	3.518547	1.964305	3.744594	3.124170
7	B	3.240023	3.369646	1.865762	3.111235	3.457729
8	B	2.125906	2.140903	3.015629	3.051626	2.870776
9	B	3.318768	2.088897	3.028914	1.850052	2.820791
10	B	1.983933	3.503965	1.830846	3.021721	1.971676

		6	7	8	9	10
6	B	0.000000				
7	B	1.781987	0.000000			
8	B	1.772962	1.735391	0.000000		
9	B	2.962488	1.802018	1.814263	0.000000	
10	B	1.911399	2.805436	2.857330	3.302765	0.000000

Table S3C: Distance table for the lowest-lying $Cp_2Co_2Se_2B_6H_6$ optimized structures obtained at the PBE0/def2-TZVP level of theory. Included are the zero-point corrected absolute energy in (a.u.) at the DLPNO-CCSD(T)/def2-QZVP level of theory with zero-point energy obtained from the PBE0/def2-TZVP computations, relative energy in (kcal/mol), symmetry and Wiberg bond indecies. For clarity, only the atoms forming the cluster framework are shown.

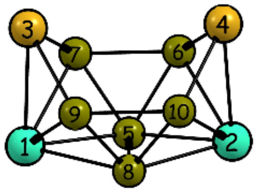

1. -8106.900023 0.0 C_{2v}

WBI: Co1-Co2: 0.1053

		1	2	3	4	5
1	Co	0.000000				
2	Co	3.767693	0.000000			
3	Se	2.318604	4.325032	0.000000		
4	Se	4.326396	2.317660	3.540115	0.000000	
5	B	2.093380	2.093415	3.174965	3.176184	0.000000
6	B	3.395286	2.101941	3.261481	1.992313	1.752904
7	B	2.103726	3.394490	1.992063	3.263275	1.752391
8	B	2.095033	2.094201	3.176563	3.175930	1.799218
9	B	2.103475	3.395589	1.992008	3.262423	2.870748
10	B	3.396330	2.103790	3.263556	1.992524	2.871809

		6	7	8	9	10
6	B	0.000000				
7	B	1.885333	0.000000			
8	B	2.871789	2.872755	0.000000		
9	B	3.438392	2.876218	1.753041	0.000000	
10	B	2.876483	3.440513	1.752412	1.886374	0.000000

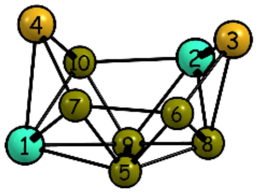

2. -8106.883130 +10.6 C_1

WBI: Co1-Co2: 0.0951

		1	2	3	4	5
1	Co	0.000000				
2	Co	3.775956	0.000000			
3	Se	4.379072	2.280975	0.000000		
4	Se	2.323842	3.568231	3.633939	0.000000	
5	B	2.080946	3.276446	3.138472	3.168053	0.000000
6	B	3.381227	3.297569	1.957474	3.278974	1.729559
7	B	2.092312	3.864816	3.268383	1.986895	1.762049
8	B	3.376381	2.131669	2.093218	3.877663	1.776505
9	B	2.112481	2.061060	3.234364	3.173652	1.819586
10	B	2.125337	2.152507	3.431144	1.998832	2.895756

		6	7	8	9	10
6	B	0.000000				
7	B	1.879073	0.000000			
8	B	1.873935	3.015612	0.000000		
9	B	2.909616	2.898773	1.783836	0.000000	
10	B	3.512157	2.908446	3.030556	1.760518	0.000000

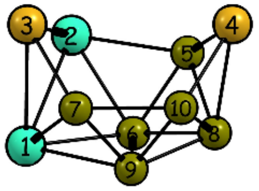

3. -8106.874979 +15.7 C_1

WBI: Co1-Co2: 0.4054

		1	2	3	4	5
1	Co	0.000000				
2	Co	2.489111	0.000000			
3	Se	2.321509	2.275530	0.000000		
4	Se	4.358189	3.590393	3.650857	0.000000	
5	B	3.447570	2.133033	3.386412	2.004855	0.000000
6	B	2.124170	2.103210	3.215258	3.163839	1.732169
7	B	2.117848	3.344786	1.966421	3.296604	3.496724
8	B	3.378317	3.406097	3.918693	2.089415	1.831881
9	B	2.093004	3.354348	3.162620	3.140062	2.873780
10	B	3.382232	3.889187	3.258244	1.987809	2.896131

		6	7	8	9	10
6	B	0.000000				
7	B	2.915831	0.000000			
8	B	1.788253	3.008606	0.000000		
9	B	1.836635	1.752393	1.757888	0.000000	
10	B	2.887481	1.871635	1.841186	1.720632	0.000000

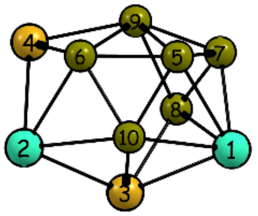

4. -8106.873805 +16.4 C_1

WBI: Co1-Co2: 0.1251

		1	2	3	4	5
1	Co	0.000000				
2	Co	3.871059	0.000000			
3	Se	2.341161	2.343754	0.000000		
4	Se	4.401381	2.328316	3.334849	0.000000	
5	B	2.124982	3.366253	3.182149	3.294410	0.000000
6	B	3.392410	2.090748	3.170175	2.061752	1.802913
7	B	1.992783	4.181059	3.166743	3.575105	1.715639
8	B	2.207730	3.531029	2.023437	3.102925	2.695871
9	B	3.122681	3.319910	3.216636	2.027425	1.896302
10	B	2.152770	2.125896	2.178985	3.280786	1.766091

		6	7	8	9	10
6	B	0.000000				
7	B	3.027706	0.000000			
8	B	3.261784	1.667637	0.000000		
9	B	2.026304	1.689156	2.036011	0.000000	
10	B	1.779639	2.920603	2.955665	2.855553	0.000000

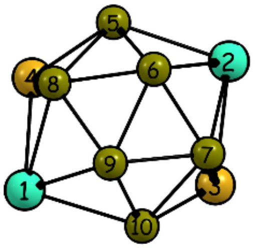

5. -8106.869830 +18.9 C_2

WBI: Co1-Co2: 0.0846

		1	2	3	4	5
1	Co	0.000000				
2	Co	4.245213	0.000000			
3	Se	3.521851	2.280481	0.000000		
4	Se	2.280287	3.521287	3.720027	0.000000	
5	B	3.324690	2.149353	3.494495	1.973368	0.000000
6	B	3.300702	2.095414	3.246465	3.143555	1.727441
7	B	3.379073	2.145637	2.106129	3.920395	3.027067
8	B	2.145580	3.379192	3.921043	2.105912	1.854518
9	B	2.094685	3.301646	3.144270	3.246189	2.922684
10	B	2.149352	3.323917	1.972941	3.493429	3.619825

		6	7	8	9	10
6	B	0.000000				
7	B	1.771158	0.000000			
8	B	1.777234	2.954962	0.000000		
9	B	1.821328	1.777854	1.771567	0.000000	
10	B	2.921461	1.854071	3.026854	1.727121	0.000000

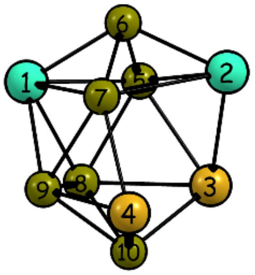

6. -8106.861703 +24.0 C_1

WBI: Co1-Co2: 0.1068

		1	2	3	4	5
1	Co	0.000000				
2	Co	3.407268	0.000000			
3	Se	3.550434	2.337757	0.000000		
4	Se	3.668583	3.280709	3.351863	0.000000	
5	B	2.062017	2.180358	2.108984	3.950555	0.000000
6	B	1.964705	1.908328	3.282257	3.634149	1.764066
7	B	2.203264	2.347055	3.346157	2.068071	2.746493
8	B	2.011268	3.345225	2.142216	3.242185	1.945439
9	B	2.039178	3.601811	3.182072	2.072790	3.012455
10	B	3.314027	3.385137	2.062499	2.025749	3.138902

		6	7	8	9	10
6	B	0.000000				
7	B	1.749154	0.000000			
8	B	3.006601	2.872702	0.000000		
9	B	3.069166	1.991330	1.759950	0.000000	
10	B	3.778198	2.900735	1.821164	1.806289	0.000000

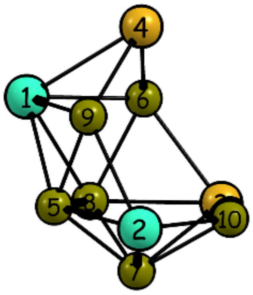

7. -8106.860631 +24.6 C_1

WBI: Co1-Co2: 0.0863

		1	2	3	4	5
1	Co	0.000000				
2	Co	3.771053	0.000000			
3	Se	3.656411	3.455896	0.000000		
4	Se	2.321860	3.682653	3.457156	0.000000	
5	B	2.116810	2.035267	3.209970	3.240201	0.000000
6	B	2.065462	3.938131	2.118909	1.992493	2.947568
7	B	3.394028	1.998101	2.182729	3.953885	1.747005
8	B	2.068860	3.257918	2.133301	3.264912	1.776877
9	B	2.204314	2.074993	3.650237	2.030694	1.782685
10	B	4.078348	1.852034	2.054340	3.464962	2.868221

		6	7	8	9	10
6	B	0.000000				
7	B	3.148544	0.000000			
8	B	1.887323	1.845445	0.000000		
9	B	2.909167	2.922016	2.893789	0.000000	
10	B	3.248464	1.885825	3.031688	2.877182	0.000000

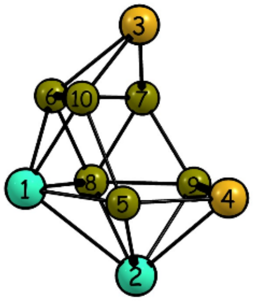

8. -8106.852767 +29.6 C_1

WBI: Co1-Co2: 0.3102

		1	2	3	4	5
1	Co	0.000000				
2	Co	2.548613	0.000000			
3	Se	3.510789	4.389127	0.000000		
4	Se	3.555910	2.318033	3.478732	0.000000	
5	B	2.003301	2.130919	3.456383	1.980077	0.000000
6	B	2.051405	3.511435	2.101335	3.857194	3.148640
7	B	3.266710	3.382278	2.014676	3.255489	3.559557
8	B	2.126127	2.133990	3.167509	3.186476	2.892185
9	B	3.347533	2.104157	3.163739	2.001795	2.921204
10	B	1.981289	3.510538	1.980677	3.112445	1.993917

		6	7	8	9	10
6	B	0.000000				
7	B	1.790734	0.000000			
8	B	1.768092	1.730001	0.000000		
9	B	2.962406	1.806216	1.805132	0.000000	
10	B	1.931621	2.884826	2.873863	3.358885	0.000000

Table S4A: Initial 11-vertex $[BH]_{11}^{2-}$ polyhedra upon which the starting structures are based; the H atoms are omitted for clarity.

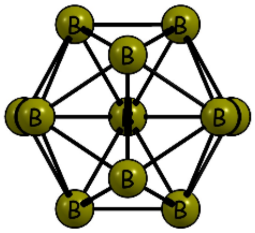 1. Edge-coalesced icosahedron	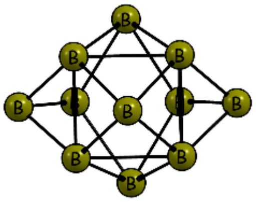 2. Pentacapped trigonal prism	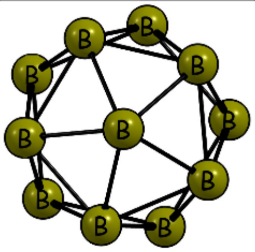 3. Capped pentagonal antiprism
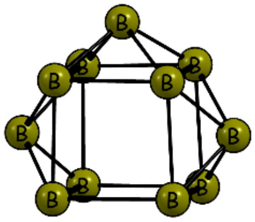 4. Tricapped cube A	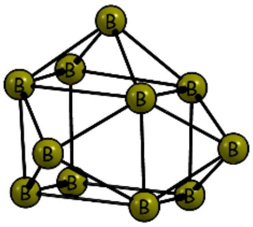 5. Tricapped cube B	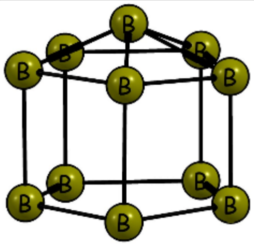 6. Capped pentagonal prism A
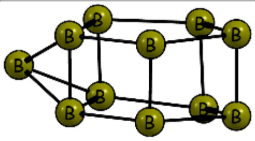 7. Capped pentagonal prism B		

Table S4B: Distance table for the lowest-lying $Cp_2Co_2S_2B_7H_7$ optimized structures obtained at the PBE0/def2-TZVP level of theory. Included are the zero-point corrected absolute energy in (a.u.) at the DLPNO-CCSD(T)/def2-QZVP level of theory with zero-point energy obtained from the PBE0/def2-TZVP computations, relative energy in (kcal/mol), symmetry and Wiberg bond indecies. For clarity, only the atoms forming the cluster framework are shown.

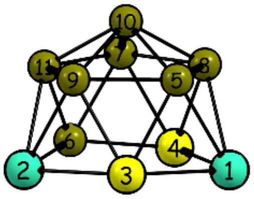

1. -4125.933556 0.0 C_1

WBI: Co1-Co2: 0.1004

		1	2	3	4	5
1	Co	0.000000				
2	Co	3.722021	0.000000			
3	S	2.204061	2.180706	0.000000		
4	S	2.213922	3.397268	3.271137	0.000000	
5	B	2.111192	3.335593	1.954686	3.130602	0.000000
6	B	3.417279	2.045264	3.160236	1.910921	3.487930
7	B	3.399948	3.296821	3.688309	1.989316	2.942391
8	B	2.102400	3.874115	3.116856	1.991348	1.793157
9	B	3.400773	2.069318	1.985834	3.633637	1.874371
10	B	3.284127	3.264665	3.060799	3.057790	1.758552
11	B	3.912852	2.054659	3.115765	3.032818	2.940948
		6	7	8	9	10
6	B	0.000000				
7	B	1.833852	0.000000			
8	B	2.971589	1.847892	0.000000		
9	B	2.907892	2.891912	2.939315	0.000000	
10	B	2.886148	1.756553	1.752887	1.771407	0.000000
11	B	1.737746	1.750595	2.912405	1.776834	1.803819
		11				
11	B	0.000000				

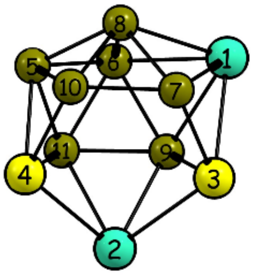

2. -4125.930663 +1.8 C_1

WBI: Co1-Co2: 0.0953

		1	2	3	4	5
1	Co	0.000000				
2	Co	3.746781	0.000000			
3	S	2.221279	2.210361	0.000000		
4	S	4.072047	2.192638	3.197555	0.000000	
5	B	3.305965	3.397321	3.659950	1.987566	0.000000
6	B	2.093094	3.300606	3.114634	3.066093	1.769401
7	B	2.100384	3.375911	1.885440	3.031334	2.925368
8	B	2.073891	3.893579	3.061870	3.024838	1.750336
9	B	2.114199	2.110525	2.026730	3.114439	2.947334
10	B	3.296704	3.380763	3.054489	1.881887	1.801180
11	B	3.318092	2.096713	3.127442	1.998179	1.865571
		6	7	8	9	10
6	B	0.000000				
7	B	2.921277	0.000000			
8	B	1.801686	1.783491	0.000000		
9	B	1.774409	2.969247	2.914532	0.000000	
10	B	2.878017	1.786348	1.735995	3.451633	0.000000
11	B	1.751819	3.454929	2.900695	1.779642	2.945854
		11				
11	B	0.000000				

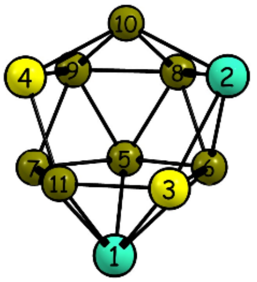

3. -4125.923748 +6.2 C_1

WBI: Co1-Co2: 0.0908

		1	2	3	4	5
1	Co	0.000000				
2	Co	3.730446	0.000000			
3	S	2.244275	2.191142	0.000000		
4	S	3.465086	3.385406	3.144203	0.000000	
5	B	2.075476	3.279924	3.124734	3.076548	0.000000
6	B	2.177734	2.055685	2.054964	3.601906	1.787022
7	B	2.042073	3.906877	3.125849	2.022869	1.756637
8	B	3.336661	2.054437	3.131558	3.041089	1.791147
9	B	3.317149	3.293632	3.653506	1.982678	1.770018
10	B	3.934243	2.009629	3.094157	1.949011	2.904578
11	B	2.072518	3.345612	1.865965	1.928954	2.929755

		6	7	8	9	10
6	B	0.000000				
7	B	2.942043	0.000000			
8	B	1.762213	2.913028	0.000000		
9	B	2.891172	1.832206	1.765292	0.000000	
10	B	2.878251	3.020306	1.737437	1.849079	0.000000
11	B	2.988571	1.886585	3.463300	2.994661	3.033887

		11
11	B	0.000000

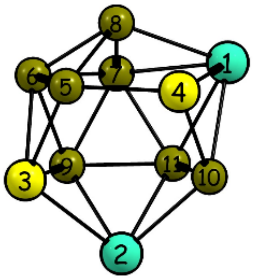

4. -4125.921229 +7.7 C_1

WBI: Co1-Co2: 0.0869

		1	2	3	4	5
1	Co	0.000000				
2	Co	3.687450	0.000000			
3	S	4.074387	2.178225	0.000000		
4	S	2.232333	3.371168	3.129181	0.000000	
5	B	3.307848	3.347765	1.885544	1.909825	0.000000
6	B	3.284135	3.350787	1.993284	3.104750	1.801588
7	B	2.098686	3.267278	3.086511	3.145924	2.905912
8	B	2.083747	3.895473	3.083343	2.092565	1.824337
9	B	3.311333	2.057168	2.015757	3.620370	2.969982
10	B	2.120195	2.041396	3.098530	1.918075	3.016063
11	B	2.097541	2.055696	3.114971	3.076975	3.453072

		6	7	8	9	10
6	B	0.000000				
7	B	1.762270	0.000000			
8	B	1.726429	1.772635	0.000000		
9	B	1.880660	1.763565	2.918483	0.000000	
10	B	3.491307	2.934793	3.028178	2.915860	0.000000
11	B	2.935019	1.793257	2.924389	1.776217	1.778598

		11
11	B	0.000000

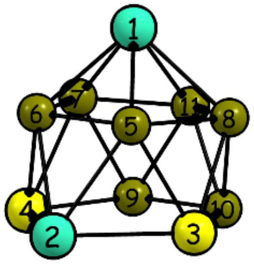

5. -4125.920524 +8.2 C_s

WBI: Co1-Co2: 0.0772

		1	2	3	4	5
1	Co	0.000000				
2	Co	3.647392	0.000000			
3	S	3.434302	2.201599	0.000000		
4	S	3.434021	2.201719	3.214845	0.000000	
5	B	2.022583	2.110343	1.985194	3.129510	0.000000
6	B	2.021668	2.112161	3.130512	1.983494	1.822592
7	B	2.049977	3.425195	3.656166	1.989666	2.993606
8	B	2.049299	3.424596	1.989833	3.656021	1.904962
9	B	3.274223	3.375952	3.036432	1.907040	3.470128
10	B	3.274012	3.375489	1.906178	3.036666	2.979197
11	B	2.077809	3.905334	3.044988	3.045651	2.940469

		6	7	8	9	10
6	B	0.000000				
7	B	1.903159	0.000000			
8	B	2.992737	2.926398	0.000000		
9	B	2.977823	1.821080	2.898662	0.000000	
10	B	3.469062	2.898340	1.821437	1.737401	0.000000
11	B	2.939078	1.758111	1.757529	1.754522	1.754061

		11
11	B	0.000000

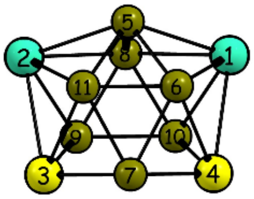

6. -4125.917850 +9.9 C_s

WBI: Co1-Co2: 0.0838

		1	2	3	4	5
1	Co	0.000000				
2	Co	3.724940	0.000000			
3	S	4.051227	2.229057	0.000000		
4	S	2.229769	4.050319	3.070784	0.000000	
5	B	2.109624	2.109218	3.173941	3.172994	0.000000
6	B	2.112458	3.281267	3.121804	2.129619	1.761412
7	B	3.287679	3.285447	1.901581	1.901704	2.909977
8	B	2.085953	2.087820	3.056877	3.056478	1.799593
9	B	3.315955	2.070406	1.886217	3.015713	2.946967
10	B	2.070640	3.315462	3.013417	1.886619	2.946726
11	B	3.282665	2.111612	2.131910	3.120799	1.761835

		6	7	8	9	10
6	B	0.000000				
7	B	1.811253	0.000000			
8	B	2.901033	3.400671	0.000000		
9	B	3.488524	2.985750	1.770753	0.000000	
10	B	3.018869	2.984803	1.770359	1.800529	0.000000
11	B	1.694151	1.810509	2.902145	3.020333	3.488110

		11
11	B	0.000000

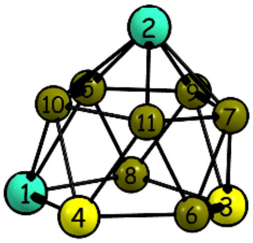

7. -4125.916528 +10.7 C_1

WBI: Co1-Co2: 0.0800

		1	2	3	4	5
1	Co	0.000000				
2	Co	3.648554	0.000000			
3	S	3.366295	3.451358	0.000000		
4	S	2.194382	3.455052	3.151922	0.000000	
5	B	2.062236	2.090895	3.059115	3.142021	0.000000
6	B	3.339037	3.256002	1.901934	1.892272	3.470688
7	B	3.899615	2.011129	2.020336	3.091605	2.957259
8	B	2.023473	3.274400	1.940612	3.124226	1.732517
9	B	3.292650	2.034429	2.027181	3.673157	1.760137
10	B	2.063217	2.055387	3.614143	2.019183	1.797258
11	B	3.372906	2.010299	3.093255	1.991036	2.979611

		6	7	8	9	10
6	B	0.000000				
7	B	1.814699	0.000000			
8	B	3.025739	3.016584	0.000000		
9	B	3.003549	1.898893	1.826887	0.000000	
10	B	2.988977	2.960309	2.897435	2.915323	0.000000
11	B	1.814424	1.758052	3.503158	2.959050	1.914249

		11
11	B	0.000000

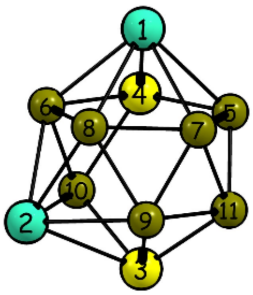

8. -4125.913402 +12.6 C_1

WBI: Co1-Co2: 0.0809

		1	2	3	4	5
1	Co	0.000000				
2	Co	3.704208	0.000000			
3	S	4.033872	2.260057	0.000000		
4	S	2.216505	3.489610	3.076410	0.000000	
5	B	2.130901	3.907524	2.961223	1.911087	0.000000
6	B	2.097477	2.038907	3.113987	2.098441	3.033827
7	B	2.094109	3.297435	3.044929	3.074059	1.802679
8	B	2.119231	2.095908	3.138370	3.166772	2.967172
9	B	3.305387	2.176181	2.088685	3.650924	2.903062
10	B	3.334454	2.065073	1.871121	1.915609	2.979612
11	B	3.292058	3.339297	1.898858	3.035238	1.744927

		6	7	8	9	10
6	B	0.000000				
7	B	2.906236	0.000000			
8	B	1.777580	1.786774	0.000000		
9	B	2.947239	1.713878	1.778727	0.000000	
10	B	1.881555	3.441253	2.961907	3.028406	0.000000
11	B	3.479004	1.739911	2.904833	1.796672	2.993673

		11
11	B	0.000000

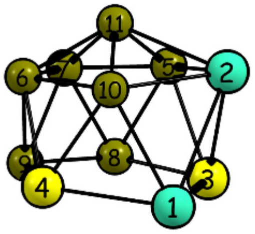

9. -4125.909483 +15.1 C_1

WBI: Co1-Co2: 0.3822

		1	2	3	4	5
1	Co	0.000000				
2	Co	2.491712	0.000000			
3	S	2.196755	2.245975	0.000000		
4	S	2.198757	3.588162	3.206618	0.000000	
5	B	3.498159	2.140703	2.042355	3.647853	0.000000
6	B	3.419807	3.369812	3.629354	1.951541	2.886493
7	B	3.927718	3.307061	3.046821	3.018900	1.724350
8	B	3.373079	3.349475	1.900568	3.036926	1.812816
9	B	3.360552	3.940669	3.024585	1.893165	2.885945
10	B	2.190085	2.120765	3.104768	2.015873	2.952932
11	B	3.399162	2.078783	3.098087	3.066465	1.788192

		6	7	8	9	10
6	B	0.000000				
7	B	1.761260	0.000000			
8	B	2.903776	1.753303	0.000000		
9	B	1.848583	1.752071	1.742775	0.000000	
10	B	1.860202	2.911050	3.453498	2.990068	0.000000
11	B	1.761298	1.793720	2.899551	2.902440	1.761258

		11
11	B	0.000000

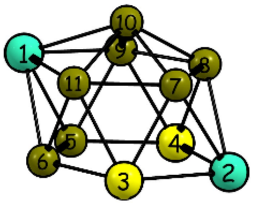

10. -4125.909456 +15.1 C_s

WBI: Co1-Co2: 0.0458

		1	2	3	4	5
1	Co	0.000000				
2	Co	4.363517	0.000000			
3	S	3.433043	2.200165	0.000000		
4	S	3.433023	2.200458	3.205375	0.000000	
5	B	2.074462	3.385388	3.028404	1.918919	0.000000
6	B	2.074085	3.383178	1.919357	3.024582	1.708560
7	B	3.295400	2.117119	1.969031	3.112112	3.471520
8	B	3.296440	2.117018	3.112628	1.969102	2.992136
9	B	2.021469	3.407949	3.639910	1.987733	1.898308
10	B	2.076870	3.304553	3.067419	3.066821	2.948765
11	B	2.019909	3.408194	1.987639	3.639319	2.925973

		6	7	8	9	10
6	B	0.000000				
7	B	2.991060	0.000000			
8	B	3.470054	1.812465	0.000000		
9	B	2.924814	2.942955	1.846012	0.000000	
10	B	2.948322	1.762053	1.762440	1.775065	0.000000
11	B	1.897975	1.846738	2.944464	2.900097	1.776643

		11
11	B	0.000000

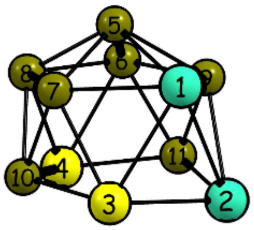

11. -4125.905729 +17.5 C_1

WBI: Co1-Co2: 0.3910

		1	2	3	4	5
1	Co	0.000000				
2	Co	2.452237	0.000000			
3	S	2.250445	2.197556	0.000000		
4	S	4.061894	3.325266	3.142934	0.000000	
5	B	2.099237	3.390512	3.136845	3.080728	0.000000
6	B	3.309781	3.322929	3.663619	2.023952	1.747520
7	B	2.121901	3.452366	2.055036	3.084390	1.762774
8	B	3.298102	3.921507	3.101800	2.027976	1.748160
9	B	2.123422	2.125480	3.121246	3.041897	1.821543
10	B	3.325541	3.331539	1.893604	1.906084	2.894589
11	B	3.356435	2.040702	3.145401	1.914820	2.897952

		6	7	8	9	10
6	B	0.000000				
7	B	2.906586	0.000000			
8	B	1.861031	1.718175	0.000000		
9	B	1.758318	2.945705	2.924139	0.000000	
10	B	2.978891	1.811063	1.811579	3.440047	0.000000
11	B	1.804666	3.504751	2.990604	1.732978	3.030317

		11
11	B	0.000000

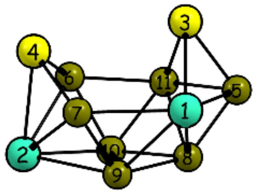

12. -4125.902261 +19.6 C_1

WBI: Co1-Co2: 0.0782

		1	2	3	4	5
1	Co	0.000000				
2	Co	3.826320	0.000000			
3	S	2.266529	4.300043	0.000000		
4	S	3.507947	2.191789	3.177291	0.000000	
5	B	2.099620	4.574452	1.795667	4.290355	0.000000
6	B	3.672401	2.125406	2.955287	1.853921	3.569423
7	B	2.207075	2.136483	3.164172	1.856987	3.710094
8	B	2.148968	3.387637	2.825013	3.918106	1.707493
9	B	2.140997	2.098785	3.441836	3.049543	3.100786
10	B	3.189008	2.064492	3.314059	3.018843	3.003769
11	B	2.928883	3.449625	1.966152	3.247054	1.845181

		6	7	8	9	10
6	B	0.000000				
7	B	2.760847	0.000000			
8	B	3.036939	3.066926	0.000000		
9	B	2.834176	1.756330	1.769380	0.000000	
10	B	1.730417	2.813573	1.754803	1.787717	0.000000
11	B	1.942504	3.351411	1.858465	2.830587	1.784577

		11
11	B	0.000000

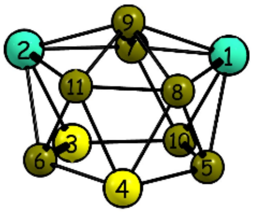

13. -4125.900389 +20.8 C_1

WBI: Co1-Co2: 0.0844

		1	2	3	4	5
1	Co	0.000000				
2	Co	3.674565	0.000000			
3	S	3.453883	2.238857	0.000000		
4	S	3.437191	3.457962	3.092226	0.000000	
5	B	2.076562	3.929602	2.977142	1.956309	0.000000
6	B	3.900908	2.076786	1.873255	1.901002	3.000675
7	B	2.003253	2.148099	2.052841	3.616389	2.907156
8	B	2.021655	3.331486	3.610667	1.997338	1.927381
9	B	2.053919	2.096824	3.134257	3.091917	2.982705
10	B	2.072279	3.409014	1.944857	3.017617	1.675682
11	B	3.276071	2.074718	3.115078	2.008367	3.045964

		6	7	8	9	10
6	B	0.000000				
7	B	3.008361	0.000000			
8	B	2.994598	2.896748	0.000000		
9	B	2.950361	1.806553	1.776384	0.000000	
10	B	3.006597	1.894954	2.925982	2.984600	0.000000
11	B	1.885470	2.944786	1.829337	1.755928	3.507492

		11
11	B	0.000000

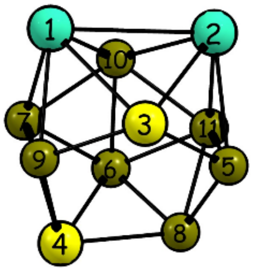

14. -4125.897445 +22.7 C_1

WBI: Co1-Co2: 0.3116

		1	2	3	4	5
1	Co	0.000000				
2	Co	2.560638	0.000000			
3	S	2.368843	2.302645	0.000000		
4	S	3.466913	4.088421	3.097153	0.000000	
5	B	3.484519	2.081169	1.904778	3.004530	0.000000
6	B	3.341198	3.331897	3.617985	1.988690	2.921947
7	B	2.019851	3.409605	3.138616	2.028940	3.495526
8	B	3.961964	3.276652	3.013069	1.900433	1.754320
9	B	2.057675	3.412527	1.873630	1.920157	2.974254
10	B	2.108489	2.147451	3.134339	3.103076	2.951393
11	B	3.394502	2.050994	3.068222	3.026668	1.792615

		6	7	8	9	10
6	B	0.000000				
7	B	1.832302	0.000000			
8	B	1.819986	2.984575	0.000000		
9	B	2.984946	1.883432	2.991628	0.000000	
10	B	1.795859	1.767439	2.916418	2.930549	0.000000
11	B	1.757288	2.902254	1.739322	3.434710	1.806256

		11
11	B	0.000000

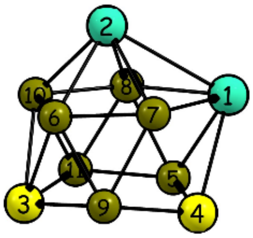

15. -4125.892387 +25.8 C_1

WBI: Co1-Co2: 0.3464

		1	2	3	4	5
1	Co	0.000000				
2	Co	2.534787	0.000000			
3	S	4.066947	3.462917	0.000000		
4	S	2.213376	3.647037	3.075231	0.000000	
5	B	2.102172	3.420966	2.990218	1.896095	0.000000
6	B	3.387323	2.006445	2.021606	3.135486	3.491690
7	B	2.135438	2.024813	3.116441	2.180666	3.050008
8	B	2.123786	2.084183	3.055766	3.078980	1.815901
9	B	3.290091	3.282802	1.907281	1.886029	2.967129
10	B	3.421810	2.033819	2.018851	3.667800	2.931516
11	B	3.300685	3.275652	1.920127	3.011878	1.745580
		6	7	8	9	10
6	B	0.000000				
7	B	1.746124	0.000000			
8	B	2.913601	2.870515	0.000000		
9	B	1.790348	1.845364	3.404407	0.000000	
10	B	1.920474	2.952559	1.772034	2.996442	0.000000
11	B	2.983119	3.473495	1.749873	2.996631	1.778696
		11				
11	B	0.000000				

Table S4C: Distance table for the lowest-lying $Cp_2Co_2Se_2B_7H_7$ optimized structures obtained at the PBE0/def2-TZVP level of theory. Included are the zero-point corrected absolute energy in (a.u.) at the DLPNO-CCSD(T)/def2-QZVP level of theory with zero-point energy obtained from the PBE0/def2-TZVP computations, relative energy in (kcal/mol), symmetry and Wiberg bond indecies. For clarity, only the atoms forming the cluster framework are shown.

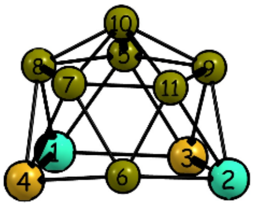

1. -8132.342536 0.0 C_1

WBI: Co1-Co2: 0.0986

		1	2	3	4	5
1	Co	0.000000				
2	Co	3.906022	0.000000			
3	Se	2.324548	2.305738	0.000000		
4	Se	2.331280	3.538842	3.449748	0.000000	
5	B	2.132000	3.414773	2.100186	3.270155	0.000000
6	B	3.592736	2.046644	3.298435	2.057983	3.552204
7	B	3.468517	3.308397	3.825150	2.120237	2.952302
8	B	2.120326	3.944523	3.256039	2.137628	1.785312
9	B	3.483226	2.092361	2.124391	3.783247	1.903493
10	B	3.318326	3.284357	3.203986	3.203780	1.753928
11	B	4.011019	2.053096	3.255225	3.181076	2.959353

		6	7	8	9	10
6	B	0.000000				
7	B	1.848793	0.000000			
8	B	3.053377	1.874914	0.000000		
9	B	2.921252	2.891354	2.957197	0.000000	
10	B	2.904242	1.756384	1.750403	1.770233	0.000000
11	B	1.732498	1.749130	2.936386	1.771345	1.799986

		11
11	B	0.000000

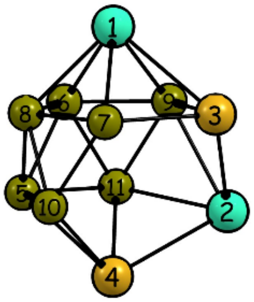

2. -8132.338503 +2.5 C_1

WBI: Co1-Co2: 0.1035

		1	2	3	4	5
1	Co	0.000000				
2	Co	3.805784	0.000000			
3	Se	2.334881	2.328679	0.000000		
4	Se	4.219906	2.312480	3.379633	0.000000	
5	B	3.305513	3.465877	3.799138	2.120693	0.000000
6	B	2.095996	3.327911	3.259789	3.211965	1.767421
7	B	2.121934	3.550611	2.030639	3.180683	2.936685
8	B	2.071439	3.981544	3.199870	3.173270	1.746431
9	B	2.139270	2.130638	2.186629	3.262693	2.960543
10	B	3.307142	3.544584	3.190163	2.025624	1.815077
11	B	3.330978	2.115764	3.272447	2.142881	1.892524

		6	7	8	9	10
6	B	0.000000				
7	B	2.947520	0.000000			
8	B	1.798171	1.777337	0.000000		
9	B	1.767137	3.066957	2.936539	0.000000	
10	B	2.895498	1.785555	1.732402	3.521616	0.000000
11	B	1.747534	3.530700	2.922279	1.772792	3.022338

		11
11	B	0.000000

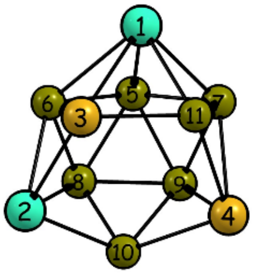

3. -8132.329521 +8.1 C_1

WBI: Co1-Co2: 0.0888

		1	2	3	4	5
1	Co	0.000000				
2	Co	3.791640	0.000000			
3	Se	2.359902	2.314697	0.000000		
4	Se	3.620335	3.532754	3.351105	0.000000	
5	B	2.075508	3.288886	3.262845	3.224084	0.000000
6	B	2.213741	2.072683	2.204064	3.775270	1.784980
7	B	2.044783	3.955406	3.258417	2.157327	1.756515
8	B	3.356022	2.053365	3.278989	3.189889	1.787671
9	B	3.336720	3.306216	3.802851	2.112005	1.768413
10	B	3.997273	2.015619	3.246146	2.089142	2.919094
11	B	2.080064	3.515550	2.011642	2.067833	2.963729

		6	7	8	9	10
6	B	0.000000				
7	B	2.966998	0.000000			
8	B	1.756759	2.930865	0.000000		
9	B	2.896882	1.856826	1.762263	0.000000	
10	B	2.898543	3.083102	1.733256	1.866567	0.000000
11	B	3.093846	1.883103	3.565570	3.065401	3.199151

		11
11	B	0.000000

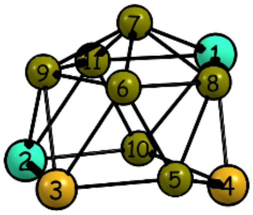

4. -8132.327540 +9.4 C_1

WBI: Co1-Co2: 0.0979

		1	2	3	4	5
1	Co	0.000000				
2	Co	3.705654	0.000000			
3	Se	4.236332	2.305185	0.000000		
4	Se	2.345211	3.515780	3.334239	0.000000	
5	B	3.376226	3.514293	2.030440	2.050486	0.000000
6	B	3.300439	3.417635	2.129489	3.245331	1.805755
7	B	2.101271	3.284371	3.230543	3.284271	2.930431
8	B	2.108422	3.956197	3.232796	2.225497	1.827754
9	B	3.319037	2.077529	2.152921	3.775831	3.051024
10	B	2.137533	2.050635	3.262376	2.062921	3.187443
11	B	2.096914	2.054417	3.261565	3.216672	3.543729

		6	7	8	9	10
6	B	0.000000				
7	B	1.756419	0.000000			
8	B	1.725488	1.768232	0.000000		
9	B	1.910854	1.764114	2.940911	0.000000	
10	B	3.556241	2.953979	3.101558	2.937016	0.000000
11	B	2.950612	1.791960	2.941504	1.771802	1.771115

		11
11	B	0.000000

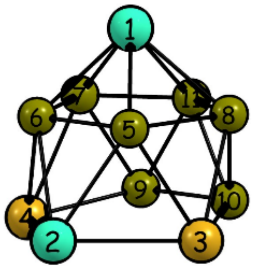

5. -8132.326461 +10.1 C_s

WBI: Co1-Co2: 0.0869

		1	2	3	4	5
1	Co	0.000000				
2	Co	3.681460	0.000000			
3	Se	3.585559	2.321535	0.000000		
4	Se	3.586479	2.322189	3.402156	0.000000	
5	B	2.020313	2.130495	2.131289	3.272868	0.000000
6	B	2.019758	2.131180	3.271519	2.129803	1.812977
7	B	2.052011	3.498716	3.800929	2.122079	3.006788
8	B	2.050749	3.499277	2.122800	3.802378	1.934319
9	B	3.295081	3.547197	3.178338	2.050498	3.536802
10	B	3.294442	3.546818	2.048960	3.179745	3.059432
11	B	2.073369	4.001203	3.190910	3.191781	2.962360

		6	7	8	9	10
6	B	0.000000				
7	B	1.931534	0.000000			
8	B	3.005633	2.923976	0.000000		
9	B	3.057585	1.838547	2.907848	0.000000	
10	B	3.534590	2.907656	1.838439	1.735639	0.000000
11	B	2.960087	1.753774	1.753479	1.749459	1.749444

		11
11	B	0.000000

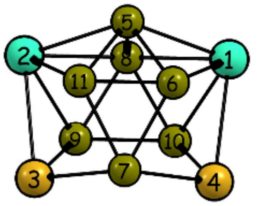

6. -8132.323711 +11.8 C_s

WBI: Co1-Co2: 0.0926

		1	2	3	4	5
1	Co	0.000000				
2	Co	3.734151	0.000000			
3	Se	4.209040	2.344364	0.000000		
4	Se	2.344801	4.208500	3.271609	0.000000	
5	B	2.113760	2.112815	3.315374	3.314761	0.000000
6	B	2.138305	3.300703	3.267640	2.263754	1.755782
7	B	3.356411	3.354439	2.043722	2.043655	2.932094
8	B	2.084709	2.085949	3.199733	3.199873	1.797294
9	B	3.332851	2.084989	2.029711	3.170434	2.963423
10	B	2.085333	3.332103	3.167486	2.030751	2.963277
11	B	3.302303	2.137454	2.265538	3.266579	1.756374

		6	7	8	9	10
6	B	0.000000				
7	B	1.815721	0.000000			
8	B	2.915325	3.484641	0.000000		
9	B	3.548735	3.144108	1.763796	0.000000	
10	B	3.085598	3.143159	1.763439	1.809652	0.000000
11	B	1.695002	1.814676	2.916319	3.086615	3.548141

		11
11	B	0.000000

S43

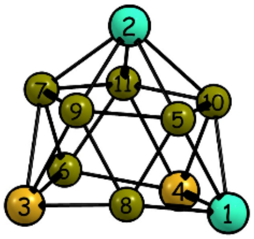

7. -8132.320876 +13.5 C_1

WBI: Co1-Co2: 0.0827

		1	2	3	4	5
1	Co	0.000000				
2	Co	3.666091	0.000000			
3	Se	3.516527	3.601310	0.000000		
4	Se	2.318167	3.604137	3.361800	0.000000	
5	B	2.058367	2.086527	3.207578	3.283609	0.000000
6	B	3.506795	3.282837	2.041809	2.036802	3.562275
7	B	3.959221	2.009125	2.155142	3.240036	2.972859
8	B	2.029328	3.291092	2.082768	3.275939	1.728367
9	B	3.306647	2.038029	2.156791	3.828792	1.758744
10	B	2.084192	2.058999	3.781246	2.156781	1.791419
11	B	3.437068	2.007639	3.238103	2.126684	2.990332

		6	7	8	9	10
6	B	0.000000				
7	B	1.819334	0.000000			
8	B	3.185876	3.080875	0.000000		
9	B	3.074909	1.923051	1.840366	0.000000	
10	B	3.075091	2.985474	2.916868	2.924583	0.000000
11	B	1.819618	1.757689	3.557120	2.973864	1.943872

		11
11	B	0.000000

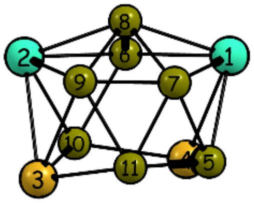

8. -8132.318964 +14.7 C_1

WBI: Co1-Co2: 0.0824

		1	2	3	4	5
1	Co	0.000000				
2	Co	3.729457	0.000000			
3	Se	4.192322	2.373522	0.000000		
4	Se	2.328931	3.642699	3.282320	0.000000	
5	B	2.153197	3.977320	3.116151	2.053522	0.000000
6	B	2.125583	2.042884	3.249150	2.229851	3.102166
7	B	2.091110	3.319723	3.193650	3.215379	1.795793
8	B	2.121383	2.096081	3.274629	3.307554	2.983593
9	B	3.312471	2.211475	2.223298	3.810993	2.917130
10	B	3.406897	2.070680	2.015806	2.057987	3.148434
11	B	3.306328	3.403763	2.040258	3.182617	1.747811

		6	7	8	9	10
6	B	0.000000				
7	B	2.922294	0.000000			
8	B	1.776352	1.782955	0.000000		
9	B	2.968692	1.710320	1.778518	0.000000	
10	B	1.879361	3.537866	2.991246	3.117952	0.000000
11	B	3.527222	1.736104	2.913130	1.807357	3.153760

		11
11	B	0.000000

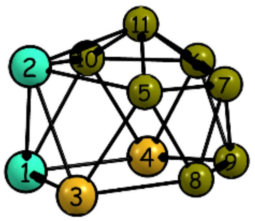

9. -8132.318011 +15.3 C_1

WBI: Co1-Co2: 0.4137

		1	2	3	4	5
1	Co	0.000000				
2	Co	2.531496	0.000000			
3	Se	2.316205	2.360167	0.000000		
4	Se	2.319072	3.752411	3.395992	0.000000	
5	B	3.602427	2.174288	2.179495	3.808507	0.000000
6	B	3.494993	3.388825	3.763137	2.082140	2.885805
7	B	4.038547	3.330065	3.193042	3.166548	1.720503
8	B	3.552836	3.422280	2.046623	3.179702	1.826205
9	B	3.537605	4.011879	3.164606	2.034421	2.897120
10	B	2.208532	2.118641	3.224024	2.169756	2.971006
11	B	3.445417	2.078429	3.228879	3.217628	1.788072

		6	7	8	9	10
6	B	0.000000				
7	B	1.755384	0.000000			
8	B	2.909152	1.749466	0.000000		
9	B	1.871047	1.749372	1.737322	0.000000	
10	B	1.889950	2.932802	3.510037	3.071280	0.000000
11	B	1.759570	1.789609	2.910923	2.922720	1.758009

		11
11	B	0.000000

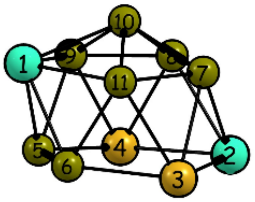

10. -8132.315674 +16.8 C_s

WBI: Co1-Co2: 0.0630

		1	2	3	4	5
1	Co	0.000000				
2	Co	4.466446	0.000000			
3	Se	3.585703	2.318892	0.000000		
4	Se	3.586784	2.319859	3.399431	0.000000	
5	B	2.072702	3.555362	3.170807	2.064099	0.000000
6	B	2.072335	3.553401	2.063617	3.168601	1.702495
7	B	3.320057	2.139588	2.111554	3.255906	3.537832
8	B	3.321431	2.138524	3.254669	2.111810	3.072492
9	B	2.020540	3.481177	3.786774	2.120518	1.919379
10	B	2.074455	3.338174	3.211227	3.211139	2.967524
11	B	2.018743	3.481450	2.120437	3.787404	2.935705

		6	7	8	9	10
6	B	0.000000				
7	B	3.071530	0.000000			
8	B	3.536379	1.805507	0.000000		
9	B	2.934652	2.956093	1.874099	0.000000	
10	B	2.967421	1.758170	1.758552	1.772128	0.000000
11	B	1.919587	1.873885	2.956963	2.896256	1.773521

		11
11	B	0.000000

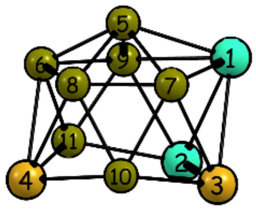

11. -8132.313545 +18.1 C_1

WBI: Co1-Co2: 0.3927

		1	2	3	4	5
1	Co	0.000000				
2	Co	2.484420	0.000000			
3	Se	2.360219	2.318332	0.000000		
4	Se	4.228942	3.476739	3.348525	0.000000	
5	B	2.107392	3.418180	3.275421	3.226192	0.000000
6	B	3.319900	3.341100	3.810653	2.155213	1.748631
7	B	2.148699	3.533947	2.196789	3.233120	1.754707
8	B	3.320816	3.991583	3.247201	2.162675	1.744107
9	B	2.120583	2.122993	3.248063	3.190188	1.820119
10	B	3.398613	3.507487	2.040417	2.045077	2.918078
11	B	3.379500	2.050528	3.292201	2.056955	2.914500

		6	7	8	9	10
6	B	0.000000				
7	B	2.921730	0.000000			
8	B	1.887523	1.716557	0.000000		
9	B	1.757182	2.956629	2.941211	0.000000	
10	B	3.046904	1.818676	1.813591	3.525415	0.000000
11	B	1.815548	3.564096	3.055377	1.728969	3.187785

		11
11	B	0.000000

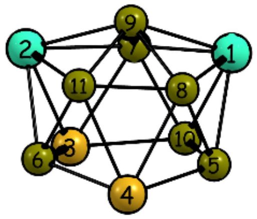

12. -8132.304152 +24.0 C_1

WBI: Co1-Co2: 0.0828

		1	2	3	4	5
1	Co	0.000000				
2	Co	3.699548	0.000000			
3	Se	3.606371	2.351372	0.000000		
4	Se	3.592292	3.616994	3.309690	0.000000	
5	B	2.075463	3.994220	3.126794	2.095889	0.000000
6	B	4.008595	2.084962	2.018226	2.044822	3.163619
7	B	2.001671	2.182714	2.185236	3.787024	2.922518
8	B	2.022223	3.352507	3.763212	2.126807	1.948024
9	B	2.051724	2.096372	3.266933	3.238798	2.995290
10	B	2.069472	3.475463	2.084739	3.169217	1.674685
11	B	3.297669	2.076802	3.247260	2.140833	3.106967

		6	7	8	9	10
6	B	0.000000				
7	B	3.100159	0.000000			
8	B	3.067480	2.901926	0.000000		
9	B	2.981349	1.803560	1.773966	0.000000	
10	B	3.169747	1.910056	2.936003	2.990129	0.000000
11	B	1.883648	2.964973	1.853695	1.756022	3.553379

		11
11	B	0.000000

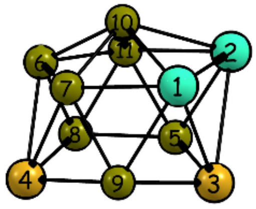

13. -8132.303052 +24.7 C_1

WBI: Co1-Co2: 0.3055

		1	2	3	4	5
1	Co	0.000000				
2	Co	2.609317	0.000000			
3	Se	2.471638	2.412928	0.000000		
4	Se	3.624750	4.263674	3.306162	0.000000	
5	B	3.563205	2.095237	2.052783	3.157205	0.000000
6	B	3.364168	3.341533	3.758764	2.119601	2.930588
7	B	2.024876	3.442038	3.263252	2.163600	3.549777
8	B	4.030990	3.292678	3.163710	2.039654	1.755112
9	B	2.065421	3.517538	2.020341	2.057813	3.148278
10	B	2.109520	2.156261	3.252479	3.250489	2.960912
11	B	3.420876	2.050627	3.206358	3.177371	1.784464

		6	7	8	9	10
6	B	0.000000				
7	B	1.856295	0.000000			
8	B	1.836464	3.046377	0.000000		
9	B	3.054844	1.882346	3.151920	0.000000	
10	B	1.793845	1.766949	2.929708	2.962009	0.000000
11	B	1.753533	2.921384	1.736205	3.536772	1.802739

		11
11	B	0.000000

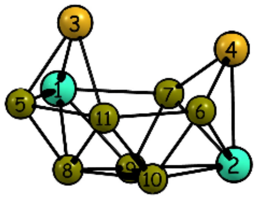

14. -8132.300931 +26.0 C_1

WBI: Co1-Co2: 0.0984

		1	2	3	4	5
1	Co	0.000000				
2	Co	3.851061	0.000000			
3	Se	2.400878	4.428610	0.000000		
4	Se	3.649616	2.308312	3.320698	0.000000	
5	B	2.117361	4.589335	1.932137	4.442612	0.000000
6	B	3.725584	2.150879	3.063307	2.004090	3.594083
7	B	2.229389	2.159864	3.313246	2.003245	3.754809
8	B	2.149211	3.382574	2.969053	4.061769	1.697497
9	B	2.144927	2.099143	3.583880	3.187279	3.102499
10	B	3.203559	2.062121	3.449978	3.163907	3.005234
11	B	2.978577	3.474310	2.119873	3.422201	1.860102

		6	7	8	9	10
6	B	0.000000				
7	B	2.849809	0.000000			
8	B	3.037936	3.076271	0.000000		
9	B	2.854469	1.744776	1.765692	0.000000	
10	B	1.724513	2.839960	1.750901	1.789063	0.000000
11	B	1.962107	3.434894	1.848660	2.846545	1.780002

		11
11	B	0.000000

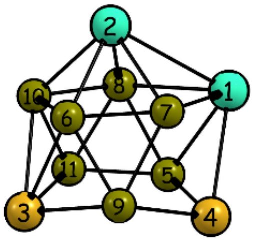

15. -8132.295584 +29.4 C_1

WBI: Co1-Co2: 0.3426

		1	2	3	4	5
1	Co	0.000000				
2	Co	2.545237	0.000000			
3	Se	4.233370	3.609532	0.000000		
4	Se	2.332139	3.795752	3.278657	0.000000	
5	B	2.116033	3.439949	3.146594	2.041362	0.000000
6	B	3.411273	2.003367	2.155311	3.274633	3.550084
7	B	2.162326	2.022635	3.267750	2.310297	3.109966
8	B	2.117301	2.083112	3.206860	3.222022	1.807285
9	B	3.366962	3.306436	2.050620	2.024572	3.125944
10	B	3.430156	2.036311	2.150247	3.821432	2.943037
11	B	3.313681	3.290241	2.063109	3.161034	1.749996

		6	7	8	9	10
6	B	0.000000				
7	B	1.747620	0.000000			
8	B	2.930571	2.879538	0.000000		
9	B	1.792787	1.853260	3.489732	0.000000	
10	B	1.952705	2.972319	1.769079	3.071050	0.000000
11	B	3.044994	3.523669	1.745577	3.147558	1.788982

		11
11	B	0.000000

Table S5A: Initial 12-vertex $[BH]_{12}^{2-}$ polyhedra upon which the starting structures are based; the H atoms are omitted for clarity.

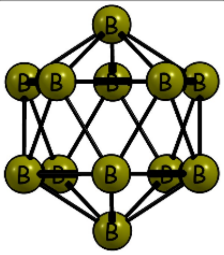 1. Icosahedron	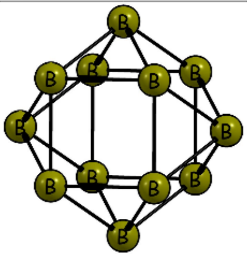 2. Cube octahedron	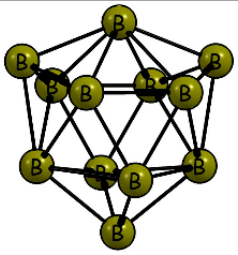 3. Anticube octahedron
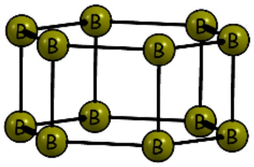 4. Hexagonal prism	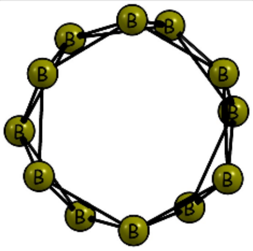 5. Hexagonal antiprism	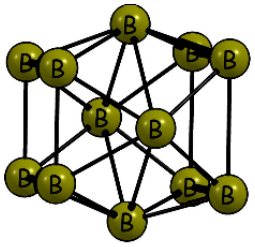 6. Double square antiprism
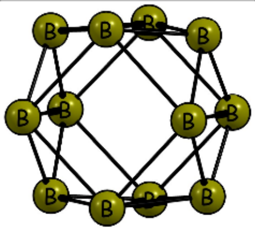 7. Tetracapped cube A	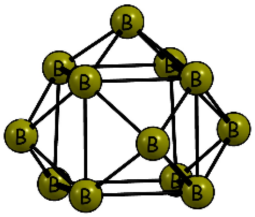 8. Tetracapped cube B	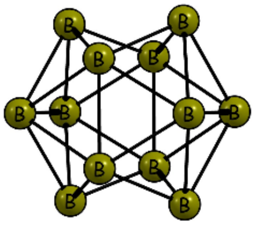 9. 2v6 structure

Table S5B: Distance table for the lowest-lying $Cp_2Co_2S_2B_8H_8$ optimized structures obtained at the PBE0/def2-TZVP level of theory. Included are the zero-point corrected absolute energy in (a.u.) at the DLPNO-CCSD(T)/def2-QZVP level of theory with zero-point energy obtained from the PBE0/def2-TZVP computations, relative energy in (kcal/mol), symmetry and Wiberg bond indecies. For clarity, only the atoms forming the cluster framework are shown.

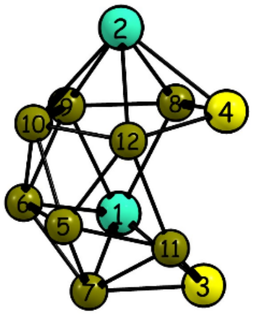

1. -4151.338626 0.0 C_1

WBI: Co1-Co2: 0.0810

		1	2	3	4	5
1	Co	0.000000				
2	Co	3.840028	0.000000			
3	S	2.225380	4.479199	0.000000		
4	S	3.499721	2.160777	3.078431	0.000000	
5	B	3.219732	3.371849	3.012128	3.227898	0.000000
6	B	2.121242	3.350316	3.182111	3.729784	1.787210
7	B	2.169979	4.320265	1.880179	3.730522	1.785537
8	B	2.192889	2.134181	3.004105	1.868030	3.462185
9	B	2.146406	2.114066	3.647316	3.025978	2.875788
10	B	3.298162	2.088735	3.986968	3.093309	1.767908
11	B	3.172771	3.671143	1.866587	2.409873	1.750702
12	B	3.757930	2.153406	3.445713	1.923146	1.772586

		6	7	8	9	10
6	B	0.000000				
7	B	1.785875	0.000000			
8	B	2.979118	3.436057	0.000000		
9	B	1.777044	3.095911	1.753451	0.000000	
10	B	1.733364	2.983303	2.889772	1.782511	0.000000
11	B	2.910252	1.970812	3.158702	3.496939	2.991297
12	B	2.867966	3.111453	2.855563	2.852734	1.803988

		11	12
11	B	0.000000	
12	B	1.929115	0.000000

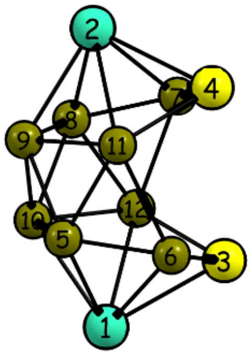

2. -4151.333782 +3.0 C_2

WBI: Co1-Co2: 0.0570

		1	2	3	4	5
1	Co	0.000000				
2	Co	4.808369	0.000000			
3	S	2.157928	4.334852	0.000000		
4	S	4.334044	2.158058	3.083633	0.000000	
5	B	2.113602	3.424547	3.018397	3.356616	0.000000
6	B	2.152373	3.873144	1.851878	2.741137	1.734927
7	B	3.873237	2.151889	2.742699	1.852646	3.484010
8	B	3.423060	2.114229	3.356650	3.018282	2.866285
9	B	3.360353	2.075813	3.846958	3.107278	1.772400
10	B	2.075491	3.360314	3.107854	3.846169	1.794632
11	B	3.512004	2.249156	3.287684	1.901916	1.800879
12	B	2.248003	3.512438	1.902070	3.287432	2.847941

		6	7	8	9	10
6	B	0.000000				
7	B	3.233468	0.000000			
8	B	3.482907	1.734607	0.000000		
9	B	3.032101	2.882354	1.794805	0.000000	
10	B	2.882198	3.032059	1.771439	1.725881	0.000000
11	B	2.002350	2.796125	2.848007	1.857917	2.895777
12	B	2.795156	2.002926	1.800608	2.896355	1.858139

		11	12
11	B	0.000000	
12	B	3.302128	0.000000

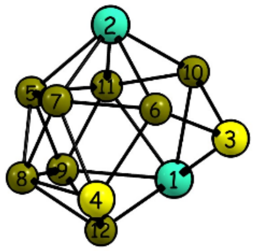

3. -4151.333610 +3.1 C_1

WBI: Co1-Co2: 0.0741

		1	2	3	4	5
1	Co	0.000000				
2	Co	3.589186	0.000000			
3	S	2.231609	3.095034	0.000000		
4	S	3.466219	3.545481	3.053778	0.000000	
5	B	3.312039	2.062693	3.897344	3.105517	0.000000
6	B	3.255250	2.150304	1.897406	2.021368	3.000600
7	B	3.823320	2.007540	3.463971	2.019511	1.750266
8	B	3.374761	3.304598	3.969022	1.948442	1.766216
9	B	2.112035	3.247061	3.577604	3.026046	1.782895
10	B	2.147343	2.100732	1.840231	3.885965	3.089872
11	B	2.159178	2.078530	3.089953	3.757098	1.762716
12	B	2.114225	3.872802	3.016750	1.916796	2.894804

		6	7	8	9	10
6	B	0.000000				
7	B	1.864314	0.000000			
8	B	3.026489	1.838993	0.000000		
9	B	3.438400	2.853147	1.773103	0.000000	
10	B	2.512802	3.360934	3.945456	3.094090	0.000000
11	B	3.100392	2.900740	2.939427	1.781790	1.764459
12	B	2.986206	2.945952	1.842120	1.721660	3.493500

		11	12
11	B	0.000000	
12	B	2.983714	0.000000

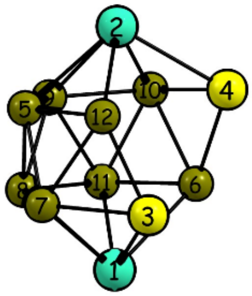

4. -4151.332977 +3.5 C_2

WBI: Co1-Co2: 0.0375

		1	2	3	4	5
1	Co	0.000000				
2	Co	4.156738	0.000000			
3	S	2.205041	3.618912	0.000000		
4	S	3.618997	2.205090	3.232260	0.000000	
5	B	3.234683	2.138003	3.002789	3.560663	0.000000
6	B	2.193168	3.227129	2.943684	1.852224	3.571547
7	B	2.165439	3.458690	1.887079	4.000621	1.782513
8	B	2.103426	3.284222	3.152295	3.881146	1.790246
9	B	3.283920	2.104936	3.882044	3.152366	1.766443
10	B	3.459256	2.166543	4.001235	1.886765	3.099929
11	B	2.138700	3.235684	3.561647	3.002628	2.904581
12	B	3.226126	2.192081	1.851916	2.942390	1.732718

		6	7	8	9	10
6	B	0.000000				
7	B	3.475390	0.000000			
8	B	2.988789	1.808431	0.000000		
9	B	2.907424	2.972882	1.736410	0.000000	
10	B	1.885809	3.927579	2.972846	1.808756	0.000000
11	B	1.732461	3.100256	1.766275	1.790024	1.782690
12	B	3.374780	1.885947	2.907354	2.989384	3.474949

		11	12
11	B	0.000000	
12	B	3.571597	0.000000

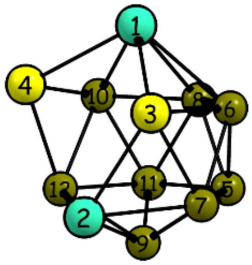

5. -4151.332531 +3.8 C_1

WBI: Co1-Co2: 0.0995

		1	2	3	4	5
1	Co	0.000000				
2	Co	3.706370	0.000000			
3	S	2.301181	2.181055	0.000000		
4	S	2.238380	3.116750	3.133321	0.000000	
5	B	3.336126	3.270167	3.061765	3.969654	0.000000
6	B	2.207411	3.325979	1.948752	3.683587	1.750232
7	B	3.459292	2.081365	1.963790	3.985679	1.768107
8	B	2.163948	3.923984	3.176802	3.151814	1.751675
9	B	3.803483	2.023805	3.057565	3.437607	1.789359
10	B	2.287792	3.807998	3.653494	1.844950	2.993760
11	B	3.283595	3.372087	3.698809	3.041530	1.748690
12	B	3.234831	2.214533	3.227022	1.938820	2.920507

		6	7	8	9	10
6	B	0.000000				
7	B	1.865951	0.000000			
8	B	1.778941	2.957084	0.000000		
9	B	2.876979	1.793722	2.859068	0.000000	
10	B	3.160690	3.824994	1.818706	3.058775	0.000000
11	B	2.867516	2.929835	1.752526	1.776137	1.764352
12	B	3.463987	3.030845	2.929855	1.774978	2.039669

		11	12
11	B	0.000000	
12	B	1.844946	0.000000

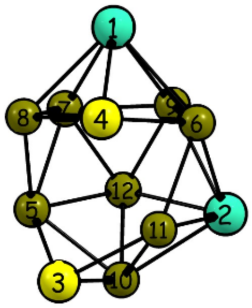

6. -4151.329636 +5.6 C_1

WBI: Co1-Co2: 0.0854

		1	2	3	4	5
1	Co	0.000000				
2	Co	3.710429	0.000000			
3	S	4.324056	3.252086	0.000000		
4	S	2.177995	3.549234	3.000368	0.000000	
5	B	3.456143	3.212868	1.936645	3.196505	0.000000
6	B	2.136738	2.023391	3.481048	1.964226	3.392995
7	B	2.097052	3.228464	3.399032	3.015294	1.794794
8	B	2.103915	3.857208	2.799682	1.883701	1.952995
9	B	2.102850	2.063213	3.903058	3.136893	2.915824
10	B	4.333391	2.060742	1.876702	3.799715	1.887843
11	B	3.528475	2.050948	1.901662	2.184358	2.819253
12	B	3.365853	2.083609	3.065991	3.772541	1.832326

		6	7	8	9	10
6	B	0.000000				
7	B	2.833327	0.000000			
8	B	2.877406	1.719854	0.000000		
9	B	1.806072	1.784586	2.903114	0.000000	
10	B	3.262618	2.998887	3.409032	3.023851	0.000000
11	B	1.928248	3.373436	2.981487	3.007222	2.221965
12	B	2.914935	1.771030	2.996518	1.758808	1.727378

		11	12
11	B	0.000000	
12	B	2.975981	0.000000

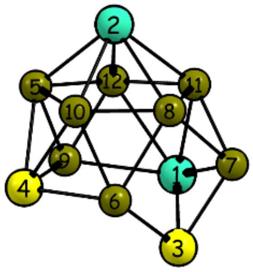

7. -4151.327331 +7.1 C_1

WBI: Co1-Co2: 0.0810

		1	2	3	4	5
1	Co	0.000000				
2	Co	3.708475	0.000000			
3	S	2.235602	4.320442	0.000000		
4	S	3.465654	3.461033	3.071718	0.000000	
5	B	3.381259	2.011450	4.013997	1.990241	0.000000
6	B	3.221719	3.363913	1.886621	1.967576	3.035459
7	B	2.131450	3.404127	1.836856	3.878332	3.948585
8	B	3.196113	2.064182	2.805921	3.203604	2.983392
9	B	2.117828	3.265274	3.052159	1.928240	1.829289
10	B	3.789087	1.989139	3.417627	2.023449	1.912185
11	B	2.156303	2.032582	3.076034	3.748964	2.940840
12	B	2.145292	2.078709	3.622823	3.049383	1.755691

		6	7	8	9	10
6	B	0.000000				
7	B	2.525697	0.000000			
8	B	1.947067	1.884458	0.000000		
9	B	2.943824	3.481118	3.435543	0.000000	
10	B	1.809603	3.294349	1.746824	2.938468	0.000000
11	B	3.085915	1.728955	1.776070	2.968370	2.859081
12	B	3.436660	3.098162	2.870987	1.723567	2.862836

		11	12
11	B	0.000000	
12	B	1.796887	0.000000

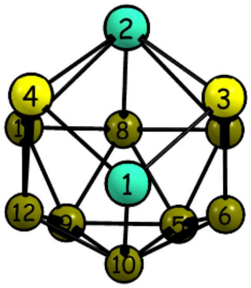

8. -4151.326013 +7.9 C_{2v}

WBI: Co1-Co2: 0.1087

		1	2	3	4	5
1	Co	0.000000				
2	Co	3.319937	0.000000			
3	S	2.265168	2.265726	0.000000		
4	S	2.262516	2.265333	3.049748	0.000000	
5	B	3.375194	3.373445	3.087780	3.863400	0.000000
6	B	2.343088	3.385765	1.984198	3.687637	1.694922
7	B	3.387616	2.343525	1.984926	3.690292	1.694951
8	B	3.775481	2.169576	3.171599	3.168140	1.806903
9	B	3.378317	3.375473	3.870964	3.085234	1.780011
10	B	2.170387	3.770871	3.170287	3.162459	1.806588
11	B	3.390733	2.344978	3.695698	1.985097	2.920039
12	B	2.350522	3.388068	3.697614	1.981773	2.921494
		6	7	8	9	10
6	B	0.000000				
7	B	1.800814	0.000000			
8	B	2.891318	1.786136	0.000000		
9	B	2.921818	2.922262	1.807739	0.000000	
10	B	1.785921	2.890772	2.869949	1.806157	0.000000
11	B	3.653897	3.179920	1.785008	1.694935	2.888824
12	B	3.181668	3.656106	2.891429	1.694870	1.785278
		11	12			
11	B	0.000000				
12	B	1.800067	0.000000			

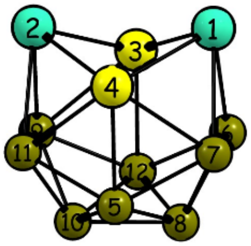

9. -4151.324329 +9.0 C_{2v}

WBI: Co1-Co2: 0.1721

		1	2	3	4	5
1	Co	0.000000				
2	Co	2.958690	0.000000			
3	S	2.230032	2.235218	0.000000		
4	S	2.235390	2.230077	3.062412	0.000000	
5	B	3.375826	3.370077	3.576032	2.000409	0.000000
6	B	2.092368	3.700592	2.102750	3.122944	2.895409
7	B	2.095680	3.694809	3.122503	2.097185	1.836003
8	B	3.277281	4.000327	3.106175	3.105380	1.777020
9	B	3.695694	2.096334	2.097372	3.123757	2.893797
10	B	4.000987	3.277284	3.105335	3.106948	1.776642
11	B	3.701617	2.092268	3.122808	2.104208	1.837375
12	B	3.370987	3.375860	2.000524	3.576923	2.870070
		6	7	8	9	10
6	B	0.000000				
7	B	1.744231	0.000000			
8	B	1.723864	1.723704	0.000000		
9	B	3.139916	3.589623	2.924513	0.000000	
10	B	2.926519	2.924804	1.779408	1.723718	0.000000
11	B	3.594709	3.140626	2.926621	1.744245	1.723811
12	B	1.837199	2.893948	1.776706	1.835613	1.777077
		11	12			
11	B	0.000000				
12	B	2.895436	0.000000			

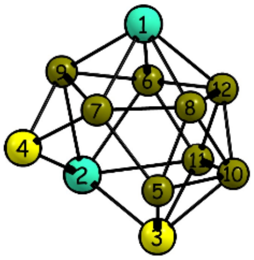

10. -4151.323914 +9.2 C_1

WBI: Co1-Co2: 0.0799

		1	2	3	4	5
1	Co	0.000000				
2	Co	3.652011	0.000000			
3	S	4.079585	2.299273	0.000000		
4	S	3.273197	2.231727	3.088295	0.000000	
5	B	3.342193	3.360637	1.870996	2.916017	0.000000
6	B	2.042388	2.196161	3.191339	3.156763	3.511825
7	B	2.118244	3.232784	3.103387	1.927166	1.907793
8	B	2.058261	3.808117	2.980664	3.433227	1.699067
9	B	2.034727	2.231664	3.641654	1.844948	3.545432
10	B	3.305728	3.478791	1.962224	4.008950	1.797718
11	B	3.234885	2.229132	1.976475	3.675105	2.906953
12	B	2.050698	3.363209	3.060788	3.979168	2.880012

		6	7	8	9	10
6	B	0.000000				
7	B	2.984602	0.000000			
8	B	2.891438	1.767584	0.000000		
9	B	1.817694	2.225614	3.196515	0.000000	
10	B	2.980545	3.014472	1.782462	3.912429	0.000000
11	B	1.782035	3.434928	2.844510	3.157258	1.844810
12	B	1.768580	2.951939	1.813123	3.074260	1.771216

		11	12
11	B	0.000000	
12	B	1.728624	0.000000

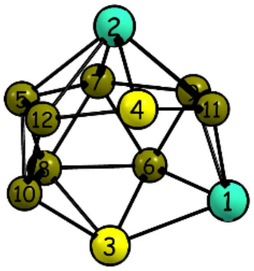

11. -4151.323142 +9.7 C_1

WBI: Co1-Co2: 0.0912

		1	2	3	4	5
1	Co	0.000000				
2	Co	3.715768	0.000000			
3	S	2.192828	3.931761	0.000000		
4	S	3.405306	2.257804	3.609809	0.000000	
5	B	3.991938	2.183822	2.978938	3.189161	0.000000
6	B	2.048707	3.375270	2.019432	4.097944	2.917488
7	B	3.246389	2.172809	3.011198	3.722566	1.774082
8	B	3.412877	3.367020	2.008138	4.115293	1.722266
9	B	2.038571	2.223234	3.053735	3.196822	3.027074
10	B	3.582517	3.299571	1.881532	3.131291	1.717470
11	B	2.054273	2.250697	3.260826	1.830008	3.501190
12	B	3.953070	2.244310	3.146629	1.812056	1.797251

		6	7	8	9	10
6	B	0.000000				
7	B	1.752695	0.000000			
8	B	1.861711	1.769871	0.000000		
9	B	1.757057	1.770998	2.968391	0.000000	
10	B	2.998820	2.854049	1.769313	3.576698	0.000000
11	B	3.043547	3.092480	3.871217	1.787452	3.619174
12	B	3.804677	3.099496	3.015006	3.497989	1.793466

		11	12
11	B	0.000000	
12	B	2.985122	0.000000

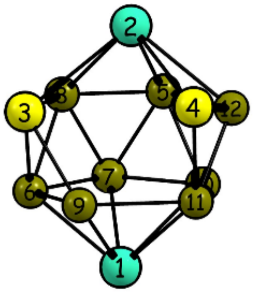

12. -4151.319236 +12.2 C_1

WBI: Co1-Co2: 0.0420

		1	2	3	4	5
1	Co	0.000000				
2	Co	4.243424	0.000000			
3	S	3.335797	2.368923	0.000000		
4	S	3.741248	2.245175	3.086889	0.000000	
5	B	3.263643	2.155375	3.167816	3.194055	0.000000
6	B	2.053551	3.506746	1.954891	4.008432	2.942402
7	B	2.082603	3.383636	3.061542	4.002435	1.775677
8	B	3.235753	2.286597	1.992217	3.717901	1.758926
9	B	1.944306	3.279626	1.889114	2.687536	3.422649
10	B	2.100967	3.309771	3.722235	3.095138	1.778018
11	B	2.074754	3.244246	3.314789	1.925676	2.891929
12	B	3.368393	2.265666	3.614916	1.887007	1.825679
		6	7	8	9	10
6	B	0.000000				
7	B	1.748446	0.000000			
8	B	1.816269	1.729778	0.000000		
9	B	1.945352	2.894329	2.932457	0.000000	
10	B	2.991341	1.784864	2.885306	2.977851	0.000000
11	B	3.193213	2.948217	3.515131	2.021784	1.787284
12	B	3.809727	2.985364	3.153060	3.270153	1.733598
		11	12			
11	B	0.000000				
12	B	1.874099	0.000000			

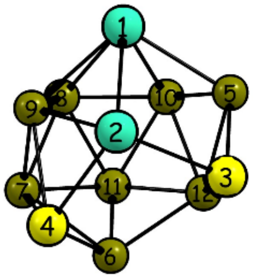

13. -4151.315815 +14.3 C_1

WBI: Co1-Co2: 0.3850

		1	2	3	4	5
1	Co	0.000000				
2	Co	2.615725	0.000000			
3	S	3.195878	2.210016	0.000000		
4	S	3.663997	2.216393	3.096989	0.000000	
5	B	2.117240	2.939333	1.830215	3.925170	0.000000
6	B	3.892076	3.289406	2.827704	1.888023	3.408259
7	B	3.353606	3.413538	3.909796	1.940310	3.910758
8	B	2.077715	3.463688	3.839778	3.097896	3.048623
9	B	2.077560	2.190874	3.512985	2.055966	3.373516
10	B	2.082295	3.635001	3.022878	3.800036	1.721558
11	B	3.255261	3.889962	3.398755	2.996714	3.001889
12	B	3.229302	3.363915	1.940805	3.205304	1.877524
		6	7	8	9	10
6	B	0.000000				
7	B	1.863683	0.000000			
8	B	2.872350	1.742638	0.000000		
9	B	2.948483	1.859266	1.749824	0.000000	
10	B	2.970725	2.940355	1.765264	2.932614	0.000000
11	B	1.694975	1.766251	1.783433	2.861534	1.769436
12	B	1.899603	3.031081	2.898740	3.434422	1.840102
		11	12			
11	B	0.000000				
12	B	1.786664	0.000000			

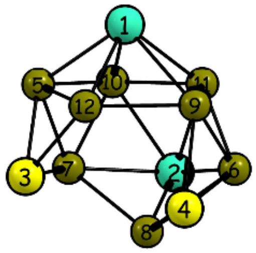

14. -4151.314167 +15.3 C_1

WBI: Co1-Co2: 0.0691

		1	2	3	4	5
1	Co	0.000000				
2	Co	3.648894	0.000000			
3	S	3.323440	3.673341	0.000000		
4	S	3.671299	3.320836	3.063058	0.000000	
5	B	2.049840	3.363882	1.916059	3.887887	0.000000
6	B	3.361778	2.049506	3.891654	1.917022	3.915076
7	B	3.223545	2.069250	1.927086	3.391415	1.842753
8	B	3.878458	1.953632	2.603069	1.884194	3.348595
9	B	2.067718	3.222394	3.393867	1.926859	3.203661
10	B	2.085823	2.076616	3.080076	3.853817	1.747616
11	B	2.075975	2.086055	3.858057	3.081898	3.025603
12	B	1.953726	3.879115	1.884738	2.604050	1.974029

		6	7	8	9	10
6	B	0.000000				
7	B	3.205421	0.000000			
8	B	1.979124	2.028762	0.000000		
9	B	1.841321	3.422327	2.848666	0.000000	
10	B	3.024543	1.792713	3.018340	2.933664	0.000000
11	B	1.748255	2.935569	2.909169	1.794558	1.801263
12	B	3.350903	2.847849	3.101587	2.031399	2.905894

		11	12
11	B	0.000000	
12	B	3.021558	0.000000

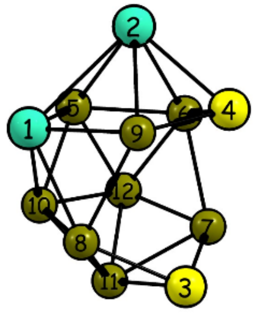

15. -4151.306961 +19.9 C_2

WBI: Co1-Co2: 0.3452

		1	2	3	4	5
1	Co	0.000000				
2	Co	2.538670	0.000000			
3	S	3.741918	4.456303	0.000000		
4	S	3.369367	2.154584	3.055331	0.000000	
5	B	2.090422	2.100466	3.880679	3.089476	0.000000
6	B	3.296914	2.154094	3.343796	1.903605	1.865558
7	B	3.903313	3.780118	1.871974	2.573639	3.018858
8	B	2.095892	3.763905	1.915172	3.323081	2.924390
9	B	1.946132	2.251228	2.895020	1.848596	2.866681
10	B	2.060974	3.512904	3.070574	3.800403	1.769893
11	B	3.385255	4.422279	1.906048	3.809122	2.982006
12	B	3.267043	3.402857	3.005702	3.279569	1.793661

		6	7	8	9	10
6	B	0.000000				
7	B	1.944094	0.000000			
8	B	3.370903	2.798256	0.000000		
9	B	2.842019	3.248701	2.194177	0.000000	
10	B	2.904330	2.861440	1.794358	3.077424	0.000000
11	B	3.084759	1.885524	1.873702	3.529060	1.739038
12	B	1.774789	1.727814	2.819078	3.509851	1.779464

		11	12
11	B	0.000000	
12	B	1.776066	0.000000

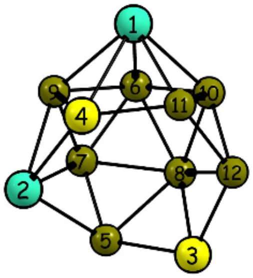

16. -4151.303442 +22.1 C_1

WBI: Co1-Co2: 0.1019

		1	2	3	4	5
1	Co	0.000000				
2	Co	3.759773	0.000000			
3	S	4.516658	3.430251	0.000000		
4	S	2.268861	2.200564	3.648929	0.000000	
5	B	4.265394	2.031715	1.834311	3.244832	0.000000
6	B	2.071552	3.251894	3.639119	3.031166	3.034340
7	B	3.335702	2.056502	3.147812	3.073784	1.745283
8	B	3.394983	3.321007	2.071708	3.507859	1.986373
9	B	2.143031	2.039955	4.084027	2.055631	2.990187
10	B	2.035665	3.977015	3.094759	2.995135	3.433966
11	B	2.028572	3.570879	3.142194	1.864511	3.595572
12	B	3.373920	3.974737	1.792047	3.182153	2.984776

		6	7	8	9	10
6	B	0.000000				
7	B	1.755225	0.000000			
8	B	1.842990	1.883470	0.000000		
9	B	1.776196	1.743276	2.959668	0.000000	
10	B	1.750213	2.990587	1.915846	2.937805	0.000000
11	B	2.859804	3.576044	2.979388	3.022636	1.753424
12	B	3.070579	3.491117	2.077507	3.831743	1.755647

		11	12
11	B	0.000000	
12	B	1.831436	0.000000

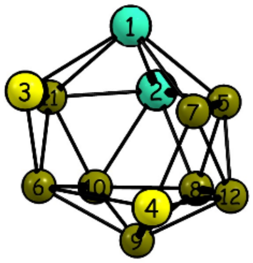

17. -4151.300089 +24.2 C_1

WBI: Co1-Co2: 0.3552

		1	2	3	4	5
1	Co	0.000000				
2	Co	2.541363	0.000000			
3	S	2.219708	3.582020	0.000000		
4	S	3.522253	4.266429	3.158380	0.000000	
5	B	2.160632	2.087557	3.618097	3.007303	0.000000
6	B	3.227317	3.322723	1.835960	2.656929	3.553562
7	B	2.162695	3.442227	3.014147	1.861674	1.736407
8	B	3.377729	2.017949	3.948426	3.058619	1.828506
9	B	3.754093	3.281225	3.353694	1.912055	2.858257
10	B	3.314680	2.088123	2.994168	3.327809	2.917209
11	B	2.304726	2.082448	1.896791	3.865876	3.147550
12	B	3.445997	3.329754	4.033904	1.895613	1.798499

		6	7	8	9	10
6	B	0.000000				
7	B	3.287256	0.000000			
8	B	2.995961	2.893162	0.000000		
9	B	1.956598	2.839127	1.784410	0.000000	
10	B	1.720329	3.518240	1.779266	1.799106	0.000000
11	B	1.910110	3.520029	3.019168	3.107380	1.786355
12	B	3.380215	1.851606	1.760690	1.881615	3.017324

		11	12
11	B	0.000000	
12	B	3.918912	0.000000

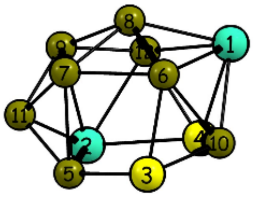

18. -4151.299478 +24.6 C_1

WBI: Co1-Co2: 0.0858

		1	2	3	4	5
1	Co	0.000000				
2	Co	3.771769	0.000000			
3	S	3.487133	3.559033	0.000000		
4	S	2.244737	2.261579	3.154642	0.000000	
5	B	4.082615	2.300067	1.875938	3.197492	0.000000
6	B	2.139783	3.740874	1.965635	3.076543	2.811503
7	B	3.455466	3.044199	2.630063	3.694254	2.015099
8	B	2.047966	3.204082	3.318738	3.063687	3.210463
9	B	3.377639	2.156264	3.870451	3.274198	2.811590
10	B	2.063757	3.426588	1.863970	1.871120	2.983376
11	B	4.334794	2.065157	3.284129	3.707131	1.650234
12	B	2.114818	2.176708	3.874953	2.014582	3.408398

		6	7	8	9	10
6	B	0.000000				
7	B	1.980844	0.000000			
8	B	1.750513	1.779678	0.000000		
9	B	3.083334	1.873068	1.779804	0.000000	
10	B	1.933273	3.332702	2.974010	3.865387	0.000000
11	B	3.340816	1.679607	2.852029	1.675730	3.996099
12	B	2.935040	2.888402	1.776903	1.741399	3.036553

		11	12
11	B	0.000000	
12	B	3.000963	0.000000

Table S5C: Distance table for the lowest-lying $Cp_2Co_2Se_2B_8H_8$ optimized structures obtained at the PBE0/def2-TZVP level of theory. Included are the zero-point corrected absolute energy in (a.u.) at the DLPNO-CCSD(T)/def2-QZVP level of theory with zero-point energy obtained from the PBE0/def2-TZVP computations, relative energy in (kcal/mol), symmetry and Wiberg bond indecies. For clarity, only the atoms forming the cluster framework are shown.

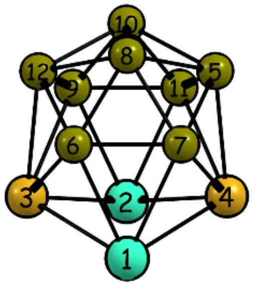

1. -8157.745753 0.0 C_{2v}

WBI: Co1-Co2: 0.1749

		1	2	3	4	5
1	Co	0.000000				
2	Co	3.176512	0.000000			
3	Se	2.339641	2.344674	0.000000		
4	Se	2.344162	2.340028	3.248172	0.000000	
5	B	3.444857	3.441399	3.725458	2.135123	0.000000
6	B	2.121133	3.834470	2.239033	3.263151	2.907521
7	B	2.124760	3.832231	3.263812	2.237583	1.862437
8	B	3.313706	4.080323	3.249655	3.249206	1.774549
9	B	3.830741	2.124739	2.236284	3.263680	2.906806
10	B	4.079485	3.313800	3.248692	3.249631	1.774267
11	B	3.833252	2.120613	3.262446	2.238664	1.862364
12	B	3.440997	3.445795	2.135065	3.725920	2.869964

		6	7	8	9	10
6	B	0.000000				
7	B	1.736450	0.000000			
8	B	1.719691	1.719211	0.000000		
9	B	3.205419	3.645367	2.944687	0.000000	
10	B	2.945256	2.944878	1.783303	1.719277	0.000000
11	B	3.646442	3.206078	2.945520	1.736759	1.719852
12	B	1.861982	2.906729	1.774241	1.862251	1.774706

		11	12
11	B	0.000000	
12	B	2.907874	0.000000

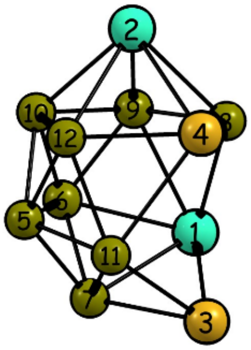

2. -8157.739425 +4.0 C_1

WBI: Co1-Co2: 0.1158

		1	2	3	4	5
1	Co	0.000000				
2	Co	3.850401	0.000000			
3	Se	2.366432	4.625036	0.000000		
4	Se	3.609751	2.287018	3.176531	0.000000	
5	B	3.250243	3.358225	3.194987	3.304305	0.000000
6	B	2.121160	3.344356	3.345365	3.835296	1.784715
7	B	2.186490	4.290453	2.018580	3.763695	1.780120
8	B	2.196349	2.151510	3.147270	2.024337	3.497353
9	B	2.145688	2.114725	3.808086	3.164630	2.875094
10	B	3.316720	2.087304	4.182754	3.234909	1.771926
11	B	3.247247	3.601044	2.057596	2.339749	1.772076
12	B	3.842958	2.153551	3.687368	2.092208	1.753655

		6	7	8	9	10
6	B	0.000000				
7	B	1.768628	0.000000			
8	B	2.981215	3.437682	0.000000		
9	B	1.776492	3.079204	1.742128	0.000000	
10	B	1.736372	2.974979	2.917486	1.782719	0.000000
11	B	2.933462	2.030579	3.178327	3.489410	2.984844
12	B	2.866517	3.124363	2.971964	2.874630	1.777799

		11	12
11	B	0.000000	
12	B	1.925827	0.000000

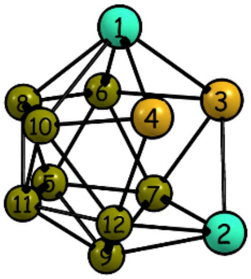

3. -8157.738481 +4.5 C_1

WBI: Co1-Co2: 0.0936

		1	2	3	4	5
1	Co	0.000000				
2	Co	3.832194	0.000000			
3	Se	2.421855	2.307901	0.000000		
4	Se	2.383339	3.212893	3.273044	0.000000	
5	B	3.363131	3.285603	3.202538	4.151812	0.000000
6	B	2.240893	3.399110	2.088934	3.856648	1.744906
7	B	3.530266	2.113536	2.097937	4.138417	1.761631
8	B	2.156269	3.955524	3.305888	3.330914	1.757130
9	B	3.870490	2.019830	3.202420	3.606214	1.787928
10	B	2.286278	3.797625	3.741566	2.004368	2.972644
11	B	3.311407	3.372269	3.842376	3.226469	1.750982
12	B	3.319795	2.234573	3.378409	2.102540	2.919393

		6	7	8	9	10
6	B	0.000000				
7	B	1.896345	0.000000			
8	B	1.772563	2.966774	0.000000		
9	B	2.897788	1.788934	2.868419	0.000000	
10	B	3.145469	3.803724	1.803838	3.042215	0.000000
11	B	2.872051	2.927781	1.757312	1.777135	1.748495
12	B	3.502110	3.050670	2.934503	1.780872	2.017310

		11	12
11	B	0.000000	
12	B	1.822431	0.000000

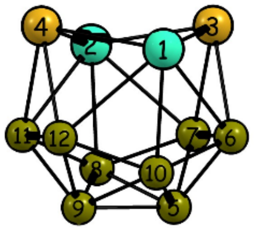

4. -8157.737226 +5.3 C_{2v}

WBI: Co1-Co2: 0.1461

		1	2	3	4	5
1	Co	0.000000				
2	Co	3.482690	0.000000			
3	Se	2.393188	2.393287	0.000000		
4	Se	2.390704	2.394160	3.199450	0.000000	
5	B	3.408271	3.409766	3.240713	4.019437	0.000000
6	B	2.369174	3.461723	2.125768	3.829747	1.694031
7	B	3.462137	2.376050	2.126317	3.835732	1.693688
8	B	3.839995	2.174642	3.312682	3.314521	1.806069
9	B	3.411735	3.409081	4.022821	3.240232	1.771818
10	B	2.175044	3.838353	3.315131	3.308336	1.805430
11	B	3.462669	2.369825	3.832784	2.126122	2.915896
12	B	2.379520	3.462133	3.838795	2.124463	2.917059
		6	7	8	9	10
6	B	0.000000				
7	B	1.824939	0.000000			
8	B	2.900065	1.779961	0.000000		
9	B	2.917055	2.917648	1.806216	0.000000	
10	B	1.780239	2.901201	2.874026	1.805306	0.000000
11	B	3.665814	3.182034	1.779451	1.694280	2.898080
12	B	3.183541	3.670970	2.900754	1.693337	1.779811
		11	12			
11	B	0.000000				
12	B	1.823071	0.000000			

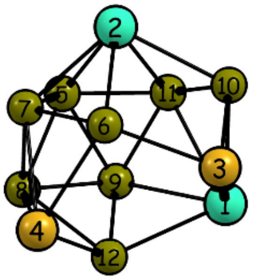

5. -8157.732622 +8.2 C_1

WBI: Co1-Co2: 0.0920

		1	2	3	4	5
1	Co	0.000000				
2	Co	3.606118	0.000000			
3	Se	2.365722	3.181615	0.000000		
4	Se	3.628613	3.707728	3.237742	0.000000	
5	B	3.317573	2.063767	4.016469	3.254929	0.000000
6	B	3.388278	2.157945	2.040278	2.155923	3.024589
7	B	3.861016	2.018791	3.575735	2.155420	1.749687
8	B	3.390450	3.323592	4.104965	2.079266	1.761910
9	B	2.112212	3.253008	3.713823	3.179488	1.784227
10	B	2.157637	2.119709	1.980791	4.096856	3.091131
11	B	2.161596	2.086506	3.220901	3.935132	1.762506
12	B	2.131401	3.918321	3.170202	2.062783	2.910556
		6	7	8	9	10
6	B	0.000000				
7	B	1.855156	0.000000			
8	B	3.084089	1.867421	0.000000		
9	B	3.519161	2.871998	1.768924	0.000000	
10	B	2.653715	3.413403	3.978822	3.091729	0.000000
11	B	3.179459	2.918251	2.940231	1.776695	1.745755
12	B	3.123366	3.005967	1.861254	1.716549	3.550426
		11	12			
11	B	0.000000				
12	B	2.999798	0.000000			

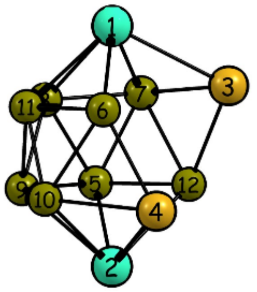

6. -8157.732613 +8.2 C_2

WBI: Co1-Co2: 0.0510

		1	2	3	4	5
1	Co	0.000000				
2	Co	4.218825	0.000000			
3	Se	2.333393	3.777263	0.000000		
4	Se	3.779553	2.333585	3.378083	0.000000	
5	B	3.258099	2.136265	3.156289	3.695099	0.000000
6	B	2.214212	3.305679	3.063733	2.003061	3.604895
7	B	2.188897	3.474513	2.030140	4.132164	1.777365
8	B	2.101073	3.295459	3.300123	4.036205	1.790290
9	B	3.296002	2.103416	4.037574	3.299887	1.766733
10	B	3.477852	2.191138	4.133130	2.029523	3.095943
11	B	2.138378	3.259561	3.695802	3.155387	2.905727
12	B	3.303876	2.209162	2.002294	3.060437	1.725796

		6	7	8	9	10
6	B	0.000000				
7	B	3.504674	0.000000			
8	B	2.998330	1.796057	0.000000		
9	B	2.923434	2.966632	1.739720	0.000000	
10	B	1.907091	3.924357	2.966408	1.795812	0.000000
11	B	1.724222	3.096480	1.766403	1.790275	1.778016
12	B	3.461122	1.907193	2.923546	2.998836	3.502667

		11	12
11	B	0.000000	
12	B	3.603843	0.000000

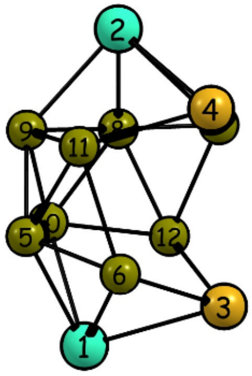

7. -8157.730869 +9.3 C_2

WBI: Co1-Co2: 0.0817

		1	2	3	4	5
1	Co	0.000000				
2	Co	4.821126	0.000000			
3	Se	2.273737	4.458150	0.000000		
4	Se	4.458216	2.274385	3.221869	0.000000	
5	B	2.112081	3.435085	3.168079	3.486204	0.000000
6	B	2.186253	3.890083	2.008960	2.826009	1.731441
7	B	3.891727	2.184355	2.828892	2.010320	3.516594
8	B	3.434509	2.112534	3.486341	3.169383	2.870463
9	B	3.361834	2.077451	3.993408	3.248294	1.770650
10	B	2.076949	3.361697	3.247561	3.993347	1.794700
11	B	3.528069	2.276516	3.454219	2.051038	1.792771
12	B	2.276958	3.527529	2.051628	3.454471	2.873774

		6	7	8	9	10
6	B	0.000000				
7	B	3.300128	0.000000			
8	B	3.515671	1.731288	0.000000		
9	B	3.031503	2.904251	1.795022	0.000000	
10	B	2.904072	3.031788	1.770047	1.728379	0.000000
11	B	2.006277	2.887525	2.873680	1.843051	2.901395
12	B	2.887450	2.007170	1.792118	2.901755	1.843079

		11	12
11	B	0.000000	
12	B	3.369950	0.000000

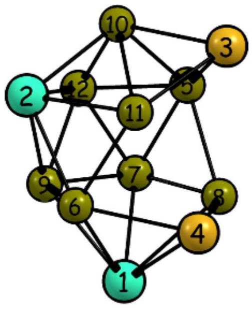

8. -8157.726844 +11.8 C_1

WBI: Co1-Co2: 0.1082

		1	2	3	4	5
1	Co	0.000000				
2	Co	3.726090	0.000000			
3	Se	4.481987	3.427710	0.000000		
4	Se	2.292292	3.684081	3.162132	0.000000	
5	B	3.474509	3.236592	2.088109	3.348660	0.000000
6	B	2.165813	2.022314	3.663006	2.102592	3.440620
7	B	2.094197	3.236887	3.556734	3.158013	1.794798
8	B	2.134387	3.904411	2.926500	2.032597	1.963860
9	B	2.098153	2.064757	4.080044	3.273957	2.922767
10	B	4.330457	2.053484	2.019267	3.912153	1.897903
11	B	3.587061	2.041990	2.068654	2.309956	2.906390
12	B	3.361894	2.085366	3.233741	3.915807	1.824921

		6	7	8	9	10
6	B	0.000000				
7	B	2.854068	0.000000			
8	B	2.957205	1.714512	0.000000		
9	B	1.797324	1.785190	2.923748	0.000000	
10	B	3.261341	2.994842	3.422663	3.011969	0.000000
11	B	1.954084	3.429537	3.095942	3.025532	2.228461
12	B	2.918601	1.767656	3.000701	1.758071	1.721291

		11	12
11	B	0.000000	
12	B	3.001908	0.000000

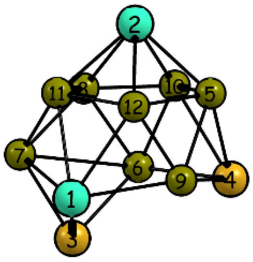

9. -8157.726153 +12.3 C_1

WBI: Co1-Co2: 0.0970

		1	2	3	4	5
1	Co	0.000000				
2	Co	3.717473	0.000000			
3	Se	2.373375	4.453202	0.000000		
4	Se	3.625907	3.604586	3.260713	0.000000	
5	B	3.396972	2.010858	4.162620	2.121221	0.000000
6	B	3.345633	3.381854	2.027225	2.104469	3.100840
7	B	2.143022	3.405137	1.975473	4.077803	3.981273
8	B	3.205182	2.070072	2.899729	3.354120	3.001320
9	B	2.131476	3.281054	3.209988	2.075872	1.846022
10	B	3.828547	1.984649	3.541874	2.159245	1.945873
11	B	2.158083	2.030687	3.210405	3.916551	2.938939
12	B	2.146451	2.080429	3.768247	3.203093	1.751815

		6	7	8	9	10
6	B	0.000000				
7	B	2.642314	0.000000			
8	B	1.948903	1.897476	0.000000		
9	B	3.078091	3.530495	3.467285	0.000000	
10	B	1.807363	3.342518	1.754715	2.999016	0.000000
11	B	3.148325	1.712682	1.778438	2.976803	2.869735
12	B	3.513776	3.095465	2.871398	1.719381	2.881404

		11	12
11	B	0.000000	
12	B	1.787159	0.000000

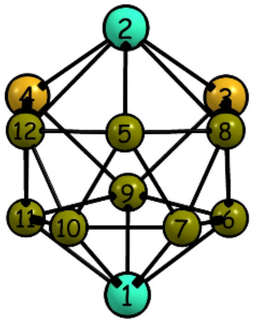

10. -8157.725873 +12.4 C_s

WBI: Co1-Co2: 0.1017

		1	2	3	4	5
1	Co	0.000000				
2	Co	4.261231	0.000000			
3	Se	3.583914	2.399625	0.000000		
4	Se	3.619228	2.396239	3.133712	0.000000	
5	B	3.269694	2.177656	3.316252	3.324962	0.000000
6	B	2.010875	3.466280	2.100766	3.831867	2.910123
7	B	2.106032	3.409952	3.245631	4.030941	1.793208
8	B	3.268875	2.338734	2.125718	3.802108	1.773451
9	B	2.023411	3.180342	2.194089	2.229738	3.382687
10	B	2.107438	3.404563	4.003483	3.252485	1.793925
11	B	2.010974	3.447280	3.772579	2.099402	2.907525
12	B	3.277855	2.331512	3.780875	2.118446	1.776731

		6	7	8	9	10
6	B	0.000000				
7	B	1.724991	0.000000			
8	B	1.831499	1.712841	0.000000		
9	B	2.075850	2.988231	3.059093	0.000000	
10	B	2.950066	1.781286	2.921136	2.981169	0.000000
11	B	3.206399	2.946757	3.660737	2.044737	1.728497
12	B	3.678339	2.926279	3.150431	3.058148	1.714274

		11	12
11	B	0.000000	
12	B	1.836853	0.000000

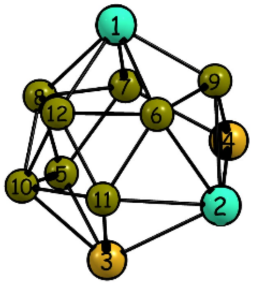

11. -8157.725041 +13.0 C_1

WBI: Co1-Co2: 0.1008

		1	2	3	4	5
1	Co	0.000000				
2	Co	3.685081	0.000000			
3	Se	4.237664	2.415419	0.000000		
4	Se	3.461835	2.373171	3.233323	0.000000	
5	B	3.344356	3.476318	2.019189	3.026071	0.000000
6	B	2.050195	2.191326	3.321088	3.336235	3.538880
7	B	2.104167	3.319264	3.261770	2.081498	1.922217
8	B	2.055200	3.869982	3.133216	3.592505	1.692025
9	B	2.024142	2.235582	3.738462	2.003854	3.536054
10	B	3.301914	3.541964	2.094438	4.153260	1.819055
11	B	3.244879	2.267421	2.116992	3.852903	2.976979
12	B	2.052520	3.391278	3.207422	4.160817	2.894805

		6	7	8	9	10
6	B	0.000000				
7	B	2.993473	0.000000			
8	B	2.893887	1.768749	0.000000		
9	B	1.804879	2.205943	3.170706	0.000000	
10	B	2.982798	3.028642	1.777380	3.886926	0.000000
11	B	1.776035	3.480089	2.866360	3.149598	1.871681
12	B	1.769992	2.955645	1.812314	3.051347	1.765706

		11	12
11	B	0.000000	
12	B	1.724413	0.000000

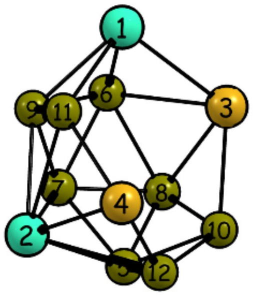

12. -8157.724574 +13.2 C_1

WBI: Co1-Co2: 0.0923

		1	2	3	4	5
1	Co	0.000000				
2	Co	3.743886	0.000000			
3	Se	2.317747	4.063585	0.000000		
4	Se	3.537117	2.386853	3.757324	0.000000	
5	B	4.061978	2.185295	3.121214	3.322310	0.000000
6	B	2.067758	3.382982	2.160503	4.234797	2.940558
7	B	3.264240	2.177201	3.152181	3.862616	1.771817
8	B	3.483601	3.369787	2.143761	4.242588	1.722121
9	B	2.037968	2.226541	3.191546	3.335211	3.037870
10	B	3.745384	3.321816	2.024511	3.258572	1.711392
11	B	2.062897	2.271378	3.384381	1.972894	3.552800
12	B	4.090759	2.268864	3.273974	1.951980	1.788455

		6	7	8	9	10
6	B	0.000000				
7	B	1.749435	0.000000			
8	B	1.892869	1.765050	0.000000		
9	B	1.755780	1.768094	2.983480	0.000000	
10	B	3.076843	2.871262	1.781289	3.643912	0.000000
11	B	3.052783	3.102102	3.913790	1.778406	3.739776
12	B	3.862312	3.114510	3.021302	3.558482	1.796190

		11	12
11	B	0.000000	
12	B	3.115194	0.000000

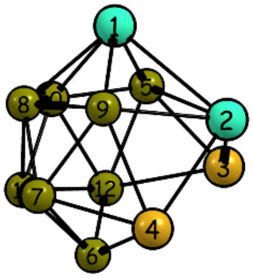

13. -8157.717297 +17.8 C_1

WBI: Co1-Co2: 0.4174

		1	2	3	4	5
1	Co	0.000000				
2	Co	2.651635	0.000000			
3	Se	3.288009	2.335757	0.000000		
4	Se	3.836583	2.330014	3.248481	0.000000	
5	B	2.119438	3.079509	1.970723	4.120989	0.000000
6	B	3.923682	3.420817	2.915116	2.041947	3.428640
7	B	3.366738	3.468593	4.007466	2.069772	3.922640
8	B	2.077875	3.498619	3.949577	3.253609	3.044766
9	B	2.084152	2.194605	3.610046	2.210358	3.406869
10	B	2.085763	3.731248	3.161127	3.986122	1.711238
11	B	3.259752	3.983756	3.523254	3.158960	3.000338
12	B	3.234918	3.518348	2.090905	3.394306	1.880023

		6	7	8	9	10
6	B	0.000000				
7	B	1.892275	0.000000			
8	B	2.890240	1.736666	0.000000		
9	B	3.020062	1.893230	1.748392	0.000000	
10	B	2.975206	2.936133	1.765787	2.950975	0.000000
11	B	1.694778	1.759456	1.784897	2.888962	1.765391
12	B	1.907904	3.040107	2.895158	3.478959	1.824787

		11	12
11	B	0.000000	
12	B	1.780545	0.000000

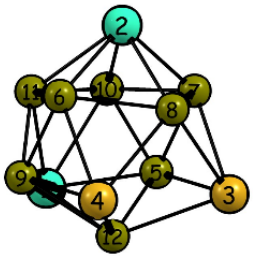

14. -8157.712756 +20.6 C_{2v}

WBI: Co1-Co2: 0.1162

		1	2	3	4	5
1	Co	0.000000				
2	Co	3.655464	0.000000			
3	Se	3.603229	3.597069	0.000000		
4	Se	3.602945	3.614844	3.100785	0.000000	
5	B	2.009409	3.269451	2.090977	3.778134	0.000000
6	B	3.270356	2.010610	3.758937	2.088273	3.665980
7	B	3.273553	2.007829	2.089866	3.787618	1.824169
8	B	3.801775	2.011444	2.215618	2.238288	3.053544
9	B	2.008755	3.275944	3.771655	2.087900	3.187631
10	B	2.094807	2.094206	3.259306	4.026653	1.745951
11	B	2.095325	2.095507	4.015855	3.263605	2.955253
12	B	2.013133	3.802482	2.225293	2.220044	2.039330
		6	7	8	9	10
6	B	0.000000				
7	B	3.184271	0.000000			
8	B	2.028280	2.037201	0.000000		
9	B	1.825975	3.677338	3.059195	0.000000	
10	B	2.954874	1.746898	2.989271	2.957762	0.000000
11	B	1.749208	2.957199	2.989259	1.748309	1.784549
12	B	3.047416	3.061294	2.846940	2.033409	2.991997
		11	12			
11	B	0.000000				
12	B	2.989357	0.000000			

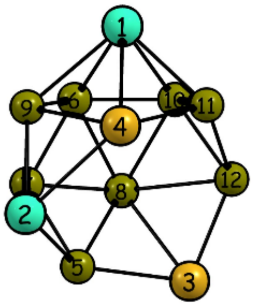

15. -8157.706295 +24.7 C_1

WBI: Co1-Co2: 0.0974

		1	2	3	4	5
1	Co	0.000000				
2	Co	3.818905	0.000000			
3	Se	4.656133	3.573974	0.000000		
4	Se	2.383875	2.321665	3.805815	0.000000	
5	B	4.294090	2.037358	1.978267	3.352314	0.000000
6	B	2.066628	3.265280	3.801457	3.167427	3.042514
7	B	3.350846	2.056001	3.304360	3.212243	1.738592
8	B	3.389815	3.331065	2.233372	3.622032	1.993907
9	B	2.180201	2.055404	4.247539	2.211128	2.997461
10	B	2.041522	4.030634	3.226035	3.123360	3.460721
11	B	2.037511	3.734062	3.273598	2.005306	3.702847
12	B	3.387889	4.109408	1.926354	3.300004	3.103085
		6	7	8	9	10
6	B	0.000000				
7	B	1.754746	0.000000			
8	B	1.836526	1.866105	0.000000		
9	B	1.772346	1.741797	2.953055	0.000000	
10	B	1.748732	2.993464	1.910790	2.965403	0.000000
11	B	2.884664	3.651224	3.009130	3.123802	1.746167
12	B	3.110355	3.566091	2.140416	3.912860	1.758961
		11	12			
11	B	0.000000				
12	B	1.829644	0.000000			

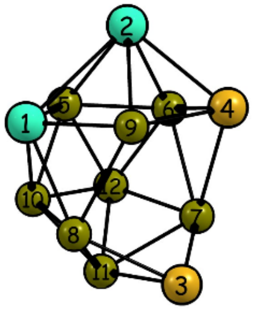

16. -8157.702565 +27.0 C_1

WBI: Co1-Co2: 0.3345

		1	2	3	4	5
1	Co	0.000000				
2	Co	2.548526	0.000000			
3	Se	3.894869	4.604386	0.000000		
4	Se	3.521270	2.278213	3.160888	0.000000	
5	B	2.094365	2.100770	4.079746	3.224342	0.000000
6	B	3.326771	2.146855	3.606043	2.063461	1.832321
7	B	3.902258	3.705846	2.062574	2.490691	3.006964
8	B	2.097766	3.790473	2.069411	3.456242	2.948631
9	B	1.934628	2.271754	3.016266	2.009828	2.889898
10	B	2.064982	3.510664	3.235300	3.903422	1.774866
11	B	3.379046	4.387840	2.036894	3.824856	2.970898
12	B	3.271093	3.388779	3.201732	3.345519	1.793091

		6	7	8	9	10
6	B	0.000000				
7	B	1.943381	0.000000			
8	B	3.471231	2.872135	0.000000		
9	B	2.958374	3.248510	2.217004	0.000000	
10	B	2.903992	2.883900	1.785182	3.076946	0.000000
11	B	3.089171	1.925849	1.882159	3.517769	1.730298
12	B	1.750975	1.748677	2.851433	3.536654	1.777616

		11	12
11	B	0.000000	
12	B	1.765680	0.000000

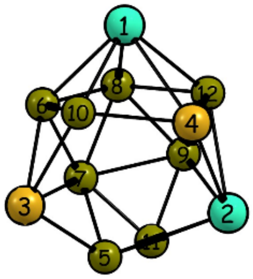

17. -8157.701878 +27.4 C_1

WBI: Co1-Co2: 0.0857

		1	2	3	4	5
1	Co	0.000000				
2	Co	3.841090	0.000000			
3	Se	3.671042	3.735229	0.000000		
4	Se	2.360130	2.384785	3.356995	0.000000	
5	B	4.173321	2.318713	2.005541	3.321299	0.000000
6	B	2.128082	3.808343	2.128230	3.188086	2.920788
7	B	3.459443	3.045477	2.692064	3.794040	2.004611
8	B	2.050579	3.220760	3.470231	3.192915	3.250675
9	B	3.393694	2.154055	4.018137	3.409723	2.821127
10	B	2.066316	3.607226	2.012836	2.017577	3.143594
11	B	4.371754	2.066121	3.407263	3.835272	1.645445
12	B	2.147167	2.199306	4.063609	2.159540	3.451620

		6	7	8	9	10
6	B	0.000000				
7	B	1.989057	0.000000			
8	B	1.747595	1.788623	0.000000		
9	B	3.102970	1.891060	1.776014	0.000000	
10	B	1.893236	3.330916	2.981130	3.938751	0.000000
11	B	3.394719	1.687707	2.855552	1.671435	4.106224
12	B	2.954083	2.892801	1.775488	1.734803	3.129707

		11	12
11	B	0.000000	
12	B	3.006969	0.000000

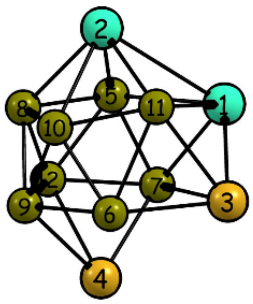

18. -8157.696229 +31.0 C_1

WBI: Co1-Co2: 0.3611

		1	2	3	4	5
1	Co	0.000000				
2	Co	2.552954	0.000000			
3	Se	2.360985	3.736216	0.000000		
4	Se	3.638164	4.391171	3.275491	0.000000	
5	B	2.154054	2.087653	3.777092	3.160307	0.000000
6	B	3.297068	3.345767	2.007367	2.595766	3.540126
7	B	2.161946	3.446408	3.165617	2.018209	1.726214
8	B	3.405159	2.021663	4.143818	3.206073	1.832155
9	B	3.838127	3.273670	3.586273	2.062182	2.877900
10	B	3.358716	2.084384	3.173878	3.408423	2.913369
11	B	2.355043	2.069921	2.033577	3.919371	3.138501
12	B	3.444798	3.320842	4.182748	2.042515	1.793638

		6	7	8	9	10
6	B	0.000000				
7	B	3.294793	0.000000			
8	B	2.986805	2.915635	0.000000		
9	B	1.945167	2.933699	1.759331	0.000000	
10	B	1.741332	3.546247	1.778520	1.778490	0.000000
11	B	1.962325	3.537391	3.013311	3.117496	1.778242
12	B	3.315383	1.856157	1.757561	1.886603	2.998440

		11	12
11	B	0.000000	
12	B	3.891348	0.000000

Table S6A: Distance table for the lowest-lying $Cp^{*}{}_2Co_2S_2B_7H_7$ optimized structures obtained at the PBE0/def2-TZVP level of theory. Included are the zero-point corrected absolute energy in (a.u.) at the DLPNO-CCSD(T)/def2-QZVP level of theory with zero-point energy obtained from the PBE0/def2-TZVP computations, relative energy in (kcal/mol), symmetry and Wiberg bond index. For clarity, only the atoms forming the cluster framework are shown.

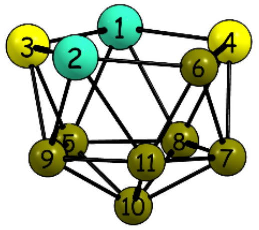

1. -4518.491151 0.0 C_1

WBI: Co1-Co2: 0.0892

		1	2	3	4	5
1	Co	0.000000				
2	Co	3.757147	0.000000			
3	S	2.206474	2.188473	0.000000		
4	S	2.211311	3.402753	3.241532	0.000000	
5	B	2.109054	3.346776	1.959723	3.118927	0.000000
6	B	3.432187	2.045455	3.141821	1.913051	3.484415
7	B	3.401131	3.299780	3.674585	1.988792	2.936553
8	B	2.098777	3.889231	3.109839	1.995574	1.785936
9	B	3.406997	2.070048	1.989302	3.622788	1.873202
10	B	3.288290	3.268865	3.061435	3.055670	1.758541
11	B	3.924900	2.052401	3.110868	3.032114	2.939589
		6	7	8	9	10
6	B	0.000000				
7	B	1.834106	0.000000			
8	B	2.979400	1.850354	0.000000		
9	B	2.901817	2.888507	2.936901	0.000000	
10	B	2.885172	1.755769	1.753900	1.770514	0.000000
11	B	1.736986	1.751872	2.916630	1.775267	1.802122
		11				
11	B	0.000000				

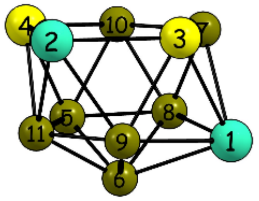

2. -4518.490244 +0.6 C_1

WBI: Co1-Co2: 0.0856

		1	2	3	4	5
1	Co	0.000000				
2	Co	3.765919	0.000000			
3	S	2.226706	2.212188	0.000000		
4	S	4.080403	2.189668	3.188637	0.000000	
5	B	3.310514	3.400472	3.650824	1.989520	0.000000
6	B	2.095802	3.307591	3.104904	3.065820	1.767147
7	B	2.106504	3.386076	1.890866	3.028838	2.918949
8	B	2.073212	3.900421	3.055732	3.027761	1.750462
9	B	2.121849	2.119235	2.019164	3.111700	2.943467
10	B	3.303221	3.386863	3.050501	1.885120	1.802543
11	B	3.325643	2.097190	3.115960	1.997304	1.866320
		6	7	8	9	10
6	B	0.000000				
7	B	2.915387	0.000000			
8	B	1.795291	1.778076	0.000000		
9	B	1.772250	2.969900	2.909668	0.000000	
10	B	2.876910	1.780026	1.739815	3.451299	0.000000
11	B	1.753966	3.452445	2.900112	1.776039	2.947863
		11				
11	B	0.000000				

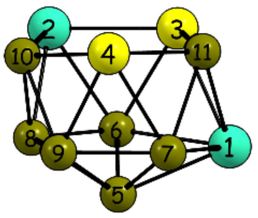

3. -4518.480325 +6.7 C_1

WBI: Co1-Co2: 0.0822

		1	2	3	4	5
1	Co	0.000000				
2	Co	3.744219	0.000000			
3	S	2.248191	2.189610	0.000000		
4	S	3.473538	3.388385	3.133849	0.000000	
5	B	2.079996	3.285110	3.115870	3.075046	0.000000
6	B	2.179834	2.060981	2.047631	3.596511	1.783236
7	B	2.040049	3.914261	3.115389	2.031027	1.752877
8	B	3.346369	2.055957	3.122214	3.038685	1.794420
9	B	3.325356	3.295251	3.641377	1.981082	1.770370
10	B	3.946850	2.008767	3.083426	1.950805	2.909320
11	B	2.074542	3.358563	1.869599	1.926846	2.922444

		6	7	8	9	10
6	B	0.000000				
7	B	2.934145	0.000000			
8	B	1.762595	2.916511	0.000000		
9	B	2.885935	1.840262	1.763165	0.000000	
10	B	2.877343	3.031740	1.737464	1.852446	0.000000
11	B	2.985630	1.874667	3.463164	2.988706	3.038615

		11
11	B	0.000000

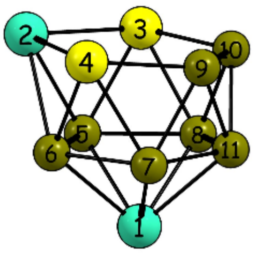

4. -4518.479282 +7.4 C_s

WBI: Co1-Co2: 0.0693

		1	2	3	4	5
1	Co	0.000000				
2	Co	3.672247	0.000000			
3	S	3.441763	2.198669	0.000000		
4	S	3.443300	2.197893	3.202763	0.000000	
5	B	2.027720	2.121591	1.991142	3.121142	0.000000
6	B	2.028644	2.121109	3.120648	1.991268	1.803158
7	B	2.048886	3.427736	3.647257	1.990075	2.980468
8	B	2.049001	3.429452	1.989715	3.648703	1.905060
9	B	3.282153	3.379402	3.032179	1.907221	3.470370
10	B	3.282092	3.380171	1.907630	3.032540	2.986447
11	B	2.075241	3.912246	3.043531	3.043969	2.937875

		6	7	8	9	10
6	B	0.000000				
7	B	1.902943	0.000000			
8	B	2.982147	2.918755	0.000000		
9	B	2.985370	1.826049	2.897956	0.000000	
10	B	3.470583	2.897994	1.825850	1.735044	0.000000
11	B	2.937597	1.756139	1.756168	1.757256	1.757379

		11
11	B	0.000000

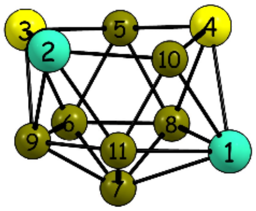

5. -4518.476971 +8.8 C_1

WBI: Co1-Co2: 0.0772

		1	2	3	4	5
1	Co	0.000000				
2	Co	3.712635	0.000000			
3	S	4.079958	2.174852	0.000000		
4	S	2.233546	3.375686	3.119532	0.000000	
5	B	3.313217	3.357104	1.885674	1.908108	0.000000
6	B	3.289074	3.355172	1.993232	3.100701	1.802385
7	B	2.100889	3.277983	3.085834	3.138281	2.904663
8	B	2.081666	3.905154	3.082215	2.090474	1.825230
9	B	3.318062	2.059287	2.017372	3.612504	2.972391
10	B	2.130716	2.047830	3.093676	1.922384	3.023365
11	B	2.105851	2.065220	3.111455	3.066274	3.452816

		6	7	8	9	10
6	B	0.000000				
7	B	1.761322	0.000000			
8	B	1.728033	1.767481	0.000000		
9	B	1.880691	1.764712	2.917230	0.000000	
10	B	3.489850	2.929276	3.029492	2.906318	0.000000
11	B	2.934868	1.796535	2.921458	1.775416	1.762865

		11
11	B	0.000000

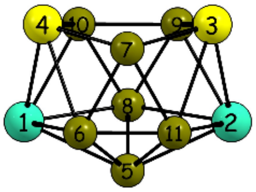

6. -4518.476139 +9.5 C_s

WBI: Co1-Co2: 0.0756

		1	2	3	4	5
1	Co	0.000000				
2	Co	3.754240	0.000000			
3	S	4.055645	2.226495	0.000000		
4	S	2.225944	4.055952	3.061462	0.000000	
5	B	2.120084	2.121255	3.171997	3.172673	0.000000
6	B	2.107647	3.291213	3.121666	2.133184	1.761404
7	B	3.286118	3.288674	1.899376	1.899191	2.909865
8	B	2.098675	2.098869	3.057371	3.055787	1.784023
9	B	3.321773	2.071514	1.888762	3.008842	2.937958
10	B	2.075289	3.320862	3.009908	1.888263	2.939397
11	B	3.288157	2.110907	2.133064	3.122428	1.759907

		6	7	8	9	10
6	B	0.000000				
7	B	1.813413	0.000000			
8	B	2.892516	3.398828	0.000000		
9	B	3.485414	2.986709	1.771480	0.000000	
10	B	3.018355	2.987102	1.771139	1.793215	0.000000
11	B	1.697543	1.814734	2.892545	3.017249	3.486297

		11
11	B	0.000000

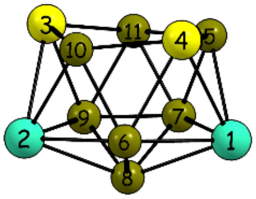

7. -4518.472901 +11.4 C_1

WBI: Co1-Co2: 0.0719

		1	2	3	4	5
1	Co	0.000000				
2	Co	3.736190	0.000000			
3	S	4.044211	2.255196	0.000000		
4	S	2.220181	3.497661	3.070268	0.000000	
5	B	2.128750	3.917250	2.961817	1.913176	0.000000
6	B	2.114161	2.050093	3.107832	2.098053	3.033535
7	B	2.093569	3.307740	3.047963	3.067683	1.795855
8	B	2.128481	2.107063	3.136974	3.156151	2.962253
9	B	3.312447	2.170735	2.089225	3.642466	2.900038
10	B	3.344511	2.067759	1.873824	1.912451	2.985152
11	B	3.296559	3.340689	1.898500	3.029592	1.742180
		6	7	8	9	10
6	B	0.000000				
7	B	2.899988	0.000000			
8	B	1.763756	1.788278	0.000000		
9	B	2.933938	1.717903	1.776679	0.000000	
10	B	1.874016	3.441367	2.952702	3.024205	0.000000
11	B	3.473251	1.743137	2.905053	1.799864	2.995920
		11				
11	B	0.000000				

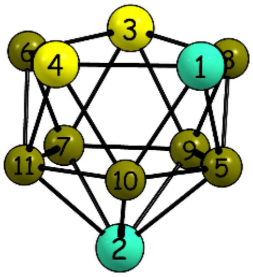

8. -4518.471073 +12.5 C_1

WBI: Co1-Co2: 0.0732

		1	2	3	4	5
1	Co	0.000000				
2	Co	3.668054	0.000000			
3	S	3.370562	3.457395	0.000000		
4	S	2.190896	3.462228	3.140597	0.000000	
5	B	2.066882	2.098024	3.059369	3.130918	0.000000
6	B	3.348091	3.260231	1.900299	1.890924	3.468623
7	B	3.909328	2.007266	2.025268	3.090881	2.954000
8	B	2.023216	3.282699	1.940340	3.108654	1.733645
9	B	3.297751	2.035210	2.029489	3.662717	1.759045
10	B	2.073101	2.059884	3.612927	2.024169	1.787307
11	B	3.377412	2.009436	3.091838	1.993601	2.969794
		6	7	8	9	10
6	B	0.000000				
7	B	1.817440	0.000000			
8	B	3.024293	3.017527	0.000000		
9	B	3.001201	1.895555	1.828910	0.000000	
10	B	2.992103	2.957498	2.892763	2.907336	0.000000
11	B	1.815929	1.757501	3.496836	2.951753	1.909975
		11				
11	B	0.000000				

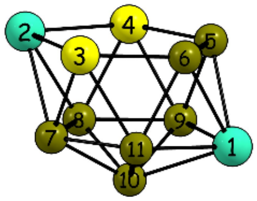

9. -4518.470174 +13.2 C_s

WBI: Co1-Co2: 0.0409

		1	2	3	4	5
1	Co	0.000000				
2	Co	4.386939	0.000000			
3	S	3.446352	2.200753	0.000000		
4	S	3.445810	2.201026	3.191208	0.000000	
5	B	2.076501	3.399813	3.024061	1.924472	0.000000
6	B	2.077985	3.399834	1.923427	3.025347	1.706957
7	B	3.306193	2.116136	1.969002	3.102830	3.466843
8	B	3.305459	2.116602	3.102517	1.969855	2.991294
9	B	2.021814	3.417246	3.633200	1.991596	1.886820
10	B	2.078811	3.311904	3.065906	3.066242	2.940273
11	B	2.023351	3.418874	1.993495	3.635370	2.916681

		6	7	8	9	10
6	B	0.000000				
7	B	2.990806	0.000000			
8	B	3.467394	1.801179	0.000000		
9	B	2.915863	2.941181	1.853859	0.000000	
10	B	2.940599	1.763174	1.762602	1.775179	0.000000
11	B	1.888579	1.854247	2.941504	2.894764	1.774602

		11
11	B	0.000000

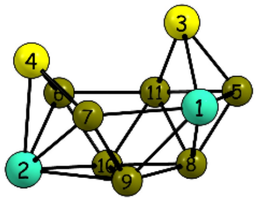

10. -4518.458889 +20.2 C_1

WBI: Co1-Co2: 0.0709

		1	2	3	4	5
1	Co	0.000000				
2	Co	3.862581	0.000000			
3	S	2.273499	4.294348	0.000000		
4	S	3.536551	2.193749	3.154805	0.000000	
5	B	2.095433	4.574947	1.796742	4.274594	0.000000
6	B	3.706133	2.120988	2.956895	1.854536	3.564172
7	B	2.225323	2.144255	3.138918	1.859931	3.691786
8	B	2.152192	3.398990	2.823006	3.914140	1.701862
9	B	2.150072	2.112547	3.426292	3.045272	3.091555
10	B	3.208626	2.066804	3.311986	3.013906	2.999070
11	B	2.946060	3.449905	1.967275	3.232013	1.841080

		6	7	8	9	10
6	B	0.000000				
7	B	2.759590	0.000000			
8	B	3.038049	3.056265	0.000000		
9	B	2.833097	1.742798	1.770062	0.000000	
10	B	1.726250	2.802719	1.757986	1.787487	0.000000
11	B	1.937775	3.336199	1.862049	2.827346	1.786917

		11
11	B	0.000000

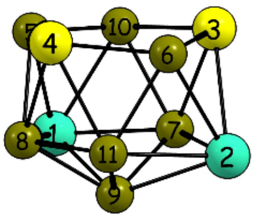

11. -4518.458727 +20.3 C_1

WBI: Co1-Co2: 0.0755

		1	2	3	4	5
1	Co	0.000000				
2	Co	3.702863	0.000000			
3	S	3.460683	2.240725	0.000000		
4	S	3.442935	3.463642	3.090622	0.000000	
5	B	2.072853	3.937202	2.977481	1.951692	0.000000
6	B	3.911118	2.071874	1.875812	1.901887	3.001272
7	B	2.009345	2.164885	2.058035	3.618187	2.906061
8	B	2.019540	3.340942	3.604738	2.001420	1.915509
9	B	2.065640	2.104615	3.123968	3.093024	2.975444
10	B	2.071529	3.414687	1.941183	3.014566	1.679037
11	B	3.288346	2.070308	3.105186	2.011828	3.041251
		6	7	8	9	10
6	B	0.000000				
7	B	3.012789	0.000000			
8	B	2.993514	2.887370	0.000000		
9	B	2.943384	1.793579	1.776665	0.000000	
10	B	3.006329	1.892591	2.915710	2.972936	0.000000
11	B	1.873296	2.939502	1.838310	1.756756	3.499287
		11				
11	B	0.000000				

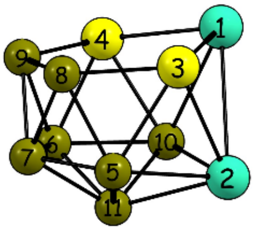

12. -4518.439289 +32.5 C_1

WBI: Co1-Co2: 0.3378

		1	2	3	4	5
1	Co	0.000000				
2	Co	2.658016	0.000000			
3	S	2.198967	2.253310	0.000000		
4	S	2.197166	3.630043	3.231144	0.000000	
5	B	3.542898	2.139365	2.029037	3.629445	0.000000
6	B	3.464421	3.387791	3.638656	1.932488	2.875135
7	B	3.959669	3.319489	3.058904	3.002856	1.719332
8	B	3.347496	3.372483	1.920875	3.023760	1.808785
9	B	3.343187	3.979007	3.053149	1.889869	2.877793
10	B	2.258507	2.128349	3.075175	2.005385	2.938241
11	B	3.478933	2.076939	3.087270	3.039947	1.787295
		6	7	8	9	10
6	B	0.000000				
7	B	1.759102	0.000000			
8	B	2.899959	1.756389	0.000000		
9	B	1.859021	1.752517	1.733934	0.000000	
10	B	1.881140	2.920132	3.438510	3.001420	0.000000
11	B	1.746913	1.792791	2.895526	2.900274	1.762583
		11				
11	B	0.000000				

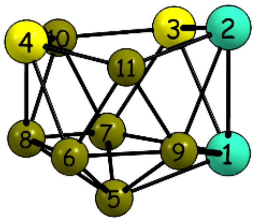

13. -4518.435922 +34.7 C_1

WBI: Co1-Co2: 0.3446

		1	2	3	4	5
1	Co	0.000000				
2	Co	2.611199	0.000000			
3	S	2.258345	2.199189	0.000000		
4	S	4.094758	3.278476	3.168677	0.000000	
5	B	2.094917	3.461892	3.120765	3.074628	0.000000
6	B	3.312290	3.344598	3.663573	2.034887	1.730550
7	B	2.120850	3.490597	2.035281	3.083982	1.760285
8	B	3.306817	3.933484	3.101102	2.021387	1.745176
9	B	2.117421	2.180337	3.088077	3.049932	1.828332
10	B	3.344687	3.292046	1.903194	1.906274	2.886379
11	B	3.396269	2.034757	3.173887	1.916131	2.883892

		6	7	8	9	10
6	B	0.000000				
7	B	2.900089	0.000000			
8	B	1.862788	1.719972	0.000000		
9	B	1.771876	2.932206	2.931863	0.000000	
10	B	2.979448	1.807958	1.809151	3.424345	0.000000
11	B	1.787183	3.502602	2.979546	1.729491	3.030266

		11
11	B	0.000000

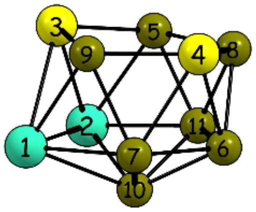

14. -4518.420847 +44.1 C_1

WBI: Co1-Co2: 0.2438

		1	2	3	4	5
1	Co	0.000000				
2	Co	2.797217	0.000000			
3	S	2.352888	2.369558	0.000000		
4	S	3.461666	4.157616	3.133528	0.000000	
5	B	3.532359	2.077548	1.912077	2.995024	0.000000
6	B	3.378556	3.351740	3.620477	1.987298	2.910009
7	B	2.008314	3.485741	3.135162	2.045932	3.476953
8	B	4.005161	3.288530	3.043747	1.892203	1.755363
9	B	2.032253	3.527941	1.882615	1.911229	2.945882
10	B	2.176694	2.173415	3.096277	3.117071	2.931510
11	B	3.482594	2.031246	3.070439	3.016162	1.790630

		6	7	8	9	10
6	B	0.000000				
7	B	1.818981	0.000000			
8	B	1.820473	2.981997	0.000000		
9	B	2.976520	1.906984	2.972013	0.000000	
10	B	1.811791	1.757854	2.926792	2.934512	0.000000
11	B	1.743330	2.884647	1.733524	3.425621	1.810883

		11
11	B	0.000000

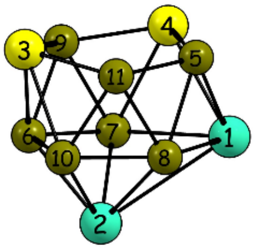

15. -4518.409861 +51.0 C_1

WBI: Co1-Co2: 0.3057

		1	2	3	4	5
1	Co	0.000000				
2	Co	2.708923	0.000000			
3	S	4.117506	3.466090	0.000000		
4	S	2.223483	3.680868	3.067141	0.000000	
5	B	2.085928	3.489533	2.962077	1.905329	0.000000
6	B	3.476073	1.987024	2.018119	3.098530	3.469711
7	B	2.201012	2.032911	3.128039	2.141875	3.022384
8	B	2.120137	2.132862	3.068682	3.075250	1.828268
9	B	3.362537	3.292304	1.911394	1.872942	2.957580
10	B	3.468864	2.027478	2.030415	3.656103	2.911228
11	B	3.304358	3.306267	1.916869	3.016397	1.725033

		6	7	8	9	10
6	B	0.000000				
7	B	1.756728	0.000000			
8	B	2.911867	2.829326	0.000000		
9	B	1.768225	1.864193	3.410131	0.000000	
10	B	1.935065	2.944717	1.767347	3.003533	0.000000
11	B	2.974398	3.455089	1.766503	3.001493	1.768103

		11
11	B	0.000000

S77

Table S6B: Distance table for the lowest-lying $Cp^*{}_2Co_2Se_2B_7H_7$ optimized structures obtained at the PBE0/def2-TZVP level of theory. Included are the zero-point corrected absolute energy in (a.u.) at the DLPNO-CCSD(T)/def2-QZVP level of theory with zero-point energy obtained from the PBE0/def2-TZVP computations, relative energy in (kcal/mol), symmetry and Wiberg bond index. For clarity, only the atoms forming the cluster framework are shown.

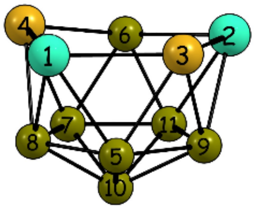

1. -8524.899054 0.0 C_1

WBI: Co1-Co2: 0.0909

		1	2	3	4	5
1	Co	0.000000				
2	Co	3.937324	0.000000			
3	Se	2.325568	2.312542	0.000000		
4	Se	2.331751	3.545503	3.422993	0.000000	
5	B	2.134055	3.426034	2.101879	3.260197	0.000000
6	B	3.606553	2.046838	3.280180	2.059994	3.548332
7	B	3.474534	3.313049	3.811497	2.119593	2.947791
8	B	2.118479	3.955991	3.243732	2.137340	1.777216
9	B	3.493537	2.095076	2.127388	3.775425	1.904726
10	B	3.326317	3.290206	3.201822	3.201129	1.754033
11	B	4.025028	2.053741	3.249645	3.181032	2.958860

		6	7	8	9	10
6	B	0.000000				
7	B	1.849306	0.000000			
8	B	3.056920	1.877791	0.000000		
9	B	2.915143	2.888456	2.954691	0.000000	
10	B	2.902542	1.755304	1.751711	1.769704	0.000000
11	B	1.731787	1.750318	2.939453	1.769059	1.798874

		11
11	B	0.000000

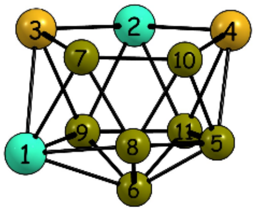

2. -8524.896959 +1.3 C_1

WBI: Co1-Co2: 0.0960

		1	2	3	4	5
1	Co	0.000000				
2	Co	3.827484	0.000000			
3	Se	2.340210	2.329712	0.000000		
4	Se	4.228151	2.310875	3.365483	0.000000	
5	B	3.311550	3.472019	3.786910	2.122032	0.000000
6	B	2.099975	3.337327	3.248941	3.211454	1.766472
7	B	2.125653	3.565015	2.033992	3.179194	2.929637
8	B	2.071717	3.992154	3.191982	3.176560	1.747028
9	B	2.147827	2.138302	2.177255	3.256711	2.956221
10	B	3.313855	3.554960	3.182882	2.029348	1.814633
11	B	3.340426	2.118549	3.259897	2.142536	1.894834

		6	7	8	9	10
6	B	0.000000				
7	B	2.940570	0.000000			
8	B	1.792502	1.770689	0.000000		
9	B	1.765319	3.065379	2.931504	0.000000	
10	B	2.894096	1.780458	1.735791	3.519439	0.000000
11	B	1.750326	3.529795	2.923549	1.768490	3.025739

		11
11	B	0.000000

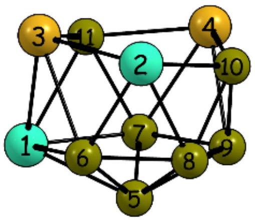

3. -8524.885858 +8.3 C_1

WBI: Co1-Co2: 0.0823

		1	2	3	4	5
1	Co	0.000000				
2	Co	3.811285	0.000000			
3	Se	2.363491	2.312728	0.000000		
4	Se	3.632110	3.542559	3.338327	0.000000	
5	B	2.080491	3.296473	3.248652	3.222688	0.000000
6	B	2.219496	2.077885	2.193217	3.772332	1.782535
7	B	2.044715	3.968024	3.244825	2.166183	1.752027
8	B	3.367597	2.055179	3.263744	3.188244	1.791526
9	B	3.347378	3.310535	3.785545	2.111359	1.768698
10	B	4.007616	2.014231	3.223652	2.089054	2.921725
11	B	2.083344	3.536162	2.015776	2.068039	2.955448

		6	7	8	9	10
6	B	0.000000				
7	B	2.961520	0.000000			
8	B	1.756413	2.934696	0.000000		
9	B	2.892090	1.865844	1.759364	0.000000	
10	B	2.893737	3.091975	1.732726	1.870059	0.000000
11	B	3.093672	1.870601	3.566382	3.060720	3.200214

		11
11	B	0.000000

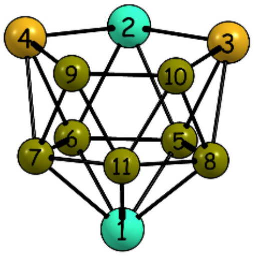

4. -8524.883734 +9.6 C_s

WBI: Co1-Co2: 0.0786

		1	2	3	4	5
1	Co	0.000000				
2	Co	3.714283	0.000000			
3	Se	3.595300	2.318416	0.000000		
4	Se	3.595438	2.318077	3.390776	0.000000	
5	B	2.028724	2.145987	2.137997	3.263266	0.000000
6	B	2.028774	2.145969	3.263736	2.139388	1.792049
7	B	2.049978	3.505727	3.793205	2.122320	2.994027
8	B	2.050957	3.505945	2.121533	3.794204	1.933550
9	B	3.301240	3.551011	3.173973	2.050196	3.536013
10	B	3.301760	3.552090	2.050228	3.175471	3.066446
11	B	2.069740	4.010677	3.188773	3.189524	2.959660

		6	7	8	9	10
6	B	0.000000				
7	B	1.934915	0.000000			
8	B	2.995292	2.916625	0.000000		
9	B	3.067402	1.842782	2.906943	0.000000	
10	B	3.538239	2.906927	1.843074	1.732787	0.000000
11	B	2.961082	1.752003	1.752504	1.751315	1.751196

		11
11	B	0.000000

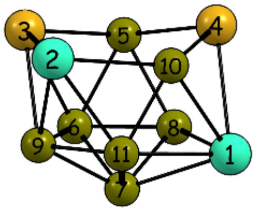

5. -8524.882084 +10.6 C_1

WBI: Co1-Co2: 0.0891

		1	2	3	4	5
1	Co	0.000000				
2	Co	3.734290	0.000000			
3	Se	4.242233	2.302607	0.000000		
4	Se	2.346902	3.521244	3.323345	0.000000	
5	B	3.380845	3.525616	2.030435	2.048200	0.000000
6	B	3.304368	3.423813	2.129533	3.241321	1.807304
7	B	2.102982	3.296600	3.229884	3.277061	2.929529
8	B	2.105592	3.968697	3.231878	2.225124	1.829335
9	B	3.326377	2.079325	2.152517	3.766351	3.052058
10	B	2.150308	2.057611	3.257123	2.067712	3.194688
11	B	2.106192	2.065766	3.258179	3.205594	3.543325

		6	7	8	9	10
6	B	0.000000				
7	B	1.755216	0.000000			
8	B	1.726601	1.763287	0.000000		
9	B	1.909970	1.765950	2.939375	0.000000	
10	B	3.554194	2.947841	3.103912	2.925472	0.000000
11	B	2.949801	1.794333	2.938669	1.771328	1.754563

		11
11	B	0.000000

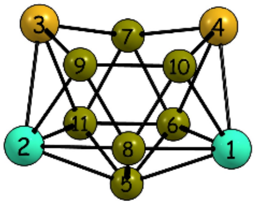

6. -8524.880786 +11.5 C_s

WBI: Co1-Co2: 0.0856

		1	2	3	4	5
1	Co	0.000000				
2	Co	3.766495	0.000000			
3	Se	4.214560	2.340529	0.000000		
4	Se	2.341366	4.212266	3.258422	0.000000	
5	B	2.125476	2.127105	3.317395	3.316724	0.000000
6	B	2.131212	3.309450	3.268717	2.268508	1.755539
7	B	3.354791	3.353902	2.039736	2.041442	2.933465
8	B	2.099169	2.098989	3.201192	3.198770	1.781727
9	B	3.339496	2.085188	2.032801	3.161545	2.954902
10	B	2.089677	3.337953	3.164467	2.032063	2.956169
11	B	3.307171	2.135129	2.273635	3.270310	1.752962

		6	7	8	9	10
6	B	0.000000				
7	B	1.819401	0.000000			
8	B	2.904493	3.481345	0.000000		
9	B	3.543975	3.143806	1.763762	0.000000	
10	B	3.083236	3.145930	1.763388	1.802661	0.000000
11	B	1.697746	1.821731	2.905686	3.085251	3.547712

		11
11	B	0.000000

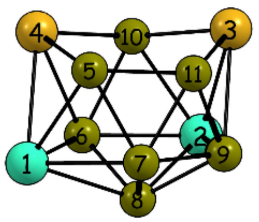

7. -8524.877325 +13.7 C_1

WBI: Co1-Co2: 0.0754

		1	2	3	4	5
1	Co	0.000000				
2	Co	3.765393	0.000000			
3	Se	4.201894	2.371075	0.000000		
4	Se	2.332788	3.650387	3.274104	0.000000	
5	B	2.149586	3.989638	3.114369	2.055569	0.000000
6	B	2.145413	2.053455	3.244705	2.232078	3.103966
7	B	2.089501	3.332654	3.194354	3.208662	1.789823
8	B	2.133114	2.109167	3.273001	3.296938	2.980425
9	B	3.319492	2.206761	2.219928	3.799800	2.913980
10	B	3.418541	2.071796	2.019532	2.054396	3.153956
11	B	3.310030	3.409727	2.038529	3.177083	1.744800

		6	7	8	9	10
6	B	0.000000				
7	B	2.915697	0.000000			
8	B	1.760493	1.785147	0.000000		
9	B	2.952821	1.714981	1.776500	0.000000	
10	B	1.873603	3.536953	2.980929	3.109527	0.000000
11	B	3.523840	1.739272	2.915232	1.811203	3.157137

		11
11	B	0.000000

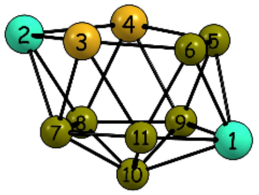

8. -8524.875701 +14.7 C_s

WBI: Co1-Co2: 0.0561

		1	2	3	4	5
1	Co	0.000000				
2	Co	4.494742	0.000000			
3	Se	3.599596	2.322219	0.000000		
4	Se	3.599964	2.321567	3.383832	0.000000	
5	B	2.074133	3.573303	3.167450	2.069567	0.000000
6	B	2.074718	3.574823	2.069455	3.169608	1.701238
7	B	3.331279	2.142374	2.109945	3.246204	3.532570
8	B	3.330960	2.139694	3.242734	2.111553	3.070031
9	B	2.020370	3.494095	3.778908	2.124493	1.905477
10	B	2.076160	3.349795	3.208635	3.210394	2.957399
11	B	2.021609	3.497329	2.125470	3.782771	2.924639

		6	7	8	9	10
6	B	0.000000				
7	B	3.070564	0.000000			
8	B	3.532587	1.794214	0.000000		
9	B	2.923543	2.954246	1.882425	0.000000	
10	B	2.957714	1.759646	1.759735	1.771848	0.000000
11	B	1.907008	1.882676	2.954812	2.889981	1.772007

11		
11	B	0.000000

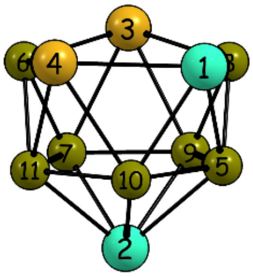

9. -8524.874663 +15.3 C_1

WBI: Co1-Co2: 0.0768

		1	2	3	4	5
1	Co	0.000000				
2	Co	3.689930	0.000000			
3	Se	3.520675	3.607378	0.000000		
4	Se	2.315676	3.611603	3.349999	0.000000	
5	B	2.064649	2.094697	3.208841	3.271444	0.000000
6	B	3.515780	3.288584	2.039241	2.034501	3.560168
7	B	3.970864	2.006858	2.159052	3.237706	2.971361
8	B	2.027922	3.299672	2.084501	3.260662	1.728957
9	B	3.313121	2.037820	2.159732	3.817557	1.758591
10	B	2.098117	2.064453	3.779050	2.159793	1.781020
11	B	3.445405	2.007193	3.236351	2.129438	2.980813

		6	7	8	9	10
6	B	0.000000				
7	B	1.821752	0.000000			
8	B	3.184670	3.082820	0.000000		
9	B	3.072679	1.920710	1.842839	0.000000	
10	B	3.076711	2.981805	2.911489	2.915559	0.000000
11	B	1.822707	1.755687	3.551495	2.965588	1.938987

		11
11	B	0.000000

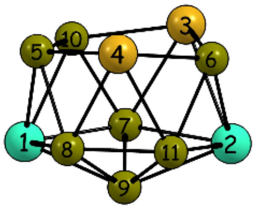

10. -8524.862010 +23.3 C_1

WBI: Co1-Co2: 0.0753

		1	2	3	4	5
1	Co	0.000000				
2	Co	3.733723	0.000000			
3	Se	3.612572	2.353311	0.000000		
4	Se	3.599638	3.622574	3.306715	0.000000	
5	B	2.072202	4.003796	3.124040	2.092254	0.000000
6	B	4.022197	2.081494	2.020308	2.044706	3.163040
7	B	2.008314	2.204722	2.191024	3.790357	2.921335
8	B	2.019483	3.363000	3.753644	2.131762	1.935693
9	B	2.065404	2.106993	3.254682	3.241752	2.989117
10	B	2.068458	3.484248	2.079756	3.167916	1.677724
11	B	3.311621	2.070209	3.235114	2.146468	3.103464

		6	7	8	9	10
6	B	0.000000				
7	B	3.108312	0.000000			
8	B	3.067270	2.890483	0.000000		
9	B	2.978054	1.789124	1.774645	0.000000	
10	B	3.170181	1.907599	2.923922	2.978106	0.000000
11	B	1.874093	2.959064	1.863622	1.756793	3.545522

		11
11	B	0.000000

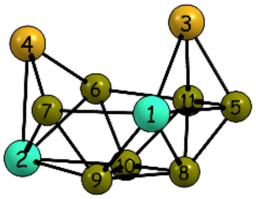

11. -8524.856795 +26.5 C_1

WBI: Co1-Co2: 0.0910

		1	2	3	4	5
1	Co	0.000000				
2	Co	3.890378	0.000000			
3	Se	2.407585	4.425845	0.000000		
4	Se	3.681730	2.310858	3.301139	0.000000	
5	B	2.115869	4.591155	1.933667	4.429426	0.000000
6	B	3.764088	2.145798	3.071205	2.004248	3.592376
7	B	2.249128	2.169595	3.288094	2.006401	3.738187
8	B	2.153357	3.393285	2.966844	4.057851	1.691706
9	B	2.154679	2.113505	3.567703	3.182770	3.093321
10	B	3.224528	2.062987	3.448604	3.157251	3.000243
11	B	2.997257	3.474811	2.121562	3.406590	1.856023

		6	7	8	9	10
6	B	0.000000				
7	B	2.849021	0.000000			
8	B	3.041031	3.065210	0.000000		
9	B	2.854408	1.731211	1.765296	0.000000	
10	B	1.719984	2.828027	1.754134	1.788568	0.000000
11	B	1.959393	3.419043	1.853192	2.843121	1.782397

		11
11	B	0.000000

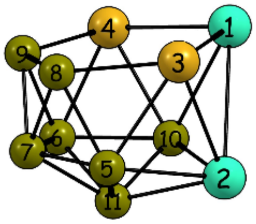

12. -8524.844227 +34.4 C_1

WBI: Co1-Co2: 0.3757

		1	2	3	4	5
1	Co	0.000000				
2	Co	2.731926	0.000000			
3	Se	2.315318	2.367744	0.000000		
4	Se	2.317219	3.805594	3.422590	0.000000	
5	B	3.664929	2.170026	2.165060	3.790607	0.000000
6	B	3.556693	3.411923	3.769253	2.061081	2.873500
7	B	4.087572	3.342781	3.203317	3.149658	1.715225
8	B	3.536914	3.447779	2.067807	3.166508	1.823073
9	B	3.530335	4.055578	3.193658	2.030902	2.888946
10	B	2.296784	2.130482	3.187168	2.162117	2.955984
11	B	3.546226	2.075110	3.212705	3.189896	1.785750

		6	7	8	9	10
6	B	0.000000				
7	B	1.751831	0.000000			
8	B	2.903636	1.752303	0.000000		
9	B	1.879749	1.749099	1.727861	0.000000	
10	B	1.917164	2.944943	3.496856	3.087225	0.000000
11	B	1.746222	1.789120	2.906359	2.920423	1.759670

		11
11	B	0.000000

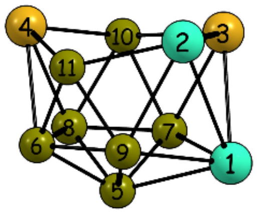

13. -8524.843352 +34.9 C_1

WBI: Co1-Co2: 0.3516

		1	2	3	4	5
1	Co	0.000000				
2	Co	2.659618	0.000000			
3	Se	2.364564	2.320521	0.000000		
4	Se	4.263909	3.429849	3.376930	0.000000	
5	B	2.099806	3.494404	3.257823	3.219644	0.000000
6	B	3.322228	3.361489	3.806181	2.164071	1.731413
7	B	2.141780	3.583662	2.180149	3.233428	1.753001
8	B	3.325164	4.011342	3.247533	2.157003	1.741204
9	B	2.116673	2.176947	3.206693	3.199737	1.825615
10	B	3.414032	3.477443	2.051022	2.046292	2.911108
11	B	3.424588	2.041246	3.317623	2.061376	2.900417

		6	7	8	9	10
6	B	0.000000				
7	B	2.914616	0.000000			
8	B	1.890077	1.716992	0.000000		
9	B	1.771812	2.942486	2.949774	0.000000	
10	B	3.046620	1.816499	1.813025	3.509286	0.000000
11	B	1.796287	3.563869	3.047278	1.726788	3.188902

		11
11	B	0.000000

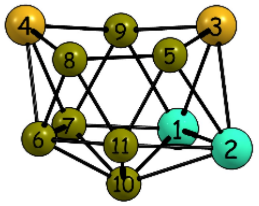

14. -8524.826761 +45.3 C_1

WBI: Co1-Co2: 0.2346

		1	2	3	4	5
1	Co	0.000000				
2	Co	2.871774	0.000000			
3	Se	2.491974	2.433257	0.000000		
4	Se	3.622198	4.333000	3.344866	0.000000	
5	B	3.648234	2.080346	2.069546	3.133254	0.000000
6	B	3.385570	3.388823	3.761420	2.125719	2.916573
7	B	2.005634	3.554391	3.262151	2.167256	3.533610
8	B	4.082546	3.299452	3.203041	2.031545	1.746113
9	B	2.043612	3.619205	2.026613	2.056830	3.122154
10	B	2.148566	2.228338	3.204549	3.264852	2.950310
11	B	3.495642	2.040540	3.201206	3.174854	1.790593

		6	7	8	9	10
6	B	0.000000				
7	B	1.836575	0.000000			
8	B	1.828866	3.030720	0.000000		
9	B	3.048180	1.896291	3.142218	0.000000	
10	B	1.808671	1.767502	2.932829	2.952707	0.000000
11	B	1.743225	2.902065	1.737666	3.524158	1.796184

		11
11	B	0.000000

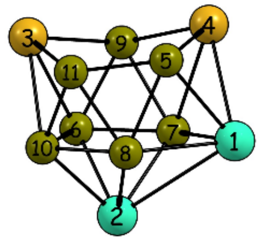

15. -8524.810808 +55.3 C_1

WBI: Co1-Co2: 0.3038

		1	2	3	4	5
1	Co	0.000000				
2	Co	2.726785	0.000000			
3	Se	4.284205	3.613720	0.000000		
4	Se	2.343290	3.834871	3.273076	0.000000	
5	B	2.098197	3.516920	3.116126	2.053468	0.000000
6	B	3.505675	1.984319	2.153650	3.239515	3.531414
7	B	2.237687	2.027857	3.282341	2.278944	3.088955
8	B	2.109700	2.136416	3.219342	3.220275	1.822016
9	B	3.442739	3.312752	2.057125	2.009070	3.115036
10	B	3.474968	2.031273	2.162566	3.811812	2.922782
11	B	3.313615	3.325376	2.059016	3.169281	1.728449

		6	7	8	9	10
6	B	0.000000				
7	B	1.756312	0.000000			
8	B	2.929746	2.838040	0.000000		
9	B	1.769170	1.873637	3.491495	0.000000	
10	B	1.968094	2.961871	1.763309	3.076321	0.000000
11	B	3.037974	3.507109	1.762559	3.151271	1.778100

		11
11	B	0.000000

Table S7: HOMO-LUMO gaps for the lowest-lying $Cp_2Co_2S_2B_{n-4}H_{n-4}$ (n = 8 to 12) structures.

Structure	HOMO (a.u.)	LUMO (a.u.)	H/L gap (a.u.)	H/L gap (eV)
B4Co2S2-1	-0.193603	-0.061574	0.132029	3.59
B4Co2S2-2	-0.225226	-0.077324	0.147902	4.02
B4Co2S2-3	-0.226228	-0.080937	0.145291	3.95
B4Co2S2-4	-0.228052	-0.090671	0.137381	3.74
B4Co2S2-5	-0.197498	-0.065590	0.131908	3.59
B4Co2S2-6	-0.223093	-0.087775	0.135318	3.68
B4Co2S2-7	-0.227306	-0.066442	0.160864	4.38
B4Co2S2-8	-0.206375	-0.071823	0.134552	3.66
B4Co2S2-9	-0.211712	-0.083840	0.127872	3.48
B4Co2S2-10	-0.202187	-0.082870	0.119317	3.25
B4Co2S2-11	-0.203823	-0.082620	0.121203	3.30
B4Co2S2-12	-0.216161	-0.074135	0.142026	3.86
B4Co2S2-13	-0.210633	-0.099043	0.111590	3.04
B4Co2S2-14	-0.198266	-0.076168	0.122098	3.32
B5Co2S2-1	-0.218297	-0.070398	0.147899	4.02
B5Co2S2-2	-0.218276	-0.082461	0.135815	3.70
B5Co2S2-3	-0.201156	-0.065249	0.135907	3.70
B5Co2S2-4	-0.217338	-0.090174	0.127164	3.46
B5Co2S2-5	-0.220130	-0.069042	0.151088	4.11
B5Co2S2-6	-0.217250	-0.072527	0.144723	3.94
B5Co2S2-7	-0.219152	-0.067803	0.151349	4.12
B5Co2S2-8	-0.212211	-0.092357	0.119854	3.26
B5Co2S2-9	-0.209982	-0.068297	0.141685	3.86
B5Co2S2-10	-0.220703	-0.088401	0.132302	3.60
B5Co2S2-11	-0.217580	-0.094390	0.123190	3.35
B5Co2S2-12	-0.197198	-0.064752	0.132446	3.60
B5Co2S2-13	-0.207107	-0.068174	0.138933	3.78
B5Co2S2-14	-0.216074	-0.086150	0.129924	3.54
B5Co2S2-15	-0.212361	-0.083945	0.128416	3.49
B6Co2S2-1	-0.233804	-0.067242	0.166562	4.53
B6Co2S2-2	-0.228579	-0.102355	0.126224	3.43
B6Co2S2-3	-0.233406	-0.100950	0.132456	3.60
B6Co2S2-4	-0.223203	-0.077404	0.145799	3.97
B6Co2S2-5	-0.225787	-0.121848	0.103939	2.83
B6Co2S2-6	-0.214510	-0.075378	0.139132	3.79
B6Co2S2-7	-0.219776	-0.069839	0.149937	4.08
B6Co2S2-8	-0.225128	-0.090105	0.135023	3.67
B7Co2S2-1	-0.217520	-0.086618	0.130902	3.56
B7Co2S2-2	-0.215292	-0.076235	0.139057	3.78
B7Co2S2-3	-0.216444	-0.077119	0.139325	3.79
B7Co2S2-4	-0.217726	-0.083778	0.133948	3.64
B7Co2S2-5	-0.222191	-0.081703	0.140488	3.82
B7Co2S2-6	-0.212882	-0.067176	0.145706	3.96

B7Co2S2-7	-0.220443	-0.065912	0.154531	4.21
B7Co2S2-8	-0.211693	-0.084588	0.127105	3.46
B7Co2S2-9	-0.218056	-0.097580	0.120476	3.28
B7Co2S2-10	-0.211145	-0.090580	0.120565	3.28
B7Co2S2-11	-0.215614	-0.089818	0.125796	3.42
B7Co2S2-12	-0.240966	-0.079079	0.161887	4.41
B7Co2S2-13	-0.213142	-0.085372	0.127770	3.48
B7Co2S2-14	-0.210504	-0.093159	0.117345	3.19
B7Co2S2-15	-0.222169	-0.081088	0.141081	3.84
B8Co2S2-1	-0.230841	-0.078452	0.152389	4.15
B8Co2S2-2	-0.227226	-0.098349	0.128877	3.51
B8Co2S2-3	-0.240416	-0.078383	0.162033	4.41
B8Co2S2-4	-0.235594	-0.101430	0.134164	3.65
B8Co2S2-5	-0.205494	-0.094582	0.110912	3.02
B8Co2S2-6	-0.234652	-0.081927	0.152725	4.16
B8Co2S2-7	-0.236901	-0.068922	0.167979	4.57
B8Co2S2-8	-0.180490	-0.087364	0.093126	2.53
B8Co2S2-9	-0.180787	-0.088298	0.092489	2.52
B8Co2S2-10	-0.214601	-0.093767	0.120834	3.29
B8Co2S2-11	-0.220388	-0.090846	0.129542	3.53
B8Co2S2-12	-0.201351	-0.093757	0.107594	2.93
B8Co2S2-13	-0.230677	-0.097051	0.133626	3.64
B8Co2S2-14	-0.230911	-0.079182	0.151729	4.13
B8Co2S2-15	-0.234166	-0.088243	0.145923	3.97
B8Co2S2-16	-0.217633	-0.100948	0.116685	3.18
B8Co2S2-17	-0.229904	-0.103103	0.126801	3.45
B8Co2S2-18	-0.222225	-0.080706	0.141519	3.85

Table S8: HOMO-LUMO gaps for the lowest-lying $Cp_2Co_2Se_2B_{n\text{-}4}H_{n\text{-}4}$ (n = 8 to 12) structures.

Structure	HOMO (a.u.)	LUMO (a.u.)	H/L gap (a.u.)	H/L gap (eV)
B4Co2Se2-1	-0.194134	-0.061880	0.132254	3.60
B4Co2Se2-2	-0.198097	-0.067298	0.130799	3.56
B4Co2Se2-3	-0.215720	-0.093983	0.121737	3.31
B4Co2Se2-4	-0.213936	-0.084262	0.129674	3.53
B4Co2Se2-5	-0.215035	-0.078019	0.137016	3.73
B4Co2Se2-6	-0.215617	-0.090279	0.125338	3.41
B4Co2Se2-7	-0.199493	-0.072065	0.127428	3.47
B4Co2Se2-8	-0.227829	-0.069917	0.157912	4.30
B4Co2Se2-9	-0.210215	-0.087839	0.122376	3.33
B4Co2Se2-10	-0.201494	-0.082562	0.118932	3.24
B4Co2Se2-11	-0.197456	-0.081864	0.115592	3.15
B4Co2Se2-12	-0.207681	-0.100980	0.106701	2.90
B4Co2Se2-13	-0.211865	-0.075440	0.136425	3.71
B4Co2Se2-14	-0.193514	-0.077775	0.115739	3.15
B5Co2Se2-1	-0.214037	-0.074428	0.139609	3.80
B5Co2Se2-2	-0.214066	-0.092177	0.121889	3.32
B5Co2Se2-3	-0.202639	-0.065636	0.137003	3.73
B5Co2Se2-4	-0.213387	-0.083838	0.129549	3.53
B5Co2Se2-5	-0.221579	-0.070584	0.150995	4.11
B5Co2Se2-6	-0.214334	-0.076923	0.137411	3.74
B5Co2Se2-7	-0.211526	-0.096174	0.115352	3.14
B5Co2Se2-8	-0.215649	-0.095719	0.119930	3.26
B5Co2Se2-9	-0.214125	-0.068531	0.145594	3.96
B5Co2Se2-10	-0.217765	-0.089851	0.127914	3.48
B5Co2Se2-11	-0.200991	-0.074914	0.126077	3.43
B5Co2Se2-12	-0.197848	-0.068186	0.129662	3.53
B5Co2Se2-13	-0.212361	-0.088737	0.123624	3.36
B5Co2Se2-14	-0.209586	-0.085502	0.124084	3.38
B5Co2Se2-15	-0.202324	-0.070736	0.131588	3.58
B6Co2Se2-1	-0.232451	-0.071727	0.160724	4.37
B6Co2Se2-2	-0.225523	-0.106265	0.119258	3.25
B6Co2Se2-3	-0.230893	-0.104112	0.126781	3.45
B6Co2Se2-4	-0.211438	-0.077848	0.133590	3.64
B6Co2Se2-5	-0.220088	-0.123549	0.096539	2.63
B6Co2Se2-6	-0.205036	-0.076200	0.128836	3.51
B6Co2Se2-7	-0.215226	-0.074701	0.140525	3.82
B6Co2Se2-8	-0.222355	-0.090804	0.131551	3.58
B7Co2Se2-1	-0.217477	-0.087247	0.130230	3.54
B7Co2Se2-2	-0.215613	-0.075296	0.140317	3.82
B7Co2Se2-3	-0.214878	-0.077504	0.137374	3.74
B7Co2Se2-4	-0.216018	-0.083725	0.132293	3.60
B7Co2Se2-5	-0.222779	-0.081742	0.141037	3.84
B7Co2Se2-6	-0.211129	-0.068406	0.142723	3.88

B7Co2Se2-7	-0.218486	-0.065628	0.152858	4.16
B7Co2Se2-8	-0.209767	-0.083517	0.126250	3.44
B7Co2Se2-9	-0.218318	-0.098240	0.120078	3.27
B7Co2Se2-10	-0.210839	-0.092582	0.118257	3.22
B7Co2Se2-11	-0.214294	-0.089686	0.124608	3.39
B7Co2Se2-12	-0.211100	-0.085699	0.125401	3.41
B7Co2Se2-13	-0.208573	-0.092739	0.115834	3.15
B7Co2Se2-14	-0.227676	-0.080417	0.147259	4.01
B7Co2Se2-15	-0.220581	-0.079863	0.140718	3.83
B8Co2Se2-1	-0.182853	-0.084385	0.098468	2.68
B8Co2Se2-2	-0.214525	-0.079182	0.135343	3.68
B8Co2Se2-3	-0.195118	-0.095794	0.099324	2.70
B8Co2Se2-4	-0.176975	-0.085967	0.091008	2.48
B8Co2Se2-5	-0.233791	-0.079401	0.154390	4.20
B8Co2Se2-6	-0.221742	-0.103330	0.118412	3.22
B8Co2Se2-7	-0.214634	-0.097983	0.116651	3.17
B8Co2Se2-8	-0.219582	-0.082076	0.137506	3.74
B8Co2Se2-9	-0.232256	-0.070722	0.161534	4.40
B8Co2Se2-10	-0.189897	-0.089322	0.100575	2.74
B8Co2Se2-11	-0.204592	-0.093773	0.110819	3.02
B8Co2Se2-12	-0.218181	-0.095221	0.122960	3.35
B8Co2Se2-13	-0.224787	-0.094744	0.130043	3.54
B8Co2Se2-14	-0.202994	-0.052846	0.150148	4.09
B8Co2Se2-15	-0.215645	-0.104850	0.110795	3.01
B8Co2Se2-16	-0.217844	-0.085570	0.132274	3.60
B8Co2Se2-17	-0.216724	-0.080198	0.136526	3.72
B8Co2Se2-18	-0.215008	-0.102803	0.112205	3.05

Table S9: HOMO-LUMO gaps for the lowest-lying permethylated $Cp_2{}^*Co_2E_2B_7H_7$ structures (E = S, Se).

Structure	HOMO (a.u.)	LUMO (a.u.)	H/L gap (a.u.)	H/L gap (eV)
B7Co2S2-1	-0.196882	-0.069681	0.127201	3.46
B7Co2S2-2	-0.196416	-0.057584	0.138832	3.78
B7Co2S2-3	-0.196686	-0.060322	0.136364	3.71
B7Co2S2-4	-0.197686	-0.067035	0.130651	3.56
B7Co2S2-5	-0.204565	-0.062148	0.142417	3.88
B7Co2S2-6	-0.196071	-0.050146	0.145925	3.97
B7Co2S2-7	-0.200641	-0.050671	0.149970	4.08
B7Co2S2-8	-0.195207	-0.066800	0.128407	3.49
B7Co2S2-9	-0.196413	-0.080657	0.115756	3.15
B7Co2S2-10	-0.194314	-0.072442	0.121872	3.32
B7Co2S2-11	-0.193032	-0.073814	0.119218	3.24
B7Co2S2-12	-0.221562	-0.062480	0.159082	4.33
B7Co2S2-13	-0.197457	-0.068250	0.129207	3.52
B7Co2S2-14	-0.190368	-0.075708	0.114660	3.12
B7Co2S2-15	-0.202632	-0.069072	0.133560	3.63
B7Co2Se2-1	-0.197144	-0.071510	0.125634	3.42
B7Co2Se2-2	-0.197562	-0.059103	0.138459	3.77
B7Co2Se2-3	-0.195696	-0.061234	0.134462	3.66
B7Co2Se2-4	-0.196573	-0.067889	0.128684	3.50
B7Co2Se2-5	-0.205886	-0.062782	0.143104	3.89
B7Co2Se2-6	-0.195044	-0.052712	0.142332	3.87
B7Co2Se2-7	-0.199369	-0.051320	0.148049	4.03
B7Co2Se2-8	-0.193869	-0.066527	0.127342	3.47
B7Co2Se2-9	-0.196908	-0.082146	0.114762	3.12
B7Co2Se2-10	-0.194676	-0.075274	0.119402	3.25
B7Co2Se2-11	-0.192322	-0.075209	0.117113	3.19
B7Co2Se2-12	-0.210876	-0.064577	0.146299	3.98
B7Co2Se2-13	-0.196035	-0.069398	0.126637	3.45
B7Co2Se2-14	-0.189696	-0.076596	0.113100	3.08
B7Co2Se2-15	-0.201949	-0.068504	0.133445	3.63
